# Extended molecular dynamics of a *c-kit* promoter quadruplex

**DOI:** 10.1093/nar/gkv785

**Published:** 2015-10-10

**Authors:** Barira Islam, Petr Stadlbauer, Miroslav Krepl, Jaroslav Koca, Stephen Neidle, Shozeb Haider, Jiri Sponer

**Affiliations:** 1 Central European Institute of Technology (CEITEC), Masaryk University, Campus Bohunice, Kamenice 5, 625 00 Brno, Czech Republic; 2 Institute of Biophysics, Academy of Sciences of the Czech Republic, Kralovopolska 135, 612 65 Brno, Czech Republic; 3 National Center for Biomolecular Research, Faculty of Science, Masaryk University, Campus Bohunice, Kamenice 5, 625 00 Brno, Czech Republic; 4 UCL School of Pharmacy, University College London, 29–39 Brunswick Square, London WC1N 1AX, UK

## Abstract

The 22-mer *c-kit* promoter sequence folds into a parallel-stranded quadruplex with a unique structure, which has been elucidated by crystallographic and NMR methods and shows a high degree of structural conservation. We have carried out a series of extended (up to 10 μs long, ∼50 μs in total) molecular dynamics simulations to explore conformational stability and loop dynamics of this quadruplex. Unfolding no-salt simulations are consistent with a multi-pathway model of quadruplex folding and identify the single-nucleotide propeller loops as the most fragile part of the quadruplex. Thus, formation of propeller loops represents a peculiar atomistic aspect of quadruplex folding. Unbiased simulations reveal μs-scale transitions in the loops, which emphasizes the need for extended simulations in studies of quadruplex loops. We identify ion binding in the loops which may contribute to quadruplex stability. The long lateral-propeller loop is internally very stable but extensively fluctuates as a rigid entity. It creates a size-adaptable cleft between the loop and the stem, which can facilitate ligand binding. The stability gain by forming the internal network of GA base pairs and stacks of this loop may be dictating which of the many possible quadruplex topologies is observed in the ground state by this promoter quadruplex.

## INTRODUCTION

Guanine rich DNA and RNA sequences can self-assemble to form non-canonical secondary and tertiary structures termed G-quadruplexes (GQs). The underlying motif of these structures is a Hoogsteen bonded planar arrangement of four guanines, the G-quartet (1–5). Several G-quartets stack together and intervening nucleotides are looped out to form a GQ (1,2,5). Interest in these structures has increased in the last fifteen years due to their potential presence in gene regulatory sites such as gene promoters, 5′- and 3′-UTRs, telomeres, breakpoint regions, immunoglobulin heavy chain switch and hypervariable regions (1,3,6–12). The presence of GQs in promoter sequences is noteworthy as these can be involved in selective gene regulation at the transcriptional level and are potential targets for therapeutic intervention in diseases such as cancer (8,13–15). Promoter GQ stabilization by small-molecule ligands offers an alternative to direct drug targeting, of for example oncogenic kinase protein active sites, which has often resulted in severe clinical problems of resistance to these drugs, usually via active-site mutations (16–19). GQ-stabilizing small molecules have been shown for example to downregulate the expression of the *c-myc* oncogene (20–23). A GQ interacting compound quarfloxin has undergone phase II clinical trials in patients with tumours originating from neural crest cells (14,24).

The activating mutations in the *c-kit* proto-oncogene have been observed in several types of human malignancy, notably gastrointestinal tumours (GIST), systemic mastocytosis and subsets of acute myeloid leukemia and melanoma (25,26). The kinase-targeting drug Imatinib destabilises the active conformation of the c-kit kinase domain and is the mainstay of current treatment for GIST and mesenchymal tumours. Subsequent resistance to Imatinib therapy is frequently encountered in many patients (27,28). Therefore, alternative methods to achieve c-kit deactivation and circumvent resistance are currently being explored. One such approach could be selective and direct *c-kit* inhibition of transcription via GQ formation at the promoter sequence level (29).

**Figure 1.**
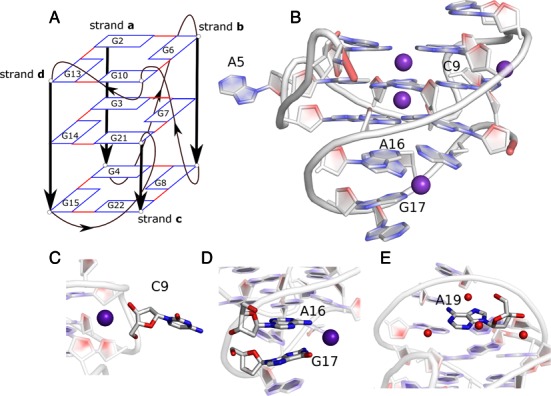
Structure of the *c-kit* promoter GQ. The 22-mer sequence (AG_3_AG_3_CGCTG_3_AGGAG_3_) forms a GQ with non-G-tract base G10 in the G-stem. The stem is supported by four loops; A5 and C9 single residue propeller loops, the C11T12 lateral loop and the A16-G20 long lateral-propeller (LP) loop. The guanine bases of the stem are in an *anti-*orientation. (**A**) Schematic representation of the *c-kit* promoter GQ. The guanine bases of the stem are shown as rectangles and the backbone is shown by thick black lines. The loops are shown by thin black lines and the arrows indicate 5′-3′ direction. The strands **a**, **b, c** and **d** are labelled as described in the text. (**B**) Cartoon representation of the crystal structure of the *c-kit* promoter GQ used as the starting structure in the present simulations. K^+^ ions are shown as purple and water molecules as red spheres. Additional K^+^ ions are bound to the exterior of the GQ in the crystal structure (**C**) in loop C9 and (**D**) between A16 and G17 of the LP loop. (**E**) Structural water molecules are present near A19 in the LP loop. The backbone of A1 is shown in red; hydrogen atoms are not shown in the figure.

The 22-mer sequence d(AG_3_AG_3_CGCTG_3_AGGAG_3_), 87 nucleotides upstream of the transcription start site of the human *c-kit* gene, forms a stable GQ, whose 3D structure was initially elucidated by NMR methods in the presence of K^+^ (30). It revealed that this *c-kit* promoter GQ adopts a unique scaffold where rather than all guanines in the G-tracts participating in quartet core formation, a non-G-tract guanine was also involved (Figure 1A). The terminal guanines of the sequence insert to complete the quartets and form a snapback scaffold. All the stem guanine bases are in *anti*-conformations, implying that it is a parallel stranded GQ. It includes four loops that connect the quartets; two single residue propeller loops, one two-residue lateral loop and one long and structured lateral-propeller (LP) loop of five residues. The unprecedented topology of this *c-kit* promoter GQ was subsequently confirmed by several X-ray crystal structures, also obtained in the presence of K^+^ (Figure 1) (31,32). To date this is the sole promoter GQ for which both NMR and crystal structures are available: these studies concur in showing a single folded arrangement and demonstrate the robustness of this *c-kit* promoter GQ topology. The first crystal structure to be reported revealed the presence of well-conserved non-channel ions and water molecules within the loops (Figure 1) (31). These ions are in virtually identical positions in the two molecules in the crystallographic asymmetric unit, indicating their potential role in maintaining loop structural integrity.

The uniqueness of this *c-kit* promoter GQ structure makes it a potential target for drug design in cancer therapy (33–36). Previous bioinformatics analysis has revealed that this 22-mer sequence has only a single occurrence in the human genome and sequences closely similar are exceptionally rare (37). The NMR and X-ray data provide invaluable insights into the structure of this *c-kit* promoter GQ. However, the experimental structures inherently provide static descriptions, opening a space for complementing the experiments by explicit solvent molecular dynamics (MD). MD has been a valuable tool to explore structural dynamics of diverse GQ molecules (38–64). Insight into structural dynamics can be useful in identifying druggable sites in the molecule (65).

In this study, we use MD techniques to evaluate the structural stability, ion interactions and loop dynamics of the *c-kit* promoter GQ structure on an extended time-scale. The several unique structural features of this GQ have been previously studied by MD simulations, such as the G-stem with a discontinuous G-strand, single-nucleotide propeller loops and the long LP loop, but not on an extended time-scale (32). Previous MD studies on GQs have shown that the G-stems are well described by the available force-fields and, due to their extraordinary stiffness, relatively short simulations are sufficient to obtain a basically converged picture of their structural dynamics (41,42,47,66). By contrast, the flexible loops of GQs represent a challenge. Shorter simulations are insufficient to provide a satisfactory sampling of the loops and, in addition, the force-field description of at least some of the loops is imperfect (41–43,46,48,50,67). Thus, our simulations were expanded to 10 μs, which to our knowledge are the longest trajectories reported to date on any GQ. Our study provides more general insights into GQ loop behaviour and applicability of the MD technique to DNA GQs, as well as information about the structural dynamics of the *c-kit* promoter GQ. We have also used the study to examine the robustness of the available force-fields, for which the *c-kit* promoter GQ, as the best-determined of all available GQ structures, is an ideal test-bed.

## MATERIALS AND METHODS

### Starting structure

The structure of this *c-kit* GQ has been previously elucidated by NMR spectroscopy (PDB code: 2O3M) and X-ray crystallography (PDB code: 3QXR, 4WO2 and 4WO3) (30–32). As the resolution of one particular X-ray structure 3QXR (1.62 Å) is superior to the other structures, we used this as the starting point for all our simulations (31). Subsequent crystal structures confirmed the uniqueness of the fold (32). All the structures have identical topologies and using only one of them as the starting structure is entirely justified. The 3QXR crystal structure contains two GQ molecules (A and B) in the asymmetric unit. They are closely similar except for differences in the conformations of residues A1, C11 and ^Br^U12 at their stacking interfaces. We have taken the coordinates of 3QXR quadruplex B as the starting structure for all our simulations, since the orientation of C11 in this structure is similar to the NMR structure. The ^Br^U12 nucleoside was replaced by T12. Nevertheless, our simulations should be long enough to sample the conformational space of short unstructured loops independent of the starting structures.

### Water and ionic conditions in standard simulations

The starting crystal structure showed two K^+^ ions occupying the G-stem channel, and two K^+^ bound to the exterior of the GQ. These additional ions interact with the propeller loop formed by the single residue C9 and the LP loop residues A16 and G17 (Figure 1). We carried out separate simulations in the presence of both Na^+^ and K^+^ to study ion-dependent effects on the structure of the GQ. In simulations with Na^+^, the structural K^+^ ions in the crystal structure were replaced by Na^+^ in the starting structure. Solvent molecules and additional ions were added using the xleap module of AMBER12 (68). The system was first neutralized by Na^+^ (or K^+^) and then excess NaCl (or KCl) of 0.15 M concentration was further added to the system. We used Joung and Cheatham parameters for Na^+^ (radius 1.212 Å and well depth of 0.3526418 kcal mol^−1^), K^+^ (radius 1.593 Å and well depth 0.4297054 kcal mol^−1^) and Cl^−^ (radius 2.711 Å and well depth 0.0127850 kcal mol^−1^) (69). The system was solvated with the SPC/E water model and placed in a truncated octahedral box with minimal distance of 10 Å of solute from the box border. Our basic simulations were extended to 10 μs.

### Simulations under low-salt and no-salt conditions

We also carried out simulations where the DNA molecules were not fully neutralized by the ions. In this particular case, the overall neutralization of the system (which is necessary to apply the standard periodic boundary condition) was achieved by net-neutralizing plasma, i.e., compensatory charge was equally distributed over all particles in the simulation box. These calculations were initially carried out with the TIP3P water model. TIP3P somewhat kinetically accelerates structural changes compared to the SPC/E model, although we do not expect that the choice of the water model has any significant effect on the simulation outcome (49). However, following a referee's suggestion, three additional no-salt simulations have been carried out with the SPC/E water model to rule out any water model-based bias. In some simulations, we included two K^+^ ions in the channel while in other simulations all the ions were excluded (true no-salt simulation).

The purpose of these simulations was to destabilize the molecules and to eventually capture early stages of unfolding under the low or no-salt condition. Then, partially unfolded structures were chosen to attempt folding back into the native structure by adding the ions. In these refolding attempts, K^+^ ions were added using the xleap module of AMBER12 at places with low electrostatic potential calculated by CMIP (70). The structures were neutralized using appropriate number of K^+^ (radius 1.705 Å and well depth 0.1936829 kcal mol^−1^, TIP3P-specific parameters) and solvated in TIP3P solvent (69). Excess KCl up to 0.15 M concentration was then again added and 100 ns–2 μs long standard simulation runs were carried out after equilibration steps (as described in the Supplementary Data section). The basic idea of this technique (no salt simulation followed by standard simulation) is that it to some extent resembles stop-flow experiments. The folded GQ molecule is first exposed to a denaturing chemical (low or no-salt) condition and after some perturbation is achieved, reparation of the structure is attempted. This approach has been recently introduced as an efficient tool to obtain atomistic insights into early stages of unfolding and potential late stages of folding of GQ molecules (38). The method is justified in more detail at the end of the Discussion and in (38). Further, the outcome of no-salt simulations does not depend on defining any biasing ‘coarse-graining’ collective variables (reaction coordinate) which other types of enhanced-sampling simulations (such as metadynamics or steered dynamics) must utilize to drive the movements and which may significantly bias the unfolding processes (71).

### DNA force-field

The simulations were carried out with the Cornell *et al*. force-field basic version parm99 with parmbsc0 refinement, which is essential to obtain stable DNA trajectories (72). It was supplemented by the recent parmχ_OL4_ refinement (49). This force-field version is designated as bsc0χ_OL4_ throughout the text. Bsc0χ_OL4_ has been shown to improve the behaviour of simulated DNA GQs compared to simulations carried out with the bsc0 refinement alone (49). The χ_OL4_ tweak improves the shape of the χ *syn* region and the *syn*-*anti* balance (38). An additional 10 μs simulation was carried out by further adding the latest ϵζ_OL1_ refinement (73), abbreviated as bsc0χ_OL4_ϵζ_OL1_. The ϵζ_OL1_ refinement corrects the ϵ = g+ region and improves the barrier between B_I_ and B_II_ B-DNA conformations. The ϵζ_OL1_ parameters have been, for example shown to markedly improve the description of B-DNA (its helical twist as well as B_I_/B_II_ populations), GQ stems (73) and catalytic centres of ribozymes (74). The χ_OL4_ and ϵζ_OL1_ corrections are fully endorsed in the latest versions of the AMBER code AmberTools for DNA simulations; for an overview of these modifications see (75). Note that the above dihedral potential corrections are intentionally constructed to be mutually compatible and additive, so it is not necessary to apply all of them simultaneously.

### MD simulations

Standard equilibration protocols were used (for a detailed description see the Supplementary Data section). The final MD simulations were performed with the PMEMD CUDA version of AMBER12 (68,76,77). The periodic boundary conditions were defined by the PME algorithm and the non-bonded cut-off was set to 9 Å (78). Covalent bonds involving hydrogen atoms were constrained using the SHAKE algorithm with a tolerance of 0.0001 Å, allowing integration time step of 2 fs (79). All simulations were carried out at a constant pressure of 1 atm and a constant temperature of 300 K. The temperature and pressure were maintained using a Berendsen weak coupling thermostat (80). Typically, the frames were written at every 10 ps, so the 10 μs trajectory analyses are based on 10^6^ datapoints (to simplify the Figures, the graphs were prepared using only each 10th snapshot). Analyses of trajectories were performed using the ptraj module of AMBER (81) and the VMD program was used for visualization (82). The programs CHIMERA (www.cgl.ucsf.edu/chimera) (83) and PyMOL (www.pymol.org) (84) were also used for visualization. A list of all simulations is given in Table 1.

**Table 1.** List of simulations presented in this paper^a^

Simulation number	Starting structure	DNA force-field	Ions used in the simulation^b^	Length of the simulation
1.	3QXR	bsc0χ_OL4_	0.3 M Na^+^ and 0.15 M Cl^−^	10 μs
2.	3QXR	bsc0χ_OL4_ϵζ_OL1_	0.3 M Na^+^ and 0.15 M Cl^−^	10 μs
3.	3QXR	bsc0χ_OL4_	0.3 M K^+^ and 0.15 M Cl^−^	10 μs
4.	3QXR	bsc0χ_OL4_	Two channel K^+^	1.25 μs
5.	Starting structure taken at 280 ns of Simulation **4**	bsc0χ_OL4_	0.3 M K^+^ and 0.15 M Cl^−^	300 ns
6.	3QXR	bsc0χ_OL4_	No ions (TIP3P water model)	500 ns
7a-i.	Perturbed GQ structures taken at 9, 59 (two attempts), 78,132,160, 255, 348 and 424 ns of Simulation **6**	bsc0χ_OL4_	0.3 M K^+^ and 0.15 M Cl^−^ (TIP3P water model)	Nine simulations totaling 3.35 μs
8.	3QXR	bsc0χ_OL4_	No ions (TIP3P water model)	500 ns
9a-g.	Perturbed GQ structures taken at 32, 100, 284, 327, 394, 453 and 479 ns of Simulation **8**	bsc0χ_OL4_	0.3 M K^+^ and 0.15 M Cl^−^ (TIP3P water model)	Seven simulations totaling 7.7 μs
10a-c.	3QXR	bsc0χ_OL4_	No ions (TIP3P water model)	Three simulations totaling 215 ns
11a-c.	3QXR	bsc0χ_OL4_	No ions	Three simulations totaling 450 ns

^a^Some additional simulations (∼6 μs in total), are not shown in the Table but are briefly discussed in the ‘Discussion’ section.

^b^SPC/E water model, unless specified otherwise.

### Selected abbreviations

Guanines G2, G3 and G4 are referred to as strand **a**, G6, G7 and G8 as strand **b**, G10, G21 and G22 as strand **c** and G13, G14 and G15 as strand **d**. The first quartet refers to the quartet closest to the 5′ end formed by G2, G6, G10 and G13. The second quartet refers to the middle quartet formed by G3, G7, G21 and G14 and the third quartet is formed by G4, G8, G22 and G15. Although we used excess salt conditions (see above), for the sake of simplicity we mark the simulations as Na^+^ and K^+^.

## RESULTS

Despite continuous tuning of force-fields for simulating non-canonical DNA structures such as GQs, the simulation force-fields remain only approximations (39,49). Evaluation of the performance of the simulation methodology was therefore one of our goals. The standard DNA simulation force-field since 2007 has been the parmbsc0 version of the Cornell *et al*. AMBER force-field which has provided a decisive stabilization of DNA simulations (41,50,66,72). Recently, the χ_OL4_ extension of this force-field has shown improvement in the handling of GQs (49). Therefore, we used the bsc0χ_OL4_ force-field for the majority of our simulations. We monitored all backbone dihedrals to confirm that the dihedral angles are in the range of the experimentally observed values (for details see the Supplementary Data section, including Supplementary Figures S1–S6). To increase sampling and to obtain insights into potential sensitivity of the results to force-field adjustments, an additional 10 μs simulation was carried out by adding the most recent B-DNA specific ϵζ_OL1_ modification (bsc0χ_OL4_ϵζ_OL1_ version; see the Methods section) (73).

The NMR and X-ray structures of *c-kit* promoter GQ have been obtained in the presence of K^+^ and no structure in Na^+^ is available. However, two of our three reference 10 μs simulations (Table 1) have been performed in the presence of Na^+^ excess salt. A detailed explanation of the ion-choice in the GQ simulations is presented at the end of the Discussion.

### 10 μs bsc0χ_OL4_ Na^+^ simulation (Simulation 1)

**Figure 2.**
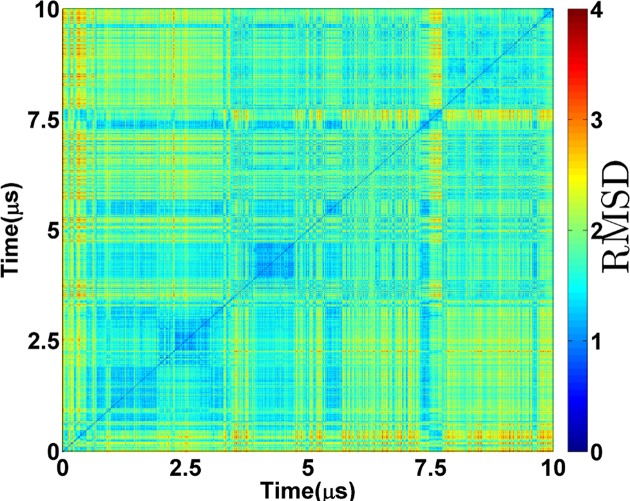
2D-RMSD of backbone atoms of the *c-kit* promoter GQ over 10 μs in Simulation **1** carried out in excess Na^+^ with the bsc0χ_OL4_ force-field. The RMSD scale is shown by the colour bar. The Figure visualises the data with δ*t* = 5 ns resolution.

**Figure 3.**
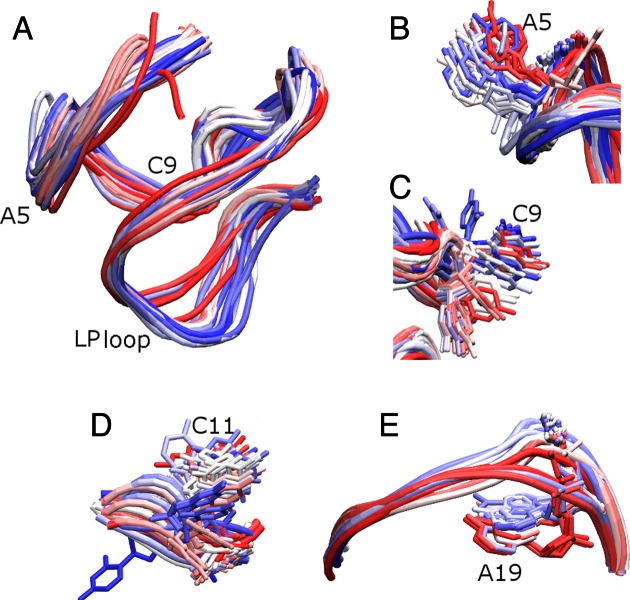
Dynamics of the *c-kit* promoter GQ in the 10 μs long simulation in excess Na^+^ carried out with the bsc0χ_OL4_ force-field (Simulation **1**). The different conformations are coloured according to simulation progression; the structure at the beginning of the trajectory is red, the middle in white and last one in blue. The conformations are sampled at a time step of 200 ns. (**A**) Overlay of backbone conformations of the GQ sampled over the simulation. The most flexible residues of the GQ (**B**) A5, (**C**) C9, (**D**) C11 and (**E**) A19 are shown in liquorice representation while the backbone is shown in tube representation.

The 2D- root-mean-squared coordinate deviation (RMSD) analysis of backbone atoms of the *c-kit* promoter GQ in Simulation **1** shows that the trajectory is dynamic over the entire 10 μs (Figure 2, Supplementary Figure S7). The guanine bases in the G-stem, the overall conformation of the GQ and the channel ions are stable during the entire simulation (Figure 3A, Supplementary Figure S8). However, dynamic changes occur in the loop regions (Figure 3).

The bases in the single residue propeller loops A5 and C9 were flexible but maintained the backbone conformation of the loops throughout the simulation (Figure 3B and C). In the lateral loop, C11 was in an *anti*-conformation in the starting structure but visited *syn* conformations via infrequent and short-lived flips (Figure 3D & Supplementary Figure S5). T12 is less flexible as it was stacked over G13 of the first quartet and was also stabilized by its interaction with the terminal A1 base (see below). The LP loop remained internally firmly structured and stable but showed profound overall fluctuations of its position with respect to the stem, namely, to its adjacent third quartet (Supplementary Figure S9). The loop position similar to the starting structure was observed after ∼7.5 μs of the simulation (Figure 3E). This is also evident in the RMSD-based clustering analysis of the trajectory as the cluster closest to the crystal structure reappeared briefly at ∼2.2 and 7.5 μs of the simulation time (Supplementary Figures S10 and S11, Supplementary Table S1). The backbone RMSD of the medoid representing this cluster from the crystal structure B of 3QXR, is 1.43 Å. Further details of the clustering analysis are presented in the Supplementary Data section.

**Figure 4.**
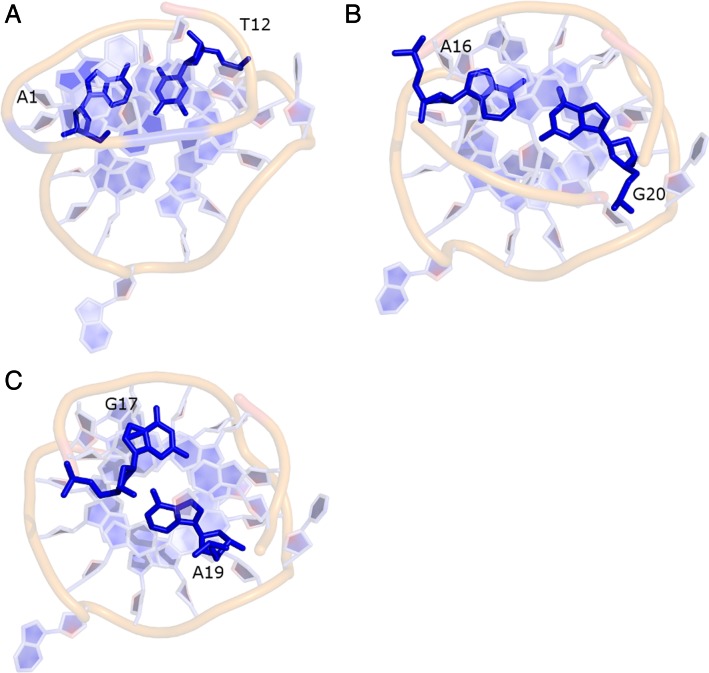
Base pairings in the *c-kit* promoter GQ that has been predominantly sampled in the simulations. (**A**) A1 and T12 ultimately form a *cis* Watson-Crick pair, (**B**) A16 and G20 form a Watson–Crick base pair and (**C**) G17 and A19 form a sheared base pair throughout the simulation. The first base pair is seen in the NMR structure while the other two base pairs are present in both the crystal and NMR structures.

The simulation sampled the base pairing patterns observed in the experimental structures (Figure 4). The terminal base A1 flipped from the X-ray *anti* orientation to *syn* after 2 μs and formed a *cis* Watson-Crick (WC) base pair with T12 of the lateral loop (Figure 4A, Supplementary Figure S12a). This rearrangement is in agreement with the NMR structure which shows A1 in a *syn* conformation. In the crystal structure, A1 may be stabilized in an *anti*-conformation by crystal packing interactions. The A1 *syn* conformation was stabilized by an intramolecular H-bond between the 5′ OH terminal group and N3(A1), which is known to be a powerful *syn*-stabilizing interaction for 5′-terminal purine nucleotides (45,85). In the LP loop, WC base pairing between A16 and G20 was similar to that in the crystal and NMR structures (Figure 4B). This base pairing was observed most of the time except between ∼7.9–8.2 μs and ∼8.7–9.0 μs (Supplementary Figure S12b). G17 and A19 formed a sheared base pair in the experimental structures (Figure 4C). This base pair remained stable in the simulation although the bases were staggered during some parts of the trajectory (Supplementary Figure S12c). The successful force-field description of the long LP loop may be at first sight surprising, considering the literature reports of difficulties in MD descriptions of the GQ loops (48,50). However, the LP loop is highly structured which may help to stabilize it during the MD, at least kinetically.

### 10 μs bsc0χ_OL4_ϵζ_OL1_ Na^+^ simulation (Simulation 2)

The G-stem of the GQ was again stable (Supplementary Figure S13). The terminal residue A1 was initially in an *anti*-orientation and stacked over the first quartet to form a reverse Hoogsteen base pair with T12. At ∼4.8 μs the A1 base flipped to a *syn* orientation and then permanently formed the *cis* WC base pair with T12 (as in Simulation **1**, Supplementary Figure S14a). As in Simulation **1**, the A16-G20 and G17-A19 base pairs were stable (Supplementary Figure S14b and c).

**Figure 5.**
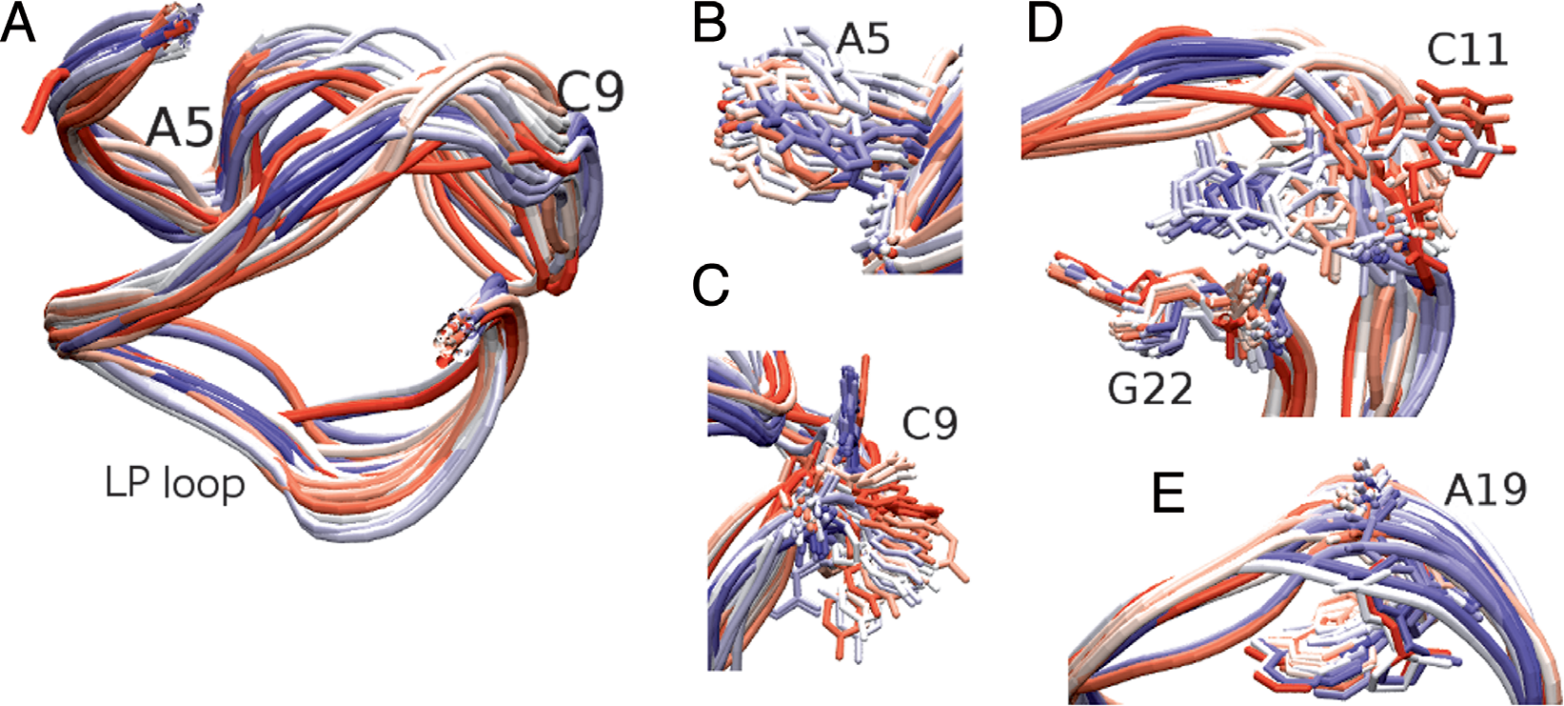
Dynamics of the *c-kit* promoter GQ in the 10 μs long Simulation **2** in excess Na^+^, carried out with the bsc0χ_OL4_ϵζ_OL1_ force-field. The different conformations are coloured according to the simulation progression as in Figure 3. The conformations are sampled at a time step of 200 ns. (**A**) Overlay of backbone conformations of the GQ, (**B**) A5 dynamics, (**C**) C9, (**D**) C11 and G22 dynamics showing insertion of the C11 into the groove fixed by a hydrogen bond between the amino group of C11 and O4′ of G22 in 6.8–10 μs and (**E**) A19.

The 2D-RMSD analysis of backbone atoms indicated that the GQ became trapped from ∼6.8 μs till the end (10 μs) in a specific conformation which had a RMSD of ∼3 Å from the starting structure and was not observed in Simulation **1** (Supplementary Figure S15). We identified its origin in the loop region. The single residue propeller loops A5 and C9 retained their conformations throughout the simulation (Figure 5). The LP loop sampled conformations similar to the NMR and X-ray structures throughout the simulation. All these dynamics were consistent with Simulation **1**. However, the lateral loop C11 residue flipped into a *syn* orientation at 6.8 μs and became inserted in the groove between strands **c** and **d**. The C11 base then formed a hydrogen bond with O4′ of G22 in the groove through its –NH_2_ group and the structure remained locked in this position until the end of the simulation (Figure 5D and Supplementary Figure S14d). This arrangement was not seen in the experimental structures. Similar non-canonical interactions between bases and grooves are often observed during end-fraying of B-DNA duplexes in simulations (86). Interestingly, the most recent crystal structure of the *c-kit* promoter GQ (PDB id: 4WO2) reveals that T12 of the same loop interacts with the backbone of G22, which was not observed in the earlier experimental structures (32). Therefore, formation of the C11 groove interaction may simply reflect different sampling in the two simulations and is not necessarily a result of force-field differences. It can even represent a realistic substate, though we cannot rule out that it is also a force-field imbalance. We did not find any unusual backbone conformations which would explicitly indicate that adding the modified ϵζ_OL1_ parameters was responsible for this behaviour, though this did not rule out a more subtle (indirect) force-field influence. The simulation time scale was not sufficient to tell if the C11–backbone interaction is the global minimum by the force-field or if it is just a rare long-living fluctuation accidentally reached by one particular simulation. Resolving these issues would require at least an order of magnitude longer time scales for the simulations.

### 10 μs bsc0χ_OL4_ K^+^ ions simulation (Simulation 3)

The 2D-RMSD analysis of the 10 μs long K^+^ trajectory revealed that the backbone conformation of the *c-kit* promoter GQ deviated marginally more from the starting structure compared to the equivalent Na^+^ Simulation **1** (Supplementary Figure S16). The C11 of the lateral loop moved over to the helix terminal and stabilized itself by forming short-lived interactions with either T12 or the G10 backbone. Similar behaviour was also observed in Simulation **1**. The other base pairs behaved similarly as in Simulation **1** (Supplementary Figure S17). The LP loop was perhaps slightly more flexible in the K^+^ than in the Na^+^ simulation (Supplementary Figure S18). This, however, may still be a sampling issue. Thus, there is no indication of any systematic difference between our Na^+^ and K^+^ 10 μs simulations (cf. also discussion in (38)).

### Cations bind to the exterior of the *c-kit* promoter GQ

The high-resolution crystal structure of the *c-kit* promoter GQ suggested that non-channel cations may be important to maintain its structure (31). However, not all ions visualized by X-ray crystallography are structurally important and, in addition, these quasi-static experimental structures do not provide significant dynamic information beyond crystallographic B-factors (87). Conventional time-scale X-ray crystal structures may miss important ion binding sites if they are delocalized, as exemplified by the prominent ion binding pocket of the HIV-1 dimerization initiation site RNA kissing-loop complex in the presence of monovalent ions (88). MD simulations provide a robust alternative tool to study structurally important monovalent ion binding sites (87–95). The simulations also usually provide successful indirect predictions for binding of divalent ions (87,88,92,96), which are much more challenging for direct simulation analysis. Several major ion binding sites in folded RNA molecules have been characterized in the past by MD simulations (91,92,97,98). Potential ion binding to the minor groove of B-DNA A-tracts has also been intensely studied, though its actual importance remains unclear (99,100). Essentially all nucleic acids molecules are surrounded by many ion binding sites with highly variable arrangements, dynamics, binding times and occupancies. Nevertheless, only a small fraction of them is likely to be sufficiently important to specifically affect the folding and other properties of the molecules. Earlier nucleic acids MD studies have suggested that important monovalent ion binding sites should have essentially 100% occupancies with either direct (inner-shell) ion binding or 100% presence of ions in some wider ion-binding pockets, which may sometimes include simultaneously bound multiple ions (87,88,92).

**Figure 6.**
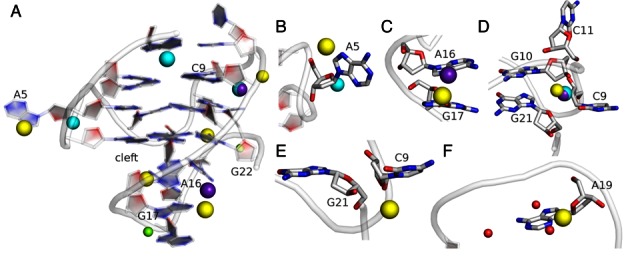
Comparison of cation binding pockets as observed in Simulation **1** and in the crystal structure (PDB id: 3QXR) of the *c-kit* promoter GQ. The K^+^ ions as observed in GQs A and B of the crystal structure are shown in cyan and purple, respectively. The X-ray Mg^2+^ ions are shown in green while the structural water molecules are shown in red. The Na^+^ ions in the simulation are shown in yellow. (**A**) Cartoon representation of the GQ structure in the simulation at ∼4 μs. The ion positions in the crystal structure were overlaid for comparison on the simulation structure using the *Chimera* program. (**B**) In the A5 loop, a Na^+^ ion is present between N7 and backbone of A5 while in the 3QXR structure A, it bridges between O4′ of A5 and the backbone of G7. (**C**) The cation is present between A16 and G17 in the LP loop. (**D**) and (**E**) The phosphate cluster of C9, G10, C11 and G21 is shielded by one crystallographic K^+^ ion while in the simulation two Na^+^ ions are present. (**F**) Cations bind to N3 of A19 in the simulations and impart flexibility to the LP loop.

In our simulations, we have first identified the ion binding sites via density analysis and then investigated their details (Table 2, Supplementary Figure S19 and Supplementary Table S2). We have observed that cations are coordinated with all loops to a certain extent, in broad accordance with the findings in the high-resolution crystal structure (Figure 6 and Supplementary Figure S20) (31). The most important ion binding site predicted by the simulations forms around the phosphates of the propeller loop C9 and adjacent G21. The occupancy of the pocket is ∼50–75% (Table 2 and Supplementary Table S2) with typical ion presence (binding) times in the range 0.01–1 μs. The reason for formation of this binding site may be the proximity of the G21 phosphate to C9 as the LP loop has a turn here in order to insert G21 and G22 into the quartet core. The C9 propeller loop may be stabilized by a cation bridging between the phosphate oxygen atoms of C9 and G21 (Figure 6E and Supplementary Figure S20c). Interestingly, this ion binding site has not been predicted by the crystal structure; however, since the simulations do not reveal a fully immobilized ion this is not surprising. Another cation binding site forms at the interface of the backbone atoms of C11, G10 and G21 (Figure 6D and Supplementary Figure S20d). It can stabilize the structure of the lateral loop and also shield the electrostatic repulsions between G10 and G21 backbone atoms. In the crystal structure 3QXR, a single cation is in contact with both the propeller (C9) and lateral (C11 and T12) loops. This cation directly binds to C11, G10 and G21 residues and is connected by a water network to the single-residue propeller loop C9.

**Table 2.** Summary of Na^+^ binding sites in the *c-kit* promoter GQ observed in Simulation **1^a^**

Residues	Atoms of the GQ forming the cation binding site	Binding time (ns)	Occupancy during the simulation (%)	Observed in the crystal structure
C9	Phosphate oxygen atom of C9 and G21	2–930	73	No
C11, G10 and G21	C11, G10 and G21 atoms at their sugar phosphate backbone interface	1–100	30	Yes
A5	N7 of A5 and sugar phosphate backbone of A5	1–70	15	No
A16	N7 of A16 and carbonyl oxygen of G17	1–100	19	Yes
A19	N3 of A19	1–60	13	No

The percent occupancy was calculated by dividing the number of frames in which any Na^+^ ion is at or below a 3.0 Å distance from the respective site, by the total frames of the trajectory multiplied by 100.

^a^See Supplementary Table S2 in Supplementary Data section for Simulations **2** and **3**.

We also observe that N3 of A19 is a potential cation binding site as it is exposed to the cleft between the LP loop and the adjacent third quartet (Figure 6F, Supplementary Figure S20f). This site has low total occupancy which, however, depends on the sampled conformation. When a cation binds to N3(A19), the entire loop bends as a rigid body towards the adjacent quartet. This decreases the cleft space between the LP loop and the adjacent quartet (Supplementary Figure S21). We suggest that these loop conformations are competing sub-states with dynamical involvement of ion binding (Supplementary Figure S22). We have attempted a MM-PBSA free energy computation but this did not show (or was not sensitive enough to capture) any significant energy difference between the two loop positions (described in the Supplementary Data section).

We have also identified some other potential non-channel cation-binding sites (Figure 6 and Supplementary Figure S20). In the Na^+^ ion Simulation **1**, the A5 propeller loop interacted with a cation coordinated between its N7 and phosphate oxygen atom, although in the K^+^ ion Simulation **3** and crystal structure, the A5 propeller loop interacted with a cation coordinated instead between its O4′ atom and phosphate oxygen of the G7 residue (Figure 6B and Supplementary Figure S20b). In the LP loop, cation coordinated between adjacent bases A16 and G17 in both Simulations **1** and **3**, similar to the crystal structure (Figure 6C and Supplementary Figure S20e). In Simulation **2** along with ion binding sites consistent with those seen in Simulations **1** and **3**, an additional ion binding site was observed between the flipped C11 and backbone of G13 (Supplementary Table S2). This cation shielded the repulsion of C11 from the backbone of G13 after the C11 flip.

On considering the occupancies we suggest that only the first two above-reported cation binding sites and the sub-state-specific N3(A19) site are important. The remaining binding sites show lower occupancies even though some of their individual ion binding events are long (1–50 ns), at least compared to the literature data (87,88,92). However, when comparing with the earlier MD studies of structurally bound monovalent ions, it is important to note that most of them were typically done using ∼10 ns simulations, which were shorter than many of the individual binding events detected by our 10 μs trajectories. Nevertheless, the older studies usually identified the major binding sites based on their consistent formation in multiple independent short trajectories, which should eliminate eventual bias due to long but rare individual binding events (87,88,92).

### *c-kit* promoter GQ simulations with only channel cations (Simulations 4 and 5)

Based on our simulations and comparison with the crystal structure, it is clear that the loops in the *c-kit* promoter GQ are cation-binding sites. To explore the role of additional ions in maintaining its structure, we simulated the GQ with only two channel cations (limited-salt Simulation **4**). The channel ions should be sufficient to keep the GQ stable but the lack of bulk ions eliminates any ion stabilization of the loops. The G-stem was indeed stable for the whole simulation, reflecting the known major role of the channel ions in stabilizing GQs. Nevertheless, the RMSD values reached ∼3.25 Å within 10 ns (Supplementary Figure S23). The GQ was inflexible for the remaining time of the simulation and did not re-attain the starting structure (Supplementary Figures S23 and S24). The structural difference compared to the start was caused by expansion of the cleft between the LP loop and the adjacent quartet by ∼8 Å, within the first 3 ns of the simulation (Supplementary Figure S24a). We did not observe any significant decrease in cleft size except for a 5 ns fluctuation at ∼220 ns. We then used the structure obtained after 280 ns of the limited-salt simulation to start a standard K^+^ simulation (Simulation **5**). The cleft between the LP loop and the third quartet returned to the X-ray size (∼12.5 Å) within 30 ns. The fully base-paired loop again moved as a rigid body, similar to its behaviour in Simulations **1, 2** and **3**. Thus, ion screening is essential for proper positioning of the LP loop, which shows substantial conformational adaptation even without any dynamics of its internal base pairing.

The conformations of the A5 and C9 propeller loops were maintained in Simulation **4**. This is probably because they are very short and the upstream/downstream nucleotides are the firmly fixed G-stem guanines which are stabilized by channel cations. Such structural stability at a short time-scale in absence of the bulk ions obviously does not rule out that the propeller loop ion binding sites still contribute thermodynamically to overall GQ stability. Likewise, the C11T12 loop does not show any interesting dynamics.

### No-salt denaturing simulation (Simulation 6) reveals vertical strand slippage and loss of conformation of single nucleotide propeller loops

**Figure 7.**
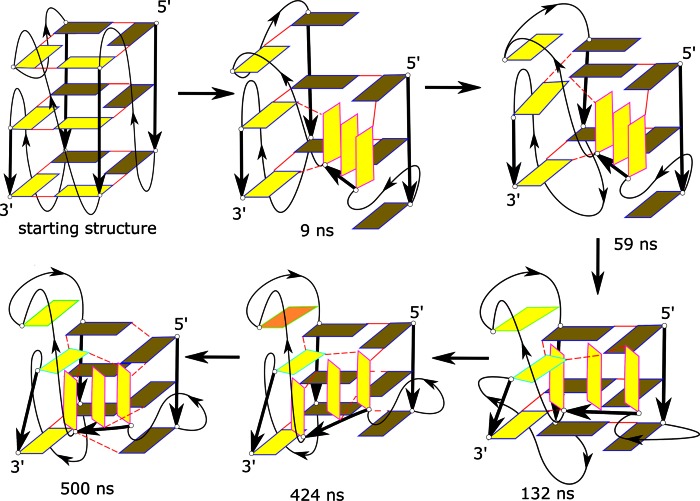
The unfolding pathway of *c-kit* promoter GQ as revealed by the 500 ns long no-salt Simulation **6**. The stem guanines are shown as rectangles, those at the back (strands **a** and **d**) are coloured darker than the ones at the front. The thick black arrows indicate the stem backbone while the loops are indicated as thin black curves. The arrows mark the 5′-3′ direction. The colour of the edges of the rectangles marks residues with approximately the same plane vector. The solid red lines represent characteristic W-C/H base pairing while dash red lines indicate any other hydrogen bonds. A1 is not shown. The time below the structures indicates the time development; the orange base at 424 ns is the *syn* G10. See Supplementary Figure S25 for cartoon representation; the Supplementary Data section also contains the corresponding PDB files.

No-salt simulation is an efficient tool to investigate early stages of GQ unfolding (see Discussion and ref. (38)). In the case of the *c-kit* promoter GQ, the unfolding in the first 500 ns no-salt simulation (Simulation **6**) was initiated by vertical strand slippage of strand **a** at 9 ns (Figure 7, Supplementary Figure S25, Supplementary Movie 1, Supplementary PDB files of key structures). The first and second bases of strand **b** (G2, G3) paired with the second and third bases of strand **d** (G14, G15). Simultaneously, strand **b** misaligned and inserted itself into the collapsing central stem channel. At 15 ns, G10 in strand **c** moved vertically over the first quartet. Residue G21, which is below G10 in strand **c** also moved up and formed a hydrogen bond with the first base of strand **d** (G13). Subsequently, G10 stacked with C11 and T12 of the lateral loop over the GQ (at around 130 ns). G10 became highly flexible and flipped into a *syn* confomation at 424 ns. The G22–G15 interaction was the only native G-stem base pair that lasted until the end of the simulation (500 ns) as it was stabilized by stacking with A16 and G20. Overall, the no-salt simulation showed rapid perturbation of the native arrangement. The initial vertical strand slippage is the most facile rearrangement in all-parallel all-*anti* GQs, which is consistent with the literature data (38). In addition, this simulation indicated that the early movement of both strands **a** (direction of its vertical slippage) and **b** (slippage and insertion into the G-stem channel) was driven by forces acting to abolish the arrangements of the two single-nucleotide propeller loops at ∼9 ns. These propeller loops may represent the structurally most fragile parts of the GQ, at least as described by the force-field used here. After the structures of the propeller loops were lost, further weakness of the structure in the region of the inserted ‘non-tract’ G10 became apparent.

In order to better understand the meaning of the loss of the propeller loops structure we note that their characteristic V-shape may be sometimes lost even upon preservation of the original G2-G6 (or G6-G10) pairing of the native 5′-end quartet. This occurs when the orientation of the G-strands sandwiching the particular loop is significantly altered (Supplementary Figure S26). Obviously, loss of the above noted base-pairing (more specifically, increase of the inter-base distance to more than ∼4 Å) always leads to loss of the characteristic propeller loop V-shaped structure.

### Refolding of the structures observed in no-salt simulation (Simulations 7a–i)

Eight snapshots were taken from the no-salt Simulation **6** (at 9, 59, 78, 132, 160, 255, 348 and 424 ns) and probed by nine standard K^+^ 100–500 ns simulations to attempt a repair of the perturbed structures. In some of the simulations, we observed a partial tendency for refolding, including one event that can be considered to be a spontaneous refolding of a single nucleotide propeller loop. Nonetheless, none of the simulations fully repaired the native structure.

The snapshot taken from 9 ns included vertical slippage of strands **a** and **b**, with strand **b** inclined to the G-stem axis and with loss of the V-shape structure of both propeller loops. In the 300 ns simulation of this snapshot (Simulation **7a**), we did not achieve any significant refolding. The strands **b** and **c** remained misaligned and their native interactions were not formed (Supplementary Figure S27). The snapshots from 59 to 424 ns were even more perturbed, with vertical slippage of the intervening base G10 and the G21 of strand **c**. Two attempts were made to refold the 59 ns snapshot (Simulations **7b** and **7c**). The 250 ns Simulation **7b** resulted in further loss of native base pairs (Supplementary Figure S28a). Similarly, no refolding was observed in Simulation **7c**. The native base pairs between strands **a** and **b** were not formed. Also, G10 and G21 of strand **c** did not move back vertically to form native interactions (Supplementary Figure S28b). However, in the refolding attempts of snapshots at 78 and 132 ns (Simulations **7d** and **7e**) significant native Hoogsteen-like interactions were formed between strands **a** and **b**. The base pairs G3-G7 and G4-G8 were formed in Simulation **7d** (Supplementary Figure S29). In Simulation **7e**, along with these base pairs, a G2-G6 base pair was also formed. We consider this to be the most successful refolding attempt in this series of refolding simulations because the V-shaped conformation of propeller loop A5 was re-established. The corresponding bases in the strands joined by the loop (strands **a** and **b**) became coplanar and achieved their native interactions (Supplementary Figure S30). However, G10 of strand **c** remained extruded from the stem in both these refolding simulations (Supplementary Figures S29 and S30). The G10 could not move back as its movement was obstructed by its stacking interaction on T12.

The simulations of snapshots at 160, 255, 348, 424 ns (Simulations **7f**–**7i**) were carried out for 500 ns. The native interactions between strands **a** and **b** were not formed and G10 of strand **c** could not insert into the stem in any of these simulations (Supplementary Figures S31 and S32). Interestingly, in the refolding attempt of snapshot at 348 ns (Simulation **7h**), C9 became inserted in place of G10 into the terminal quartet and formed a stable misfolded GQ (Supplementary Figure S31c). G10 was already in the *syn* conformation in the structure at 424 ns. In the 500 ns refolding Simulation **7i**, G10 flipped back into an *anti*-orientation at ∼340 ns but showed no sign of re-insertion (Supplementary Figure S32).

In summary, we have observed trends to refold the structure in some of our standard refolding simulations, but none of them was capable of re-inserting G10 back into the GQ once the native G10 position was lost in the starting structure. This would probably require a longer time-scale. Further, the nine refolding simulations gave an impression that formation of propeller loops is difficult on a sub-microsecond time-scale because only one instance of repair of a propeller loop was observed (A5 in Simulation **7e**). Refolding the propeller loop is certainly more difficult than re-establishing the Hoogsteen base pairs. We also emphasize that even this propeller loop refolding event could be affected by a memory effect, i.e. it was not achieved from a fully unfolded structure.

### Second no-salt simulation (Simulations 8 and 9a–g)

**Figure 8.**
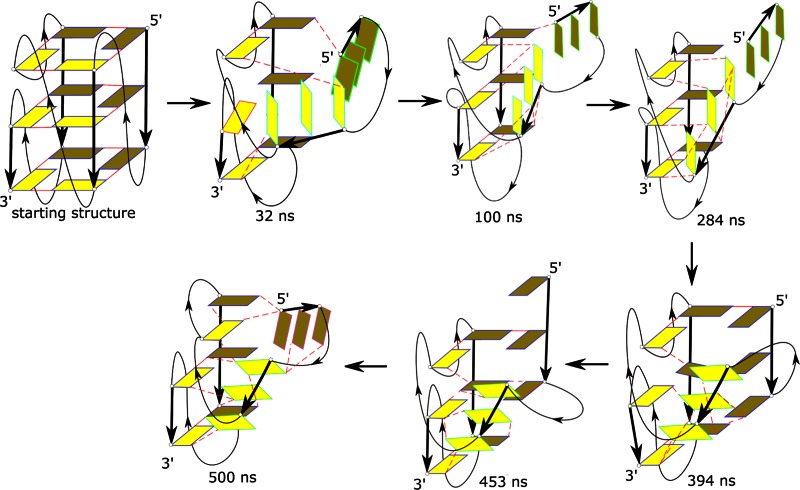
The unfolding pathway of the *c-kit* promoter GQ as revealed by the second no-salt Simulation **8**. See legend to Figure 7 for further details. Unfolding is initiated by the simultaneous loss of conformation of the single residue propeller loops A5 and C9. Although strand **a** is capable of a very rapid return back to the structure, strand **b** remains inclined to the G-stem axis. The strands **a** and **d** are at the back (dark colored) while strands **b** and **c** (light colored) are at the front. See Supplementary Figure S33 for cartoon representation; the Supplementary Data section also contains the corresponding PDB files.

The unfolding in the second no-salt simulation was fast and was again initiated by strand slippage (Figure 8, Supplementary Figure S33). Strand **a** moved dramatically vertically at 32 ns (Figure 8, Supplementary Figure S33, Supplementary Movie 2, Supplementary PDB files of key structures). Strand **b** was also inclined to the G-stem axis with a loss of native base pairs. The conformations of the single residue propeller loops A5 and C9 were simultaneously lost. Strands **c** and **d** retained the native base pairings, stacking with the bases from the LP loop and formed a stable duplex-like structure. At ∼390 ns, strand **a** realigned back along the stem axis (before slipping and again bending away) but the conformations of the single residue propeller loops A5 and C9 remained lost till the end of the simulation as strand **b** remained inclined to the rest of the structure.

We took seven snapshots (at 32, 100, 284, 327, 394, 453 and 479 ns) from the second no-salt simulation (i.e. Simulation **8**) and attempted refolding in standard K^+^ 730 ns –2 μs long simulations (Simulations **9a**–**g**). One of these simulations (the 284 ns snapshot, Simulation **9c**) was capable of moving decisively towards the native GQ structure (Supplementary Figure S36, Supplementary Movie 3). In this simulation the first sign of refolding towards the native structure was observed at ∼220 ns with a loss of stacking interactions between strands **a** and **b**. Following this, strand **a** partially aligned with the G-stem axis (with some residual inclination). At ∼260 ns, strand **a** straightened further and formed base pairs with strand **d** albeit with strand slippage. G3 and G4 of strand **a** paired with G13 and G14 of strand **d**, respectively. At ∼300 ns, strand **a** underwent vertical back-slippage and formed native base pairs with strand **d**. However, G4 remained tilted and formed a stable non-native hydrogen bond with N3 of G15. Strand **b** also realigned and formed native base pairs at ∼400 ns. The V-shaped structure of the C9 propeller loop was refolded. However, due to the persistent G4 mis-interaction, the A5 propeller loop structure was not fully repaired. The structure achieved at 400 ns was then stable (i.e., the G4 position was not repaired) till the end of the simulation which we prolonged to 2 μs. Nevertheless, we consider this simulation as being the most successful refolding attempt in this whole study (see the Supplementary Data section for full documentation) and a spectacular example of atomistic micro-pathways that can participate in the latest stages of folding. It started from an initial phase with no visible movements followed by a ∼200 ns transition period of decisive rearrangements, from the quite perturbed structure towards the native structure which was then again halted by formation of a single non-native interaction due to subtle mis-insertion of G4.

In the remaining six refolding simulations strand **b** always remained inclined to the G-stem axis (Supplementary Figures S34, S35, S37-S40). Its position was locked by a network of non-native hydrogen bonds with bases of strands **c** and **d**. The ion channel collapsed and only one cation was retained inside these structures. It is apparent that the initial structure perturbations were too large to initiate re-folding. The structure with the inclined strand **b** can serve as an example of how variable structures can be that temporarily occur during GQ folding and unfolding pathways at the atomistic level. It further supports the view that GQ folding is, at the atomistic level, a multi-pathway process with a highly uneven (rugged, frustrated) free energy surface (38,51), which is very different from the funnel-like folding of short fast-folding proteins.

### Additional six no-salt simulations (Simulations 10 & 11) confirm the weakness of the single-nucleotide propeller loops

The unfolding of the *c-kit* promoter GQ was initiated by loss of the structure of the single nucleotide C9 propeller loop (the A5 propeller loop unfolded shortly after that) in the first no-salt simulation and simultaneous loss of the A5 and C9 propeller loop structures in the second no-salt simulation. We carried out six additional no-salt simulations using TIP3P and SPC/E water models (Table 1, Supplementary Figures S41, S42). These simulations confirmed that the loss of conformations for the single nucleotide propeller loops A5 and C9 (associated with the predominantly vertical movements of the strands **a** and **b**) is a hallmark of early stages of unfolding of the *c-kit* promoter GQ initiated by the no-salt condition. Further details are given in the Supplementary Data section.

## DISCUSSION

We have used an explicit solvent MD approach to explore conformational behaviour and structural stability of the *c-kit* promoter GQ in differing sets of conditions. Improvements in DNA force-fields (49,72,73) have enabled us to run long simulations. A total of ∼50 μs of simulations are presented here, three of them being expanded to 10 μs (Table 1). We have used the recent DNA-specific χ_OL4_ (49) and ϵζ_OL1_ (73) force-field refinements (not available in the older studies but now fully endorsed in the AMBER code) that were added to the basic bsc0 (72) force-field variant. For the sake of completeness, we carried out a few ∼1 μs scale simulations with the bsc0 correction alone and also this force-field variant appeared to provide stable trajectories (data not shown). Note, however, that especially using χ_OL4_ would be quite essential for longer simulations of GQs with *syn* guanosines.

### The overall structural dynamics reveals a stiff stem and flexible loops

Our standard simulations describe the GQ stem including its internal ions as entirely stable. The 5′-terminal A1 flips from *anti* to *syn*, as expected for a solution environment in the absence of crystal packing effects (45,85). Some plasticity is observed in the four loops. The backbone of single-nucleotide propeller loop A5 was the least dynamic (Figures 3 and 5, Supplementary Figure S9). The C9 propeller loop and C11 in the C11T12 lateral loop showed higher flexibility (Supplementary Figure S9c). In the GQ crystal structure B of 3QXR, the single-nucleotide propeller loop A5 showed more fluctuations than the C9 propeller loop and the C11T12 lateral loop, as revealed by the B-factors (Supplementary Figure S9b). However, the flexibility and base fluctuations in C9 and C11 had also been observed in recent shorter simulations of this GQ (32). Simulation **2** even resulted in a permanent flip of C11 with formation of base-backbone interaction (Figure 5D & Supplementary Figure S14d). This substate is not seen in the experimental structures. T12 of the lateral loop was often stacked with G13 of the first quartet in our simulations. Most interesting has been the behaviour of the internally structured long LP loop. It shows distinct positions in the NMR and crystal structures (30–32). Our long standard simulations readily sampled both experimentally-observed conformations of the LP loop (and the space between them) (Figures 3 and 5, Supplementary Figure S43). The LP loop A16–G20 and the G17–A19 base pairs are identical in the experimental structures and are stable in all simulations (Supplementary Figures S12, S14 and S17). These interactions stabilize the bases in the LP loop while the backbone is flexible in both the crystal structure and the simulations (Supplementary Figures S9b, S9c). The entire LP loop fluctuates as a rigid segment while firmly maintaining its internal structure and salient intra-loop base pairing (Supplementary Figure S43).

In summary, our results demonstrate that long simulations are needed to appropriately sample the loops. Even the simple A1 *anti* to *syn* rearrangement required several μs to occur (Supplementary Figure S12a). Shorter simulations could easily lead to entirely incorrect interpretations and this should be taken into account when considering older literature data. We are obviously far from claiming that our 10 μs time scale is already converged. In fact, as discussed above, there are several aspects of our simulations that we could not ultimately resolve due to insufficient sampling. We plan in the near future to use enhanced sampling methods (71) to increase sampling of the *c-kit* promoter GQ loops though these methods also have their own limitations and cannot fully replace standard simulations (75).

### External cations may stabilize the structure of the *c-kit* promoter GQ

Our simulations indicate that, besides the channel ions, the *c-kit* promoter GQ can be partially stabilized by monovalent ions interacting with the loops (Figure 6 and Supplementary Figure S20, Table 2 and Supplementary Table S2). Monovalent cation binding site with high occupancy (>50%) formed in all simulations between the G21 and the C9 phosphate and may be related to a similar ion binding site seen in the crystal structure, where structured cations and the water network coordinate with C9 and G21 residues (Figure 1). Another highly-occupied site was observed at the interface of the C11, G10 and G21 sugar phosphate backbone (Figure 6D and Supplementary Figure S20d). The cation at this position may shield backbone electrostatic repulsion and thus support the structure of the lateral loop. This ion binding site has been observed in both GQs A and B of the 3QXR crystal structure (Figure 1) (31).

The very recent *c-kit* promoter GQ structure (PDB id: 4WO2) does not show specific ions in coordination with any of the loops, most likely as a consequence of the lower resolution of this structure compared to the earlier one. It is also possible that some ion binding sites may be obscured by the known difficulty of X-ray crystallography in unambiguously identifying more mobile nucleic acid monovalent ion binding sites (87,88,92,101). In considering the overall picture from the present simulations, taken together with the experimental data and earlier simulations of highly occupied ion binding sites, we suggest that the interactions with the ions can contribute to the stability of the GQ. However, none of the ion binding sites appears to be indispensable. Even the most developed *c-kit* promoter GQ loop ion binding sites show relatively low occupancies compared to major ion binding sites in many folded RNA molecules that have essentially 100% ion occupancies in MD simulations (88,91,92). This view is further supported by our 1.25 μs simulation in the absence of any external ions (limited salt Simulation **4**) which shows a stable structure (Supplementary Figure S24). We performed two additional limited-salt simulations of 500 ns with only one channel cation (i.e., only one ion in the whole simulation box, data not shown). In these conditions as well the GQ including its loop base pairing was stable. The only exception is the long LP loop. Although it is internally stiff and inflexible, it expanded (moving as a rigid body) in all these limited-salt simulations, forming a large cleft between this loop and G-stem.

Considering all the data we suggest that binding of cations to the exterior of the *c-kit* promoter GQ provides auxiliary stabilization to the loop conformations but is not decisive for retaining the integrity of the GQ structure. Ion screening is, however, important for positioning of the LP loop. We note that the potential role of specific ion binding to loops (and their potential role in GQ stabilization) has also been suggested by other groups (95,102). Unfortunately, it appears difficult (for both theory and experiment) to quantify the net free energy effect of loop ion binding, i.e. to uncouple the free energy effect of ion binding from the other contributions.

### The single nucleotide propeller loops are the most fragile structural components of the *c-kit* promoter GQ and their formation may represent a constriction on its folding landscapes

The topology of the *c-kit* promoter GQ is consistent with the findings that single-nucleotide loops typically adopt propeller arrangement and thus result in parallel orientation of the strands involved (103–105). In general, single-nucleotide loops are common in GQs throughout the human genome (106) and single-nucleotide propeller loops have been observed in several atomistic structures (7,107–112). Previous experiments have shown that melting temperatures of GQs decrease as the loop lengths increase and longer loops have destabilizing effect on the GQ (104,105,113–115). The experiments carried out on model sequences predict that single nucleotide loops are the most stable ones (113,116,117). The stability of the single nucleotide loop formed by adenine has been predicted to be reduced compared to cytosine (104). However, in a study on naturally-occurring GQ sequences found in promoter regions of various proto-oncogenes, no simple correlation between the loop length and thermodynamic stability of GQs could be derived (118). This study reveals that along with the loop length, the loop composition and loop symmetry also influence thermodynamic stability of the GQ. A previous computational free-energy study (atomistic MD and MM-PBSA method) has predicted that short loops are less favourable than long loops (46). In summary, the current picture of the effect of loops on stability of GQs is far from being conclusive. Despite this, it is evident that the loops and flanking nucleotides greatly modulate the primary GQ topology preferences pre-determined by the number of guanines in the G-strands (45,85,119). It should be noted that most of the experiments are not based on atomistic methods and would thus have difficulty in identifying eventual inclusion of the guanines into the loop regions or other unexpected structural features, which could bias the results. For example, the UV-visible and CD spectroscopy signals are similar for different antiparallel GQs (120,121). The signals in these methods are also insensitive to the number of propeller loops and their composition in parallel-stranded GQs (121). Obviously, the presence of a mixture of structures (or presence of an unfolded fraction) may entirely bias structural interpretation of the experiments. For more general discussion of the limitations of spectroscopic methods in determining GQ structures see (122,123). Nevertheless, despite some uncertainties, experimental data does show that single nucleotide propeller loops are common and stable.

Several recent MD studies have investigated possible intermediates in the folding of human telomeric GQs, which can have three-nucleotide propeller loops (38,51,61). These studies noted that formation of the propeller loops is a non-trivial problem, since the simulations revealed a profound tendency of the propeller loops to be straightened and disrupted. These studies thus concluded that formation of propeller loop structures of human telomeric GQs is much less straightforward compared to lateral and diagonal loops.

The present study for the first time probes the stability of single nucleotide propeller loops by simulations. These loops are entirely stable in standard simulations of the complete GQ. This is consistent with the known extraordinary structural stability (compared to affordable simulation timescales) of the ion-stabilized GQ stems. However, denaturing no-salt simulations (Figures 7 and 8 and Supplementary Figures S41, S42) lead to spectacularly speedy loss of the propeller loops. Typically, the GQ structure is disrupted through ejection or rotation of strand **b** sandwiched by two such loops. The most plausible explanation of this result is that the sharp bend of the propeller loops is highly unstable *per se*. The single-nucleotide propeller loops appear to be under some considerable energy strain and are experiencing immediate unfolding once they are deprived of the full support of the ion-stabilized G-stem. In other words, the simulations predict that the single-nucleotide propeller loops represent the most fragile part of the *c-kit* promoter GQ, being even more strained than the three-nucleotide propeller loops of the human telomeric GQ. Thus, we suggest that formation of the propeller loops represents one of the most peculiar atomistic aspects of the GQ folding pathways. However, it is presently not clear to what extent this behaviour reflects real properties of such loops and to what extent it may be affected by some hitherto unidentified force-field imbalance. The single-nucleotide propeller loops shift the backbone of both preceding and succeeding guanines into the non-canonical regions. The X-ray structures and the present simulations show that in the 5′-G_i-1_N_i_G_i+1–_3′ segment, when the propeller loop is formed, G_-1_ is in a B_II_ ϵζ backbone conformation and the α angle of G_+1_ is in a non-canonical region. The available experimental structures do not allow us to more precisely specify this backbone conformation, as it varies across the structures (31,32). Nevertheless, it is possible that the force-field either excessively penalizes the shift of the backbone to the non-canonical region or does have some problem in accurately treating some clustering of the anionic phosphates around the propeller loop. If the potential imbalance is related to electronic structure redistributions, it would be obviously not reparable by minor force-field refinements modulating the torsional potentials of the DNA backbone that are common in the contemporary literature. Resolving this issue will require further studies including comparison of the force-field with benchmark quantum-chemical calculations.

It is important to point out that we have been able to restore the propeller loop V-shaped architecture in a small fraction of our refolding simulations, namely in Simulations **7e** and **9c** (Supplementary Figures S30, S36, Supplementary Movie 3). However, the significance of these events should not be overemphasized, since the simulated structures could still have some structural memory from the native structure after the preceding short denaturing simulations. In other words, the structures most likely did not fully reach the true unfolded ensemble in the preceding no-salt simulation. Furthermore, the propeller loop refolding occurred upon completing the native ion-stabilized quartets, which is a situation corresponding to the very end of folding. It is more difficult to imagine spontaneous formation of the propeller loops during earlier stages of the GQ folding (for example, in case of forming G-hairpin structures or G-triplexes) (51). We have actually noticed an irreversible loss of conformation of a propeller loop in MD simulations of ion-stabilized G-triplexes, which is in contrast with the stable behaviour of lateral and diagonal loops (51).

It is well established that fast folding (millisecond-scale) proteins fold via a funnel-like single-pathway mechanism and that their sequences are optimized to have a smooth free-energy folding landscape (124,125). Because folding of GQs is much longer (often hours) (120) it is likely that the GQ folding surface is very rugged, with multiple competing and deep non-native free energy basins of attraction (most likely alternatively folded GQs with diverse *anti*/*syn* distributions), resulting in a long multi-pathway process (51). This picture is consistent with our current, as well as preceding and ongoing simulations. The simulations suggest that formation of the propeller loops may lead to some constrictions on the folding landscapes of the GQs. This is highlighted in the present work as out of sixteen refolding attempts described above, significant refolding was observed in only two simulations (Simulations **7e** and **9c**). We have carried out four more independent refolding attempts of starting structure in Simulation **9c**, using random seeds, which are not presented in the Results section. However, none of these 1 μs long simulations showed the necessary realignment of strand **b** to structure the propeller loops (data not shown). This is due to the stochastic nature of simulations. In other words, our successful Simulation **9c** required some chance with random sampling. It indicates that, in principle, the V-shaped propeller loops may be formed by fast but very rare transitions across the transition state ensembles which would be difficult to capture on the present time scale of atomistic simulations (126,127). Such a scenario could best reconcile the above-described simulation behaviour of propeller loops and their evident common occurrence in known structures. Note that folding and unfolding pathways may differ, and the folding events may utilize sudden but rare rearrangements of a given strand, for example to pair with a triplex structure stabilized by ions (120,128–130). Actually, Simulations **7e** and **9c** can be considered to be the first atomistic examples of such folding attempts visualised to date, albeit we reiterate that their starting structures most likely did not reach the true unfolded ensemble (for example, all structures kept their native *anti*/*syn* distributions and some other attributes of the native GQ architecture). Still, as noted above, we cannot ignore the possibility of a somewhat unbalanced force-field description of the propeller loops, resulting in their under-stabilization in simulations. This would complicate future GQ folding studies using MD simulation techniques.

### The LP loop is very stable and may dictate the folding topology

The five nucleotide LP loop is exceptionally internally stable under all simulation conditions, including the denaturing simulations. A comparison of the thermal mobility of the atoms in quadruplex B of crystal structure 3QXR and RMSF of atoms in the simulations also highlights that the backbone of LP loop is flexible while the bases are fairly rigid (Supplementary Figures S9b, S9c, S43). In the crystal structure, backbone atoms of A16, G17 and G18 residues of the LP loop show fluctuations while in the simulations A16 is stable but the backbone atoms of G17, G18 and A19 show significant fluctuations (Supplementary Figures S9b, S9c). It is noteworthy that the backbone torsion angles of A19 show large differences between the NMR and crystal structures (31). Therefore, the flexibility of A19 in the simulations is not surprising. We suggest that the extraordinary internal stability of this loop is directly contributing to the overall unique topology of the *c-kit* promoter GQ. The stability gain by forming its internal network of GA base pairs and stacks may be dictating which of the many possible GQ topologies is encoded by this unique promoter sequence. This may be also the reason why the sequence selects an arrangement with a discontinuous strand **c**. The no-salt simulations indicate that the inserted G10 nucleotide may be another structurally weaker point of the structure, albeit still much more stable than the propeller loops. The crystal structure revealed that water molecules form extensive networks throughout the GQ structure (31). In the simulations, a spine of hydration was also formed along the entire length of the GQ and linked the bases of the LP loop to the G-stem bases (Supplementary Figure S44a). The hydrogen bond donors and acceptors of the firmly structured LP loop formed a scaffold for water binding (Supplementary Figure S44b). The bound water molecules and the base interactions form a framework within the LP loop such that it behaves as a rigid body (Supplementary Figure S43). In our unfolding simulations, the LP loop bases even stabilized the quartet stem by forming stacking interactions with the guanine bases of the third quartet. This stabilizing effect was evident in some of our refolding simulations, since the stacking of the LP loop bases with the third quartet bases formed a scaffold on which the second and first quartet became re-established (Simulations **7a-i**).

### Additional comments on refolding simulations

We have attempted altogether twenty re-folding simulations using diverse starting structures taken along the path of the denaturing no-salt simulations. Some of the simulations showed a visible trend towards refolding, though none of them achieved complete refolding. A hallmark of the difficulties in refolding was the restoration of the structures of the V-shaped propeller loops which has been discussed in detail above. Further, the simulations were unable to spontaneously re-insert the G10 back into the stem once it was ejected. In excess salt simulations of these structures, the cation in between the first and second quartet readily escaped into the bulk due to incomplete coordination. The re-insertion of G10 was hindered as it formed stacking interactions with T12 of the lateral loop. We thus carried out a simulation (not noted in the Results section) with distance restraints between G10 and T12 forcing these two bases to separate. In this heavily biased simulation, G10 re-inserted back into the first quartet a few ns after the T12 base had been shifted away and the native GQ was formed (data not shown). This indicates that a spontaneous re-insertion of G10 would be achievable on a longer time scale with T12 thermal fluctuations.

Many of the re-folding simulations ended up in a stable misfolded arrangement, with the **b** stem perpendicular and extensively hydrogen-bonded to the rest of the structure. We suggest that this arrangement (Figure 8, Supplementary Figures S34-S40) is an example of one of the very many individual atomistic structures that can be populated in the course of the natural (un)folding processes.

The cations may also mediate the movement of the guanine bases and facilitate their alignment with the G-stem. Such cation-mediated movement of base into the stem was observed in re-folding Simulation **7e**. G21 is a snapback base and its insertion into the G-stem could be hindered by the phosphate-phosphate repulsion with C9. The cation bridging between C9 and G21 backbone shielded the repulsion, thereby facilitating insertion of G21 in the stem.

In summary, although our simulation time-scale was not sufficient to completely reverse the early stages of unfolding initiated by the denaturing no-salt simulations, a propensity was apparent for the perturbed structures to move back to the native structure after adding the monovalent salt.

### Force-field approximation of ion description in MD simulations of GQs

MD aims to characterize selected aspects of the structural dynamics and stability of DNA molecules. In common with every scientific tool, MD has specific limitations which need to be considered in order to execute the simulations correctly and to interpret the results appropriately. When the genuine advantages and limitations of the method are not properly taken into consideration, it results either in serious over-interpretation of the MD results or groundless criticism of the method. Our recent reviews provide a critical assessment of the methodology and explain its relationship to different types of experiments (41,75). A unique aspect of MD simulation of GQs is the inclusion of channel cations. This is because of the role of the cations in the GQ structures and because of the simple form of the force-field, which somewhat compromises the description of the ion binding, mainly due to the lack of explicit polarization (131).

Neither experiment nor computations allow direct decomposition of overall free energies into individual terms (132,133). Various factors including ion binding, hydration pattern and base stacking contribute to GQ folding and stability (38,120,134–136). Nevertheless, it has been suggested that during GQ folding a rather large electrostatic energy is compensated by other forces (105,136,137). These are likely to arise from ion condensation and ion binding. In the absence of cations, the fraction of folded states is very small. As specific ion binding to the unfolded state is likely to be small, the stabilization due to specific ion binding should be substantial and overcomes unfavorable electrostatic interactions due to folding (105). Thus, it appears that GQ folding is an energetically unfavorable process *per se* and is driven by energy derived by cation binding with little or no contribution from other weak molecular interactions (138). It nevertheless does not exclude the possibility that some GQ folding sequences may be correctly prefolded in the absence of the ions, as suggested by NMR experiments for the dimeric *Oxytricha nova* telomeric GQ (139). MD simulations are consistent with this picture and indicate that ion binding inside the GQ stems is the dominant stabilization energy term. In the presence of the channel ions (and using a correct force-field), the GQs are exceptionally structurally stable and stiff, as confirmed by the present simulations (the longest published to date). By stiffness we refer to the structural dynamics (thermal fluctuations) of folded GQ stems on the timescale probed by the simulations. In contrast, in the absence of the channel ions, the GQ stem structure is immediately destabilized (even though the channel is hydrated) and unfolds, as visualized by our no-salt simulations. When simulating initially ‘vacant’ GQs in the presence of bulk ions, the stems have a high capability to retrieve ions from the bulk, which leads to their immediate stabilization (43,140,141). Finally, our present re-folding simulations show that addition of cations to perturbed structures initiates re-formation of the G-quartets, although this has not been achieved in all simulations due to the limited simulation time scale.

Despite the force-field limitations, the MD technique robustly captures the basic stabilization of the GQs by the monovalent ions. It is because the stabilization primarily originates from long-range electrostatics correctly described by the force-field and also because the GQ stems are very stiff molecules with deep free energy basins, making the simulation behaviour insensitive to minor force-field deficiencies (41,42). It is, however, useful to keep in mind that the force-field description of the ion binding is only approximate. The simulations are not carried out with ‘real’ ions, but within a simple physical model (termed a pair-additive approximation) using van der Waals spheres with atom-centered +1 point charges. This leads to at least three deficiencies primarily caused by lack of geometry-dependent polarization energy, which cannot be eliminated when using the simple force-field form. All of these deficiencies have been rigorously assessed by quantum chemical calculations. The first one is quite straightforward; it is an underestimation of the direct quartet-ion interactions due to lack of polarization (41,42,50). The second one is a more complex electronic structure phenomenon, which is manifest as an overestimation of the apparent inter-cation repulsion between multiple ions inside the G-stem by the force field. The real ions are partly re-distributing their +1 charges into the molecular orbitals of the G-quartets (in a geometry-dependent manner) and polarizing the guanines (131). The final effect on the energies is as if the cations would have a variable charge smaller than +1 depending on their exact position within the GQ structure. This reduces the ion-ion electrostatic repulsion between the real ions compared to the force field ions in a highly geometry-dependent manner. For these reasons, the constant point-charge approximation of the force field then necessarily breaks down to a certain extent for closely-spaced ions inside the G-stems. Elimination of this error would require geometry-dependent force-field parameters of the overall system, due to many-body polarization interplay between the ions and the quartets (131). The third problem is an overestimation of the apparent size of the ions with respect to the GQ channel size, since it is not possible to simultaneously optimize the ion parameters for their hydration energies and their G-quartet binding (41,42,50). The description of the GQ stems thus would profit from making the force-field ions smaller, however, this would compromise their hydration energies and would still not resolve the first two deficiencies (50). Thus, when we use Na^+^ ions in the present GQ simulations, the apparent size of the cation inside the GQ is somewhere between ‘real’ Na^+^ and K^+^ ion sizes (50). Use of K^+^ parameters would not necessarily bring the simulation closer to the real K^+^ environment. The cumulative effect of all three of these inaccuracies may lead to occasional spurious (usually temporary and very rare) expulsions of the K^+^ ions from the GQ stems (50). Although no such K^+^ ion fluctuations were noticed in the present study, they have been documented elsewhere (50). Such expulsions are not consistent with the experimentally suggested time-scale of ion exchange between the bulk and GQ stems and the fact that K^+^ exchange is slower than Na^+^ exchange (142–144). Instability of cations inside GQ stems is a serious problem in simulations in some other DNA force-fields (including CHARMM) and a profound instability of G-stem cations always means that a non-optimal force-field has been applied (50).

A common misunderstanding is a requirement that the GQ simulations must be done using the same ions (either Na^+^ or K^+^) as used in the experiments. This requirement would be justified only in computational studies which calculate relative stabilities of different GQ forms and traverse between different GQ conformations. The fact that (for example for the human telomeric GQ) Na^+^ and K^+^ may stabilize different topologies does not mean that the unobserved topologies are entirely non-existent. In fact the history of experimental studies on human telomeric GQs shows that new topologies are frequently being found, and are often dependent on the precise nature of the flanking sequences. Ion replacement changes the relative free energies (and thus populations) of different GQ forms, making some of them not detectable for a given sequence. However, simulations starting from any known folded structure are valid irrespective of the ion type used, since they are too short to experience any unfolding. This is supported by a recent NMR study which shows that the Na^+^/K^+^ replacement in Tel23 GQ does not lead to any changes of the GQ topology on time scales that are orders of magnitude longer than the MD time scales (122). Specifically, real-time hybridization experiments for the Tel23 GQ using the complementary WC strand reveal an unfolding time constant ∼17 minutes for the Na^+^ form of the Tel23 while the Na^+^/K^+^ ion exchange inside the structure is much faster (122). This unfolding time scale is consistent with other previous studies (145). This experimental data further supports the claim that Na^+^ and K^+^ MD simulations are equally valid for all known GQ folds irrespective of the existence of the eventual ion-dependence of the thermodynamic equilibrium of different GQ folds. The simulations are ∼7 orders of magnitude shorter than the unfolding time constants suggested by the real-time hybridization experiments for Tel23. Because different GQ topologies are separated by large energy barriers, their simulations always remain within the conformational basins of the starting structures and do not sense eventual shifts of relative free energies of different GQ topologies caused by the ions (38). In addition, the *c-kit* promoter GQ appears to adopt a unique topology and does not seem to be sensitive to the nature of the ions.

The above arguments explain why we (and some other groups) often prefer to make MD simulations on a GQ in the presence of Na^+^. The above explanations also justify the use of no-salt simulations to probe the early stages of GQ unfolding. The basic concept (no-salt simulation followed by standard simulation) resembles reversed stop-flow experiments. The folded GQ molecule is first exposed to denaturing chemical (ion-free) conditions and after some perturbation is achieved, repair of the structure is attempted. This is an efficient tool to obtain atomistic insights into the early stages of unfolding and the potentially late stages of folding of GQs (38). One of the reasons for this is that GQ structural transitions are likely occurring as sudden rare events during time periods where the GQ has a temporarily reduced number of ions in the channel due to ion exchange with the bulk solvent. Then the central G-quartet core is temporarily destabilised and has an increased likelihood of reaching the transition state ensemble. Thus, no-salt simulations may be more realistic than extremely high-temperature unfolding simulations. Obviously, the subsequent re-folding attempts are not always successful, since when the denaturing simulation achieves too large a perturbation, the refolding may be already beyond the simulation time scale; i.e. the emphasis is on the unfolding part. Any successful re-folding (as observed in some of our trajectories) can be considered to be a bonus. Recently, the first real-time spectroscopy study appeared which may significantly increase the accuracy of characterization of the most stable intermediates (146). This study also pointed out that MD simulations may represent an important tool to study those parts of folding processes that are below the time-resolution of the experiments (146).

We finally point out that the primary goal of the MD simulation method, as reflected by its name, is to provide a direct atomistic picture of structural dynamics of single molecules. MD has essentially an unlimited bottom boundary of time resolution (the upper boundary is governed by the affordable simulation time scale). In that sense the technique is entirely unique and can fill many gaps in the resolution of various experimental techniques, improve their interpretations and prevent formulation of too naive atomistic models based on non-atomistic experimental data. The MD method, in principle, also allows estimation of free energies, either directly from relative populations of the different structures, or via a rich spectrum of specialised free energy methods. However, it is the case that the accuracy of calculated free energies is often questionable and the published free energy data are sometimes too ambitious and over-interpreted. Considering all the approximations affecting the free energy computations (some but not all have been noted above), we consider that the technique is still not sufficiently mature to be able to reliably explain the experimentally known effects of the ion type on stability of different human telomeric GQ folds, which have been well documented by solution ensemble experiments (57,95,102,122,147,148). This would require achieving an accuracy of a few kcal/mol, which for this very complex problem is unrealistic. MD-based free energy computations of even much simpler tasks are also affected by sizable uncertainties (85,149,150). Nevertheless, the cation-dependence limitation is not a concern in the present study, as the basic fold of the *c-kit* promoter GQ is independent of the ion type and consistent results were obtained with both Na^+^ and K^+^.

## CONCLUSIONS

We have performed a series of extended atomistic simulations on the *c-kit* promoter GQ, including three 10 μs-long benchmark simulations, no-salt denaturing simulations and re-folding simulations. The GQ stem is perfectly stable, with all ions firmly bound inside. Also the structural features and conformation of the loops are highly stable. This indicates very good performance of the force-fields.

The results show that very long simulations are needed to capture dynamics of the loops, since several of the reported features required the full 10-μs time scale. However, the dynamics of the lateral C11T12 loop did not converge even on this simulation time scale. One of the simulations resulted in a permanent substate with flipped C11 interacting with the backbone of G22. This does not correspond to any experimental structure and thus might even indicate a force-field imbalance, although a similar geometry has been observed for the adjacent T12 base in one of the X-ray structures. In any case, converged conformational dynamics of this loop is beyond the 10 μs time scale.

The unfolding and re-folding simulations are consistent with our expectations that folding of GQs is a complex multi-pathway process. The simulations suggest that the single-nucleotide propeller loops are the most fragile structural parts of this GQ with a high propensity to unfold (straighten). This indicates, and is consistent with simulation studies on human telomeric GQs, that formation of the propeller loops is one of the most peculiar atomistic aspects of GQ folding. The simulation and experimental data can be reconciled by assuming that propeller loops are formed by fast but very rare transitions across the transition state ensembles which would be difficult to capture on the present simulation time scale. However, we also need to consider the possibility that the intricate topology of propeller loops is not fully satisfactorily described by the force-fields. Further studies are needed to clarify this issue.

The properties of the *c-kit* promoter GQ, as captured by the simulations, indicate that it has many of the attributes needed for it to be a potential target for drug design, especially in GIST and other cancers that involve over-expression of *c-kit*. The single nucleotide propeller loops of the *c-kit* promoter GQ are stabilized by the binding of cations even though the GQ is able to at least temporarily retain its conformation in the presence of just the channel cations. Small molecules with cationic substituents can be assumed to behave rather like cations in the present simulations and can provide additional stability to the structure of the *c-kit* promoter GQ by binding in the non-channel cation sites detailed here and in the crystal structure. The majority of GQ interacting compounds reported to date have been designed to bind and stack with the terminal quartets of a GQ. However, as the LP loop of the *c-kit* promoter GQ is very stable and clearly structured, the cleft between the LP loop and the third quartet of the stem may act as a unique site for small molecule binding and is of a size sufficient to be systematically explored by *in-silico* search techniques. The LP loop itself is internally very stable, but is capable of adjusting its position with respect to the stem. This creates a size-adaptable cleft which can facilitate ligand binding, similar to the shape adaptability of DNA grooves. The flexibility in cleft dimensions is also suggested by comparison of the X-ray and NMR structures. Further, the stability gain by forming the internal network of GA base pairs and stacks of the LP loop may be dictating which of the many possible GQ topologies is realized by this unique promoter sequence. It may suppress the stable existence of competitive alternative folds.

## Supplementary Material

SUPPLEMENTARY DATA
