# Supplementary material for: Extended molecular dynamics of a c-kit promoter quadruplex
Source: Nucleic Acids Res. 2015 Oct 10;43(18):8673–93. doi: 10.1093/nar/gkv785 (PMC4605300; doi:10.1093/nar/gkv785)
Supplement: SUPPLEMENTARY DATA [file supp_43_18_8673__index.html]

Extended molecular dynamics of a c-kit promoter quadruplex — Extended molecular dynamics of a c-kit promoter quadruplex — SUPPLEMENTARY DATA 

# Extended molecular dynamics of a *c-kit* promoter quadruplex

## SUPPLEMENTARY DATA

- SUPPLEMENTARY DATA
- SUPPLEMENTARY DATA
- SUPPLEMENTARY DATA
- SUPPLEMENTARY DATA
- SUPPLEMENTARY DATA
- SUPPLEMENTARY DATA
- SUPPLEMENTARY DATA
- SUPPLEMENTARY DATA
- SUPPLEMENTARY DATA
- SUPPLEMENTARY DATA
- SUPPLEMENTARY DATA
