# Supplementary material for: Extended molecular dynamics of a c-kit promoter quadruplex
Source: Nucleic Acids Res. 2015 Oct 10;43(18):8673–93. doi: 10.1093/nar/gkv785 (PMC4605300; doi:10.1093/nar/gkv785)
Supplement: SUPPLEMENTARY DATA [file supp_gkv785_nar-00989-f-2015-File010.pdf]

Extended molecular dynamics of a *c-kit* promoter quadruplex

Barira Islam[1], Petr Stadlbauer[2], Miroslav Krepl[2], Jaroslav Koca[1, 3], Stephen Neidle[4], Shozeb Haider[4], Jiri Sponer[1,2]

[1]Central European Institute of Technology (CEITEC), Masaryk University, Campus Bohunice, Kamenice 5, 625 00 Brno, Czech Republic

[2]Institute of Biophysics, Academy of Sciences of the Czech Republic, Kralovopolska 135, 612 65, Brno, Czech Republic

[3]National Center for Biomolecular Research, Faculty of Science, Masaryk University, Campus Bohunice, Kamenice 5, 625 00 Brno, Czech Republic

[4]UCL School of Pharmacy, 29-39 Brunswick Square, London WC1N 1AX UK

Supplementary Data section

Equilibration methods

For equilibration, the system was first minimized with 500 steps of steepest descent followed by 500 steps of conjugate gradient minimization with 25 kcal mol^{-1} A^{-2} position restraints on DNA atoms. It was then heated from 0 to 300 K for 100 ps with constant volume and position restraints of 25 kcal mol^{-1} A^{-2}. Minimization with 5 kcal mol^{-1} A^{-2} restraints followed, using 500 steps of steepest descent method and 500 steps of conjugate gradient. The restraints of 5 kcal mol^{-1} A^{-2} were maintained on DNA atoms and the system was equilibrated for 50 ps at constant temperature of 300 K and pressure of 1 atm. An analogous series of alternating minimizations and equilibrations followed using decreasing position restraints of 4, 3, 2 and 1 kcal mol^{-1} A^{-2} consecutively. The final equilibration was carried out with position restraints of 0.5 kcal mol^{-1} A^{-2} and starting velocities from the previous equilibration, followed by a short free molecular dynamics simulation of 50 ps. Temperature and pressure coupling during equilibration was set to 0.2 and coupling during the last molecular dynamics phase was set to 5.

Clustering methods:

MMTSB tool kit (http://mmtsb.scripps.edu/software/mmtsbToolSet.html) was used to cluster the 10 µs long bsc0χ_{OL4} trajectory (Simulation **1**) (Supplementary reference 1). Our aim of clustering was to sieve major distinct conformations sampled in the trajectory. For classification, root mean squared deviation (RMSD) of the whole GQ was used as the parameter and pairwise distances measured as coordinate between the structures were defined by a cut-off reflecting range of conformations and their relative populations. The algorithm generated centroids describing each cluster and gave an RMSD for each cluster with respect to each identified cluster. We used trajectory file of Simulation **1** with only GQ atoms (without ions and water) and frames were

extracted at a time interval of 200 ps yielding 50,000 frames. An RMSD cut-off of 2.4 Å was used for the clustering of 10 µs long trajectory of Simulation **1**.

MM-PBSA methods:

The snapshots for MM-PBSA analysis were taken from 7.450-7.550 µs of the trajectory of Simulation **1** with a time step of 100 ps using the MM-PBSA perl script distributed with AMBER12. The two channel bound Na^+ were included explicitly in the free energy calculations as they contribute to the stability of the GQ stem (Main text reference 41). The Tan and Luo estimated radius of Na^+ was modified in the script to obtain consistent solvation energies (Supplementary reference 2). The solute entropies were not included in the analysis as entropy was assumed to be rather constant in the trajectory portion used for the MM-PBSA calculations.

Supplementary Results

Backbone dihedrals

The G-stems are the most common non-canonical structures used to evaluate the force-fields as they remain largely stable during the simulations. To assess the representation of *c-kit* promoter GQ with the bsc0χ_{OL4} force-field, we compared backbone dihedral angles in Simulation **1** to those observed in the experimental structures (Main text references 30-32). The stem dihedrals are very well represented during the entire 10 µs of Simulation **1** (Supplementary Figures S1-S3). In the first quartet, G6 and G10 sample the backbone angle α in non-canonical regions similar to that observed in the X-ray structure. This can be attributed to the fact that preceding to these bases are the A5 and C9 single nucleotide propeller loops, respectively (see the main text for further discussion). The sugar puckers of G6, G10 and G13 are also flexible throughout the simulation. In second quartet, G3, G7 and G14 show some flexibility in ε/ζ dihedrals. The dihedrals of the third quartet show better agreement with the experimentally observed values. The G4 and G8 are in B_{II} confirmation similar to experimental structures as the backbone here turns to form single nucleotide propeller loops A5 and C9, respectively (discussed in detail in the main text).

The terminal base A1 exhibits both *syn* and *anti*-conformation in the simulation. The ε/ζ dihedrals also changes from B_{II} to B_I conformation when the sugar pucker changes from *anti* to *syn* conformation. This is in agreement with experimental observations where in the crystal structure, A1 is in *anti*-orientation and B_{II} conformation while in NMR it is in *syn*-orientation and shows B_I conformation (Main text references 30, 31). The single residue propeller loops A5 and C9 show high flexibility in all backbone dihedrals. The backbone of lateral loop formed by residues C11 and T12 is also very flexible. The force-field is unable to represent the non-canonical α/γ values in both the single residue propeller loops and the lateral loop. The LP loop is stabilized by the stacking and hydrogen-bonding interactions within the loop. The backbone of this loop shows limited flexibility in the simulation and the dihedrals are represented well with the force-field. The residue A19 of LP loop shows flexibility in sugar pucker as well as in ε/ζ dihedrals. In G20 base as well the force-field is unable to represent non-canonical α/γ values

and instead shifts to canonical α(-)/γ(+) region. We draw the inference that the current force-field reasonably represents the G-stem but the loop residues may be represented less accurately. The largest discrepancies are observed with loop nucleotides which are the most flexible in the simulation. Therefore, while the present simulation is satisfactory as the many of the loop bases have limited flexibility due to interactions within the loop, the results may vary for other GQ systems.

It should be noted that the nucleic acids simulations force-fields should always be considered as only approximate, since their simple functional form (lacking any electron structure redistribution effects, relying on severe approximation of constant point charges, etc.) does not allow a perfect description of nucleic acids. Taking into consideration the overall simulation behavior as monitored in our study, we consider the force-field performance as very satisfactory and sufficient to derive the basic conclusions of our study. From basic physical principles, simple force-fields will never provide a fully perfect description of nucleic acid. For frank assessment of force-field performance see refs. 41 and 75 in the main text, and references therein. To understand the true magnitude of force-field limitations, see ref. 82 in the main text.

Clustering analysis

The simulation **1** trajectory was clustered using an RMSD cut-off of 2.4 Å and nine clusters were obtained (Supplementary Figures S10 and S11). Our aim of clustering was to sieve major conformations populated within the trajectory. The percentage of occurrence of each cluster is presented in Supplementary Table 1. The starting red cluster showed the smallest population in the simulation. Other than the initial few frames this cluster was also present at ~2.2 and 7.5 μs. The 0.7 - 3.5 μs of simulation were dominated by green cluster with minor appearance of other clusters. From 3 μs till ~9.5 μs the trajectory was very dynamic and showed inter-conversion of yellow, purple, black, blue, green and cyan clusters. The last 0.3 μs of the trajectory showed the orange cluster which was not observed in any other part of the trajectory. A comparison of structures representing each cluster shows that the major differences are observed in the position of propeller loop bases A5 and C9, C11 of lateral loop and A19 of LP loop (Supplementary Figure S11). The base T12 of lateral loop shows limited movements as it interacts with A1 and stacks with G10 and G13 of the first quartet during the simulation.

Supplementary Table 1: Percentage of appearance of each cluster in the 10 μs long Simulation **1** carried in presence of excess sodium cations.

**Cluster number**	**Color for representation of Cluster**	**Percentage in trajectory (%)**	**RMSD with respect to NMR model 1 (Å)**	**RMSD with respect to quadruplex B of 3QXR crystal structure (Å)**
Cluster 1	Grey/Black	18.4	1.935	2.227
Cluster 2	Green	38.8	1.988	2.213
Cluster 3	Yellow	23.1	1.902	2.401
Cluster 4	Blue	7.4	1.881	2.456
Cluster 5	Orange	2.2	2.223	2.602
Cluster 6	Brown	7.0	1.413	2.135
Cluster 7	Red	0.16	1.828	1.427
Cluster 8	Purple	0.44	1.716	2.155
Cluster 9	Cyan	2.5	2.250	2.539

MM-PBSA results

The LP loop exhibits two distinct conformations in the MD simulations. One in which the loop is close to the adjacent quartet and the cleft is narrow as in the X-ray structure and second in which the loop is further away from the adjacent quartet and the cleft is broad as in NMR structure. We used 7.450-7.550 μs simulation snapshots from the Simulation **1** to evaluate the effect of loop conformation on the GQ energy. During this period the desired movement of LP loop is sampled in the trajectory. The energy calculation by MM-PBSA revealed that there is no significant difference in energy of the GQ conformations when the stem loop is close to adjacent quartet (similar to the crystal structure) and when the stem loop is relatively further away from the adjacent quartet (similar to the NMR structure) (Supplementary Figure S22). The MM-PBSA method has limitations and individually minimized structures from the same trajectory might show a variation of 5 kcal/mol (Supplementary reference 3). Our ensembles also show a variation of ~5-10 kcal/mol and hence we predict that the two loop conformations are isoenergetic and represent two different converged states of *c-kit* promoter GQ. The intrinsic inaccuracy of the MM-PBSA method does not allow us to make any more specific analyses. In other words, the energy difference we wanted to derive is too small compared to the intrinsic accuracy limits of the method.

Cation binding to exterior of *c-kit* promoter GQ in the bsc0$\chi_{OL4}\varepsilon\zeta_{OL1}$ simulation and in K^+ simulation

Supplementary Table 2: Cation binding sites in the *c-kit* promoter GQ observed in Simulations **2** and **3** (the upper and lower line for each site, respectively). The percent occupancy was calculated by dividing the number of frames in which any ion is at a distance of 3 Å from the respective site by the total frames of the trajectory multiplied by 100. Note that in Simulation **2**, C11 adopts a specific conformation (see the main text) which is not seen in the other simulations. It is associated with a specific ion binding site.

Residues	Atoms of the GQ forming the cation binding site	Cation	Binding times (ns)[a]	Occupancy during the simulation (%)	Observed in the crystal structure
A5	N7 of A5 and sugar phosphate backbone of A5	Na^+	2 to 62	14	No
	O4' of A5 and sugar phosphate backbone of G7	K^+	2 to 25	17	Yes
C9	phosphate oxygen atom of C9 and G21	Na^+	2 to 1000	56	No
		K^+	1 to 125	55	No
C11, G10 and G21	Sugar phosphate backbone of C11, G10 and G21	Na^+	1 to 100	10	Yes
		K^+	2 to 300	53	Yes
C11 and G13	O2 of C11 and phosphate G13	Na^+	2 to 800	63.4	No
A16	N7 of A16 and carbonyl oxygen of G17	Na^+	1-90	18	Yes
		K^+	1-40	25	Yes
A19	N3 of A19	Na^+	1-80	17.5	No
		K^+	1-40	30	No

[a]Length (its range) of the individual binding events.

Additional no-salt simulations in TIP3P water model (Simulations 10 a-c) not shown in the main text.

In Simulation **10a**, the unfolding is initiated by the movement and misalignment of strand **b** at 3 ns (Supplementary Figure S41a). Strand **a** undergoes vertical slippage at ~8 ns followed by loss of base pairing with all the bases of the neighbouring strands at ~8.5 ns. The conformations of single nucleotide loops A5 and C9 are simultaneously lost in this simulation, due to the above movements of the strands **a** and **b.** Essentially, misalignment of strand **a** is sufficient to perturb

the structure of the propeller loops. G10 undergoes vertical strand slippage in strand **c** and conformation of also lateral loop is also lost at 11 ns. Later, G21 and G22 of strand **c** tilt to form stacking interactions with bases of strand **b**. This arrangement of stacked bases lasts till the end of the simulation. The conformation of LP loop is retained till the end of 100 ns of the simulation.

The unfolding in Simulation **10b** is also initiated by misalignment of strand **b** of the GQ at ~30 ns of the simulation (Supplementary Figure S41b). This misalignment destabilizes the preceding propeller loop A5, its structure is then lost. The strand **a** loses base-pairing with both the neighbouring strands at 60 ns. The native base pairing between G21 and G22 of strand **c** and respective bases of strand **d** (G14, G15) is retained till the end of 100 ns of the simulation.

In Simulation **10c**, strand **a** undergoes vertical slippage at ~ 4 ns which destabilizes the conformation of propeller loop A5 and strand **b** (Supplementary Figure S41c). This leads to misalignment of strand **b** along the G-stem and simultaneous loss in conformation of single residue propeller loop C9. Then, the native base pairing interactions between strands **a** and **d** are lost. This simulation was terminated at 15 ns. These three simulations confirm the basic picture of early stages of unfolding described in the main text.

Additional no-salt simulation in SPC/E water model (Simulations 11 a-c) not shown in the main text

In Simulation **11a**, the unfolding is initiated by collapse of strand **b** and vertical slippage of strand **a** similar to that observed in unfolding simulations carried out in TIP3P water model (Supplementary Figure S42a). The snapback bases G21 and G22 of strand **c** also lose native interactions with strand **d.** These bases realign within few nanoseconds and LP loop is then stable till end of the 150 ns of this no-salt simulation.

The unfolding in Simulation **11b** is initiated by misalignment of strand **b** at 10 ns (Supplementary Figure S42b). The bases G10 and G21 of strand **c** also lose the native interactions leading to vertical slippage at ~22 ns. Strand **a** undergoes vertical slippage by one base in the direction below the third quartet at 90 ns.

In the unfolding Simulation **11c**, strand **b** collapses at 50 ns (Supplementary Figure S42c). Simultaneously strand **c** collapses and stacks with bases of the misaligned strand **b**. The confirmation and intra-loop interactions of LP loop are retained in this simulation. Strand **a** undergoes vertical slippage at ~190 ns**.** Overall, the unfolding pathways in the present simulations are broadly similar in TIP3P and SPC/E water models; there is no indication of any systematic effect of the water model on the unfolding processes. The misalignments and expulsions of strands **a** and **b** highlight the weakness of single nucleotide propeller loops.

Supplementary References

1. Feig, M., Karanicolas, J. and Brooks, C.L. (2004) MMTSB Tool Set: enhanced sampling and multiscale modeling methods for applications in structural biology. *J. Mol. Graph. Model.*, **22**, 377-395.

2. Tan, C., Yang, L. and Luo, R. (2006) How Well Does Poisson−Boltzmann Implicit

Solvent Agree with Explicit Solvent? A Quantitative Analysis. *J. Phys. Chem. B*, **110**, 18680-18687.
3. Homeyer, N. and Gohlke, H. (2012) Free Energy Calculations by the Molecular Mechanics Poisson−Boltzmann Surface Area Method. *Mol. Inform.*, **31**, 114-122.

LEGENDS TO SUPPLEMENTARY MOVIES

Supplementary movie 1: The movie shows unfolding pathway of *c-kit* promoter GQ in no-salt (unfolding) Simulation **6.** The major events of unfolding are shown in the movie. GQ is shown in tube representation. Stem guanines are also shown in licorice and ribbons representation. Strand **a** is shown in blue, strand **b** in yellow, strand **c** in green and strand **d** in orange. The backbone of loops and terminal base (A1) is shown in tan. Hydrogens are not shown.

Supplementary movie 2: The movie shows unfolding pathway of *c-kit* promoter GQ in no-salt Simulation **8.** The major events of unfolding are shown in the movie. The coloring scheme is explained in legend to Supplementary movie 1.

Supplementary movie 3: Major events in the refolding of snapshot at 284 ns (Simulation **9c**) taken from unfolding Simulation **8** are presented in the movie. The coloring scheme is explained in legend to Supplementary movie 1. Hydrogens and ions are not shown. Refolding is initiated by realignment and slippage of strand **a** at ~300ns. Strand **d** also realigned to form native base pairings by ~450 ns. Overall, significant refolding from a highly perturbed starting structure was observed in this simulation. Among the quartet bases, only G4 is slightly tilted and could not re-attain native base pairings. The simulation was continued for 2 μs but alignment of G4 in the third quartet was not achieved. This is nevertheless the most successful refolding event in our study and a quite unique atomistic documentation of processes that can take place in the very last stages of folding of the individual GQ molecules, or during unsuccessful unfolding attempts of individual GQ molecules in their folded ensembles.

List of attached PDB files

Five snapshots from first unfolding simulation (Simulation **6**) at 9, 59, 132, 424 and 500 ns:

1. 9ns_simulation6
2. 59ns_simulation6
3. 132ns_simulation6
4. 424ns_simulation6
5. 500ns_simulation6

Six snapshots from second unfolding simulation (Simulation **8**) at 32, 100, 284, 394, 453 and 500 ns:

1. 32ns_simulation8
2. 100ns_simulation8
3. 284ns_simulation8

4. 394ns_simulation8
5. 453ns_simulation8
6. 500ns_simulation8
