# Supplementary material for: Extended molecular dynamics of a c-kit promoter quadruplex
Source: Nucleic Acids Res. 2015 Oct 10;43(18):8673–93. doi: 10.1093/nar/gkv785 (PMC4605300; doi:10.1093/nar/gkv785)
Supplement: SUPPLEMENTARY DATA [file supp_gkv785_nar-00989-f-2015-File011.pdf]

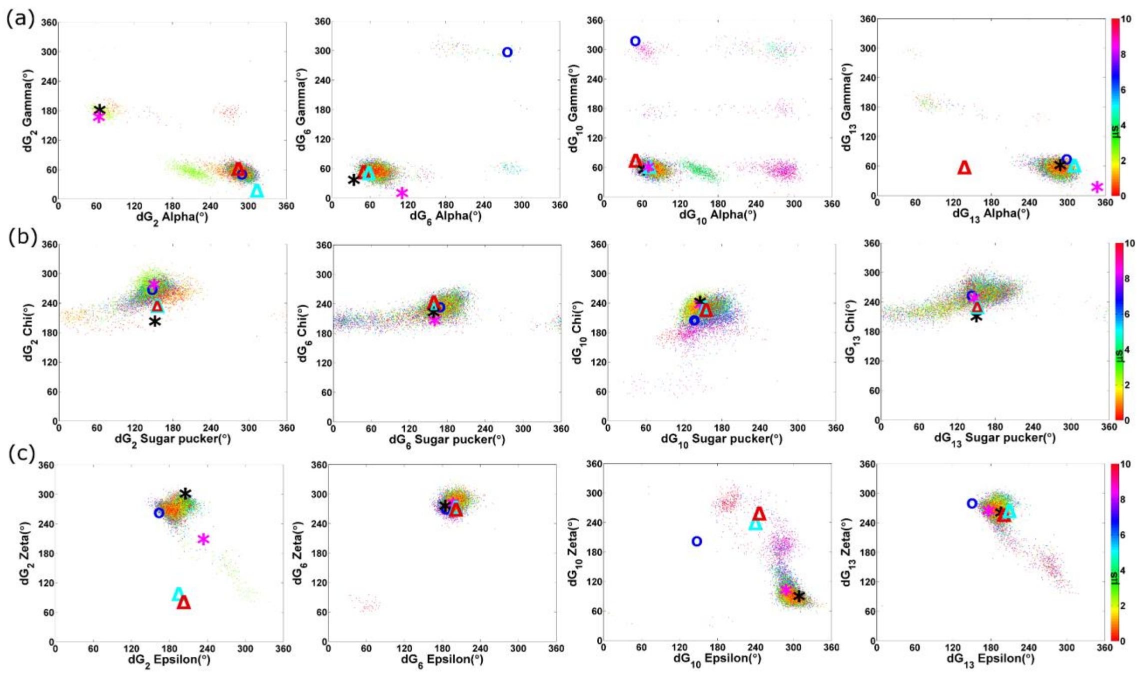

Figure S1: Scattergrams of dihedral angles of the G2:G6:G10:G13 (quartet 1) of *c-kit* promoter GQ observed in the 10 µs long Simulation **1**. (a) α/γ, (b) χ vs sugar pucker and (c) ε/ζ are shown. The pink and black asterisk, cyan and red triangle and blue circle in the figures indicate values observed in GQ A and B of X-ray crystal structure 3QXR, GQ A and B of X-ray crystal structure 4WO2 and model 1 of NMR structure 2O3M, respectively. The color bar indicates time in microseconds.

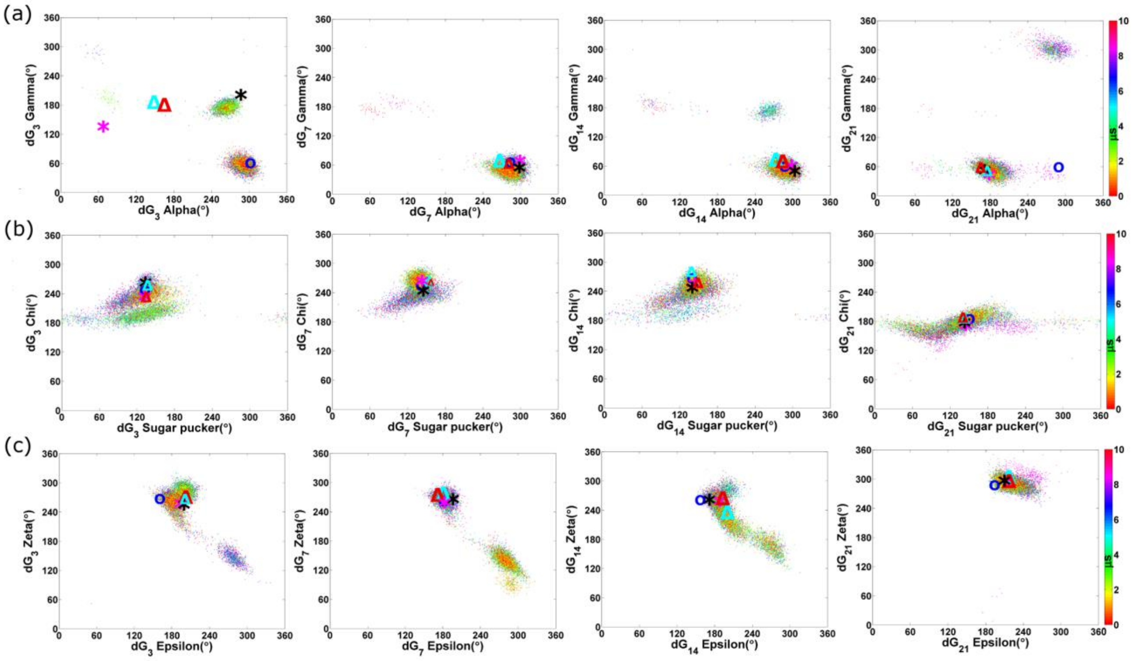

Figure S2: Scattergrams of dihedral angles of G3:G7:G21:G14 (quartet 2) of *c-kit* promoter GQ observed in the 10 µs long Simulation **1**. (a) α/γ, (b) χ vs sugar pucker and (c) ε/ζ are shown. The symbols are described in the legend to Figure S1.

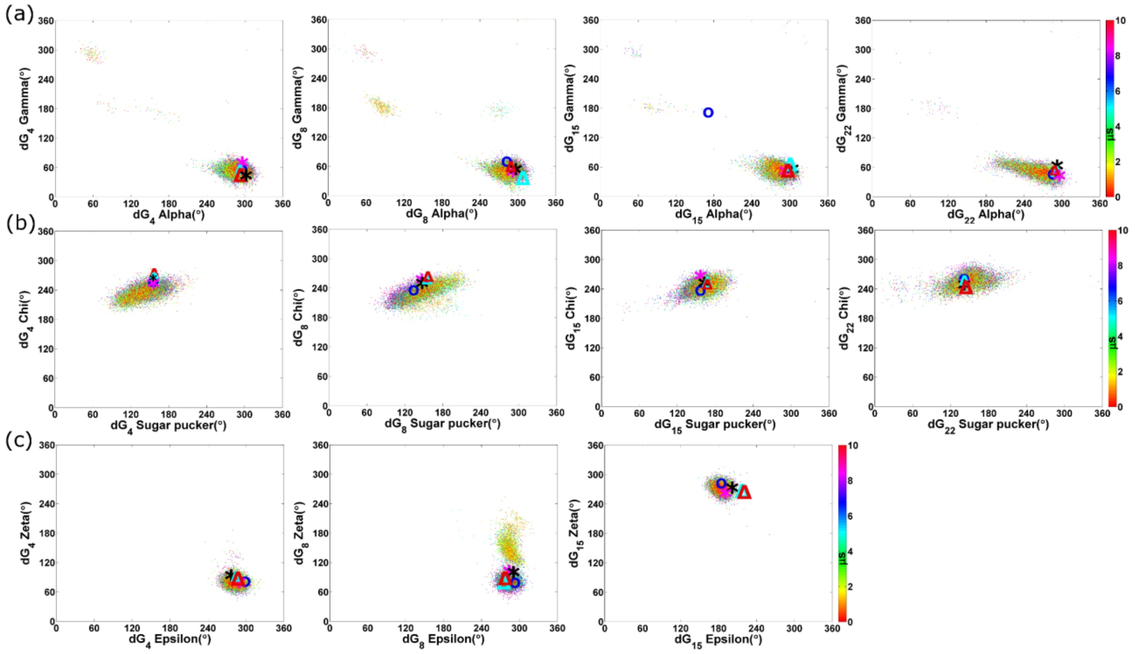

Figure S3: Scattergrams of dihedral angles of G4:G8:G22:G15 (quartet 3) of *c-kit* promoter GQ observed in the 10 μs long Simulation **1**. (a) α/γ, (b) χ vs sugar pucker and (c) ε/ζ are shown. The symbols are described in the legend to Figure S1.

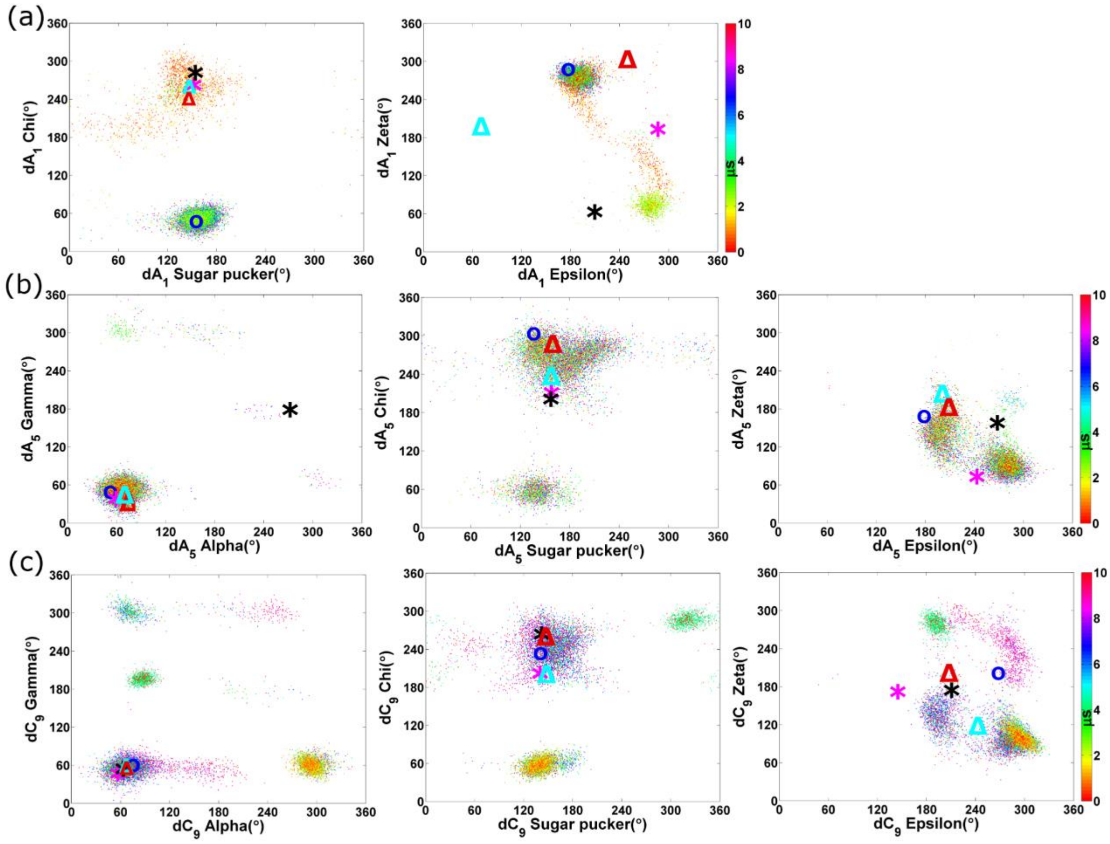

Figure S4: Scattergrams of dihedral angles of (a) terminal base A1 (b) single nucleotide propeller loop A5 and (c) single nucleotide propeller loop C9 of *c-kit* promoter GQ observed in the 10 µs long Simulation **1**. The α/γ, χ vs sugar pucker and ε/ζ are shown. The symbols are described in the legend to Figure S1.

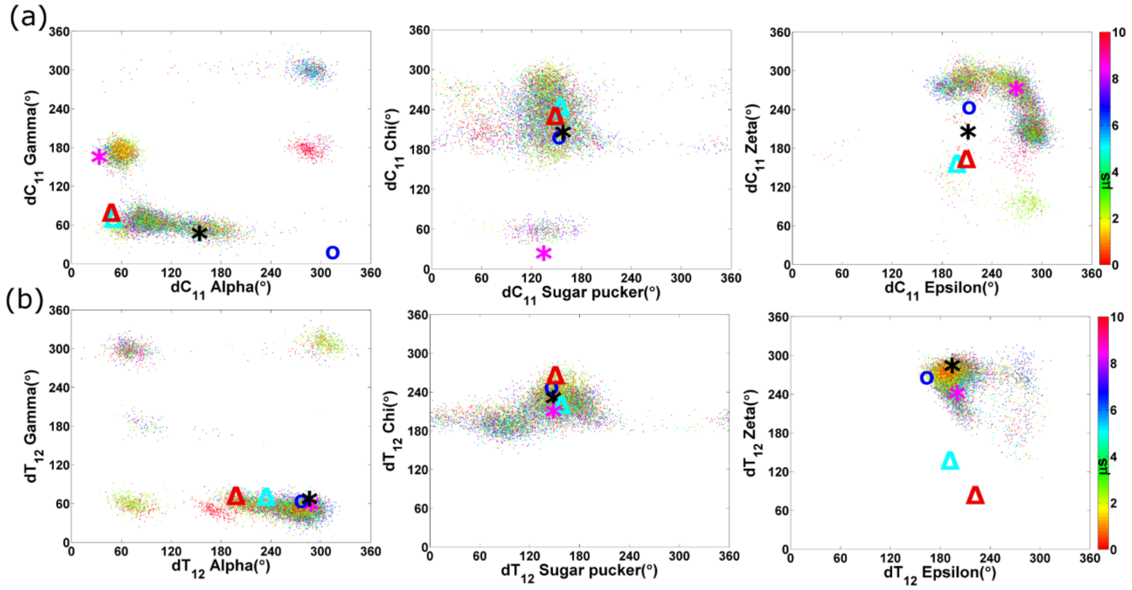

Figure S5: Scattergrams of dihedral angles of lateral loop bases (a) C11 and (b) T12 of *c-kit* promoter GQ observed in the 10 μs long Simulation **1**. The α/γ, χ vs sugar pucker and ε/ζ are shown. The symbols are described in the legend to Figure S1.

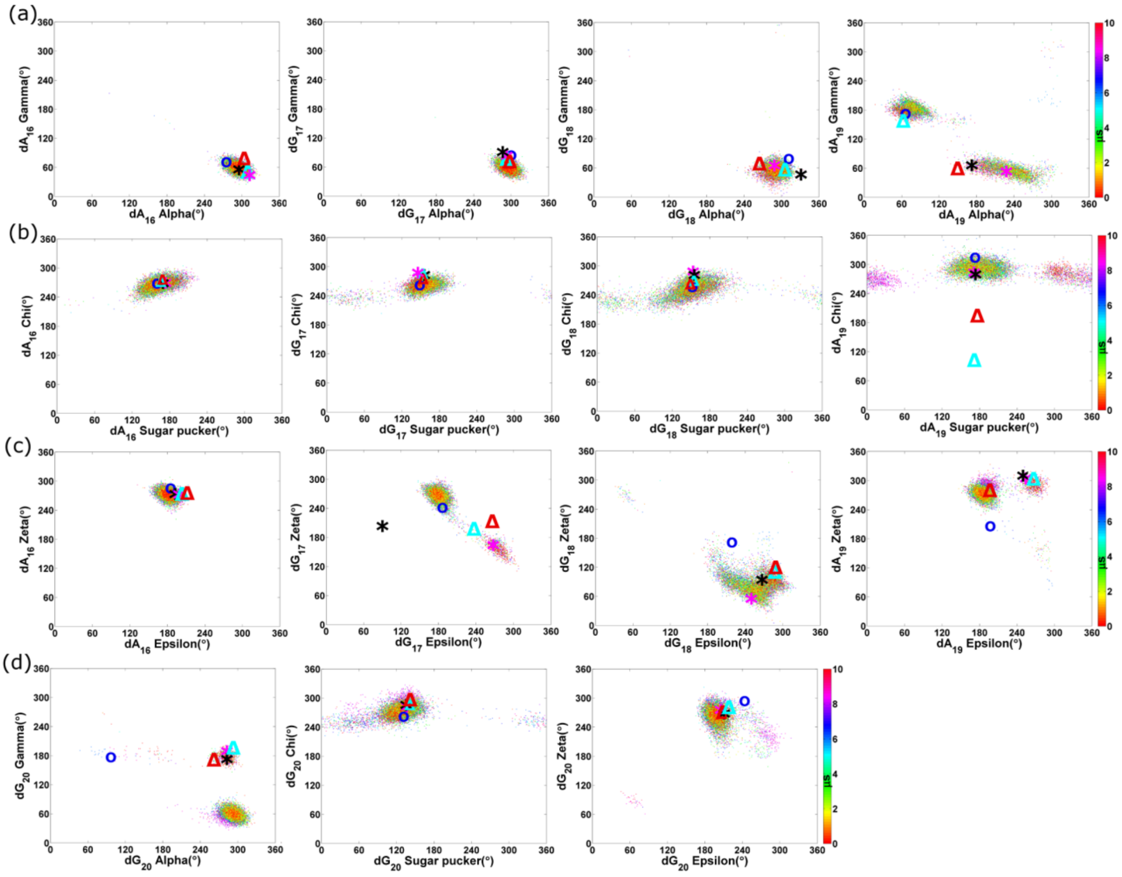

Figure S6: Scattergrams of dihedral angles of LP loop of of *c-kit* promoter GQ observed in the 10 μs long Simulation **1**. The (a) α/γ, (b) χ vs sugar pucker, (c) ε/ζ of the A16, G17, G18, and A19 are shown. (d) shows dihedral angles of G20. The symbols are described in the legend to Figure S1.
