# Supplementary material for: Extended molecular dynamics of a c-kit promoter quadruplex
Source: Nucleic Acids Res. 2015 Oct 10;43(18):8673–93. doi: 10.1093/nar/gkv785 (PMC4605300; doi:10.1093/nar/gkv785)
Supplement: SUPPLEMENTARY DATA [file supp_gkv785_nar-00989-f-2015-File012.pdf]

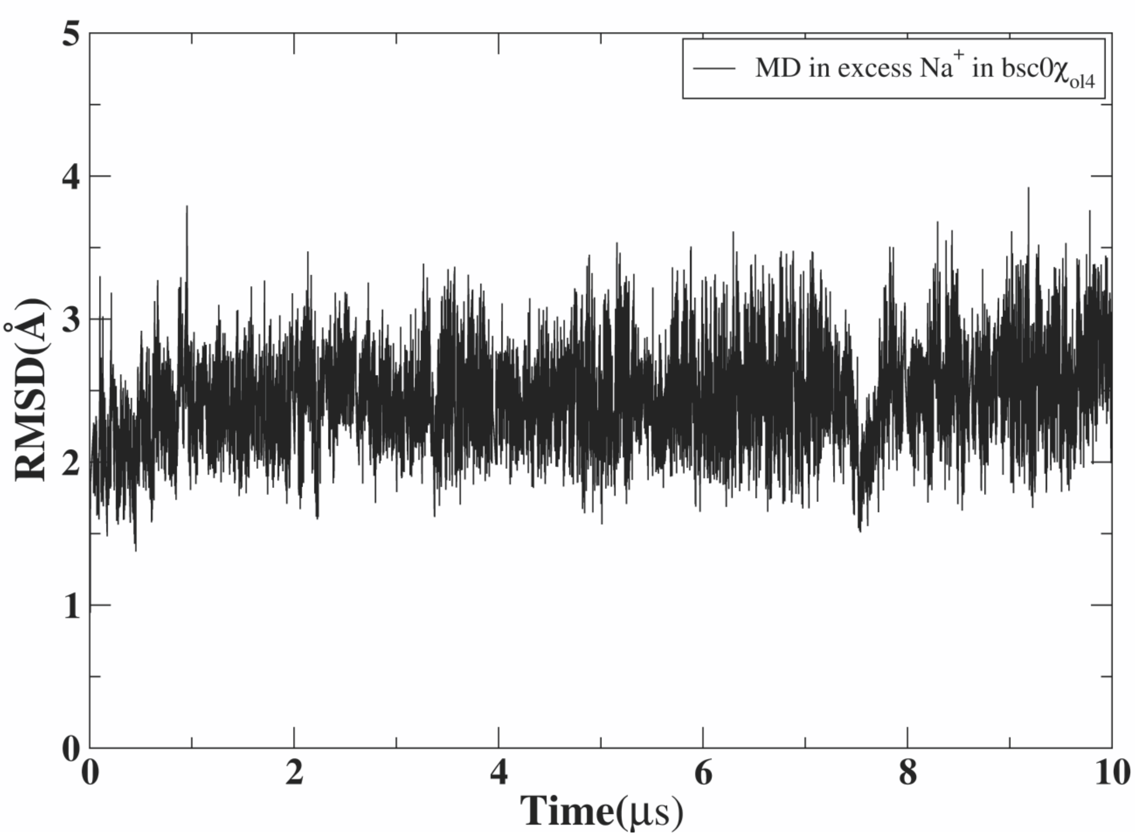

Figure S7: RMSD of *c-kit* promoter GQ over 10 μs in MD simulation carried out in presence of excess Na^+ in bsc0χ_{OL4} force-field (Simulation **1**). Although RMSD is not a very representative measure of the simulation behavior, the dependence shown above indicates a very stable trajectory with minor fluctuations.

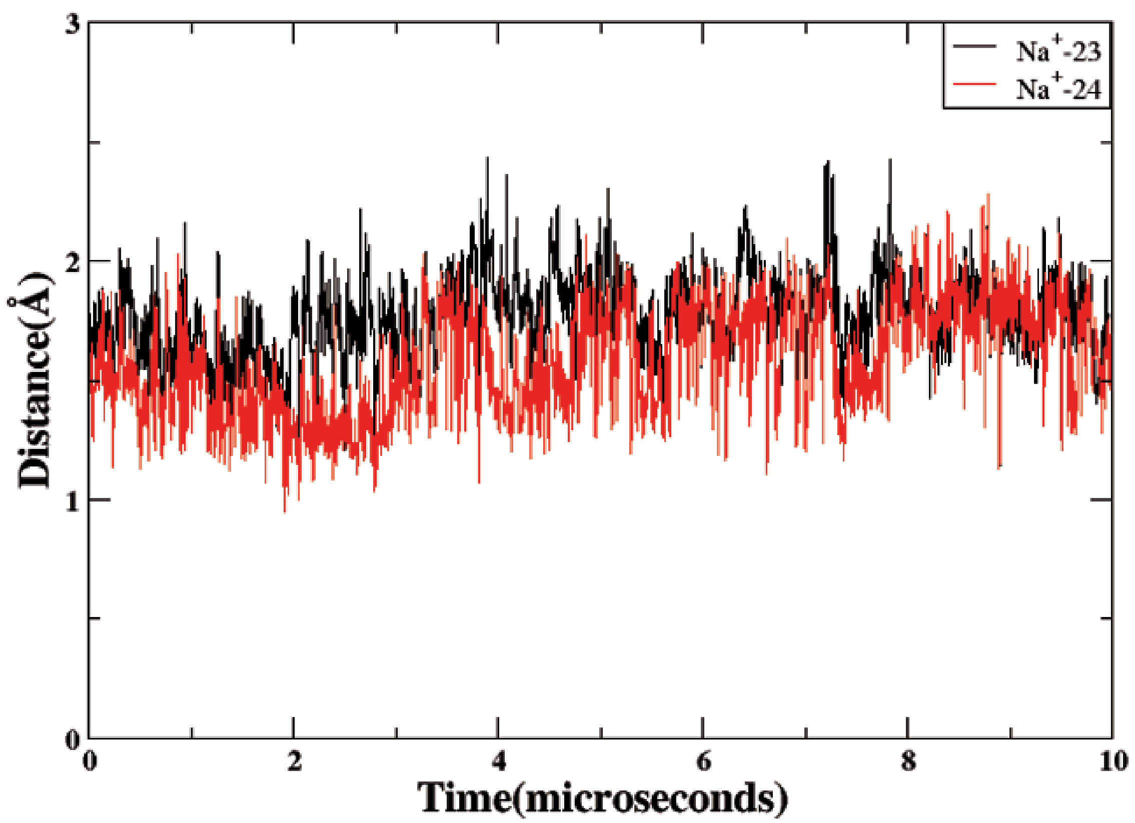

Figure S8: Distance of channel Na^+ ions in the 10 μs long Simulation **1**. The distance of each cation was measured from center of mass of GQ stem bases. The black line represents distance of cation residing between the first and the second quartet while the red line represents distance of cation between the second and the third. The bound ions can be considered as perfectly stable.

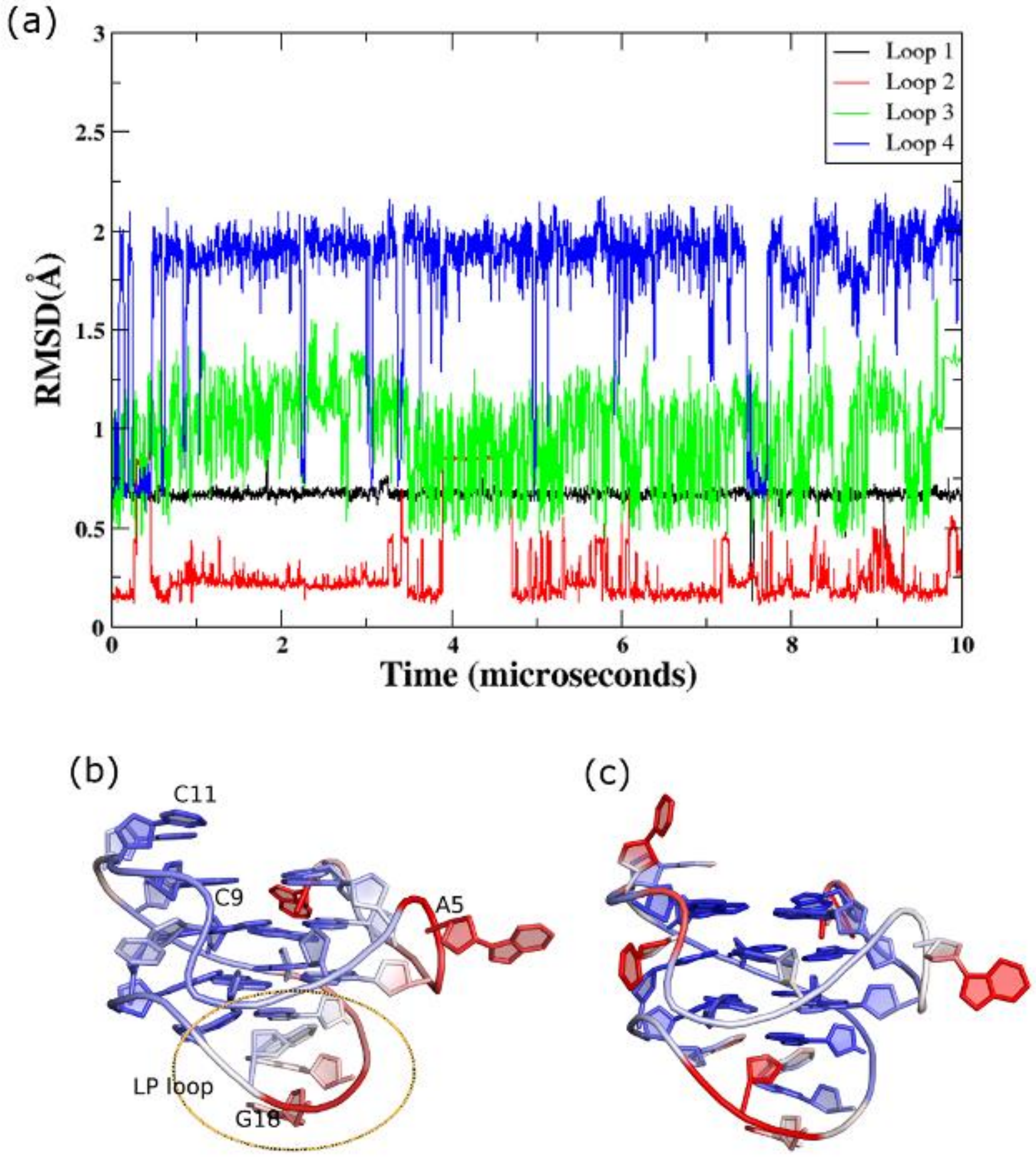

Figure S9: (a) RMSD of sugar phosphate backbone (with respect to the start) of all the four loops of GQ in the 10 µs long Simulation **1** in excess Na^+. The backbone RMSD of single residue propeller loop A5 (loop 1) is shown in black, C9 is shown in red (loop 2), lateral loop C11-T12 (loop 3) is shown in green and LP loop A16-G20 (loop 4) is shown in blue. A comparison of atomic fluctuations in (b) GQ B of crystal structure 3QXR and (c) Simulation **1** is shown in cartoon representation. The mobility distribution was calculated using B-factors from the crystal structure and averaged RMSF from the MD simulation. The structures are colored according to mobility distribution with blue-white-red spectrum representing lowest to highest fluctuating atoms.

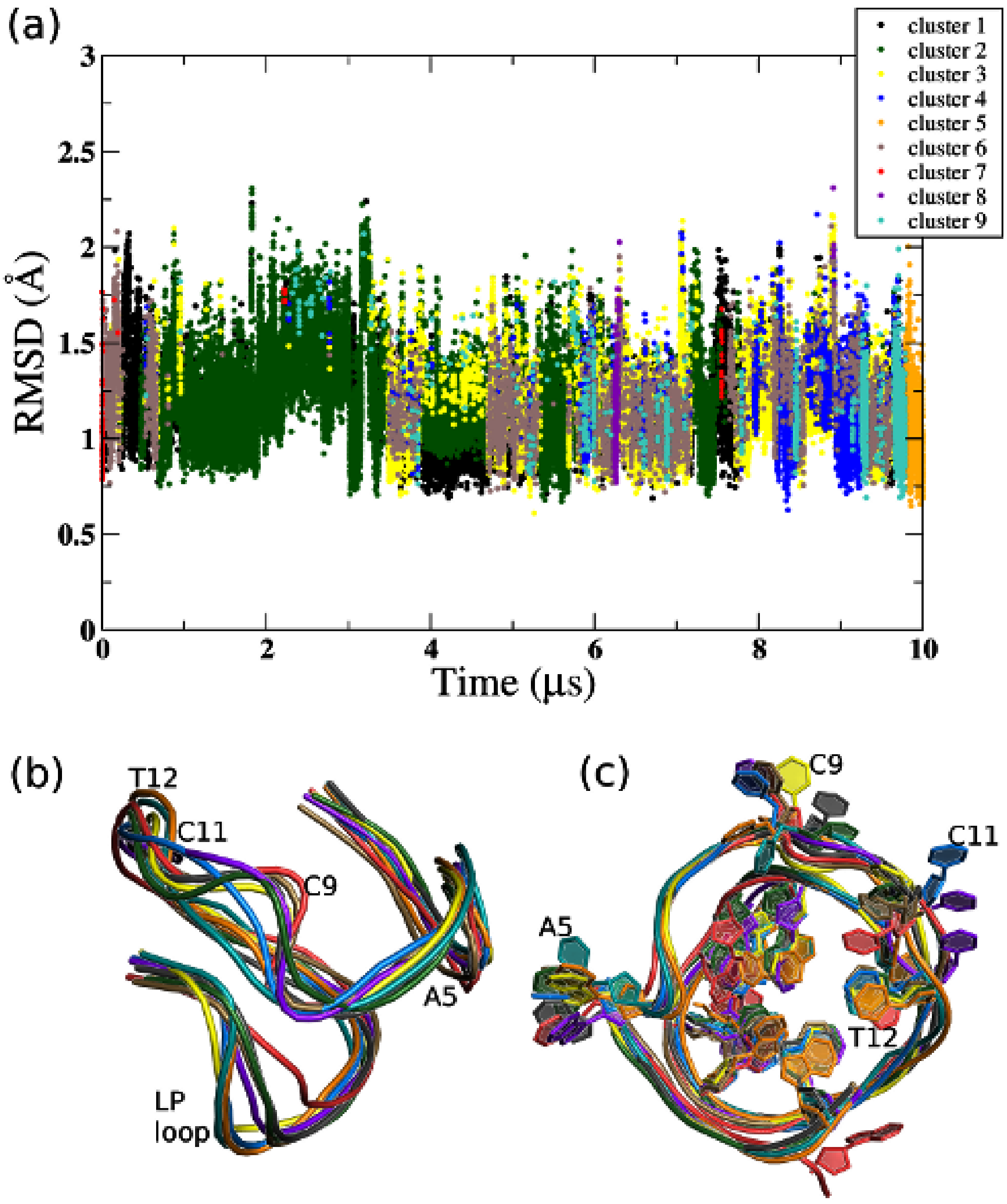

Figure S10: (a) Conformational transitions in the Simulation **1** of *c-kit* promoter GQ identified via clustering analysis. All atom weighted RMSD-based clustering was carried out using a cut-off of 2.4 Å over the trajectory. Nine clusters were sieved out from the 10 µs long Simulation **1**. Overlay of the backbone of medoid structures representing the clusters. (b) Side view and (c) Top view with loop bases in cartoon representation. Clusters 1-9 are represented by different colors.

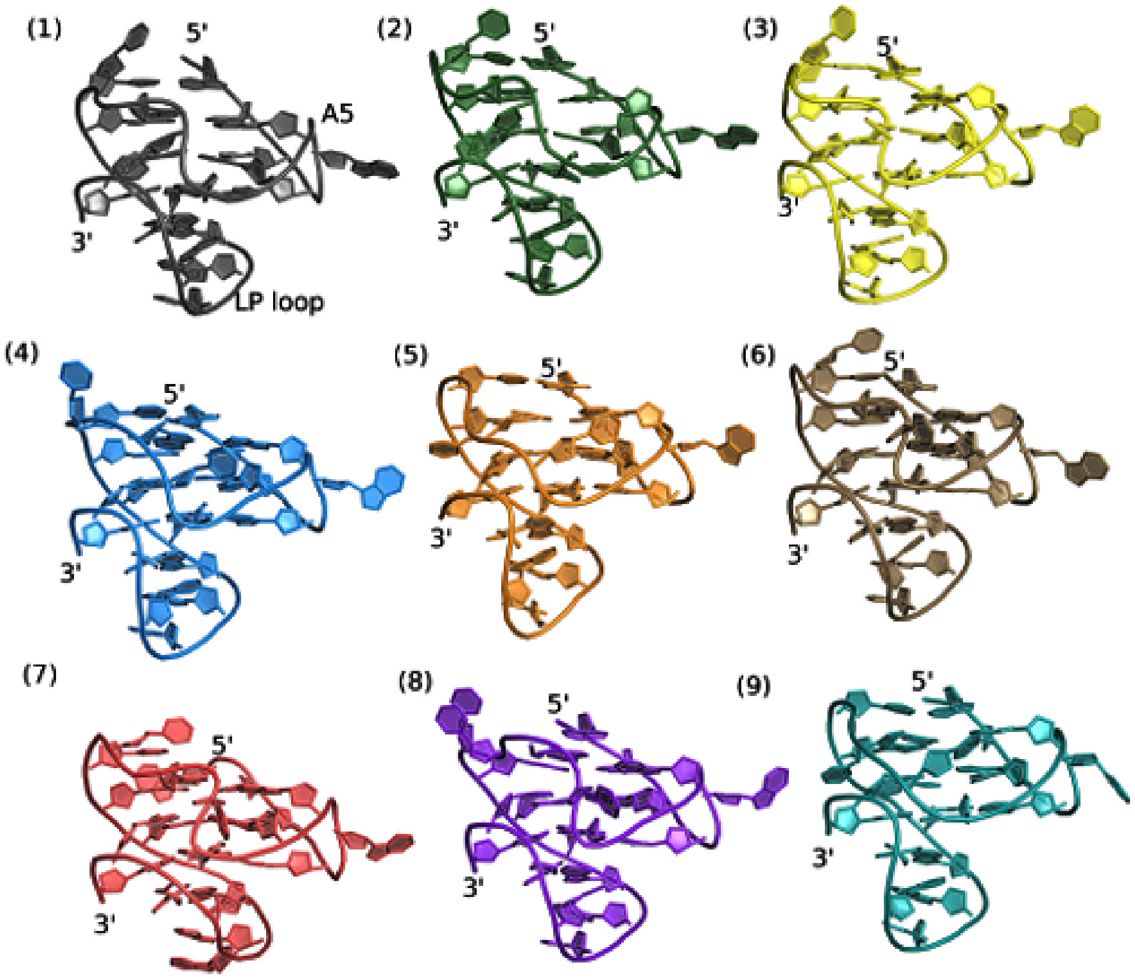

Figure S11: Cartoon representations of the nine clusters identified from the 10 μs long trajectory of Simulation **1** carried out in presence of Na^+ ions in the bsc0χ_{OL4} force-field using an all atom RMSD cut-off of 2.4 Å. The cluster 7 is closest to the starting structure in the simulation and also appears briefly at ~2.2 and ~7.5 μs of Simulation **1**.

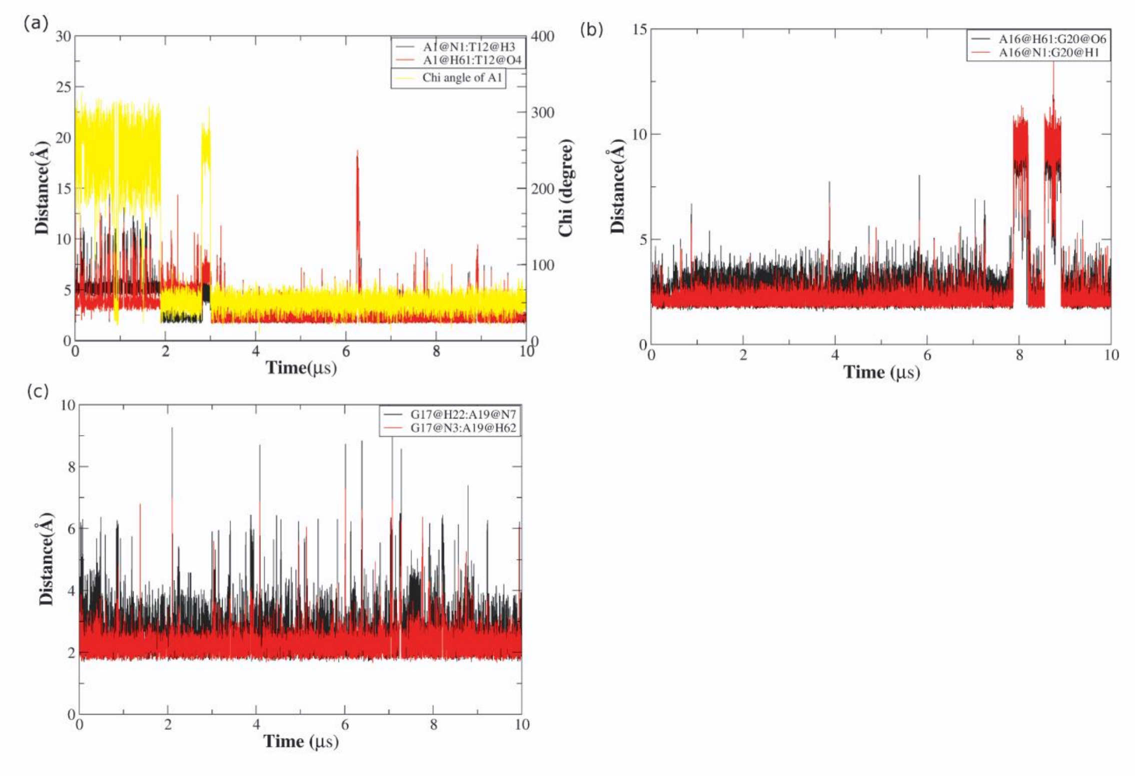

Figure S12: Distance plot of base pairs found within the loops of *c-kit* GQ in the 10 µs long Simulation **1** carried out in presence of Na^+ in the bsc0χ_{OL4} force-field. (a) The A1 and T12 *cis* WC base pair together with χ angle values of A1 (yellow) are shown. The A1 and T12 base pair is only formed when A1 is in *syn* orientation. (b) A16 and G20 form WC pair and (c) G17 and A19 form sheared base pair.

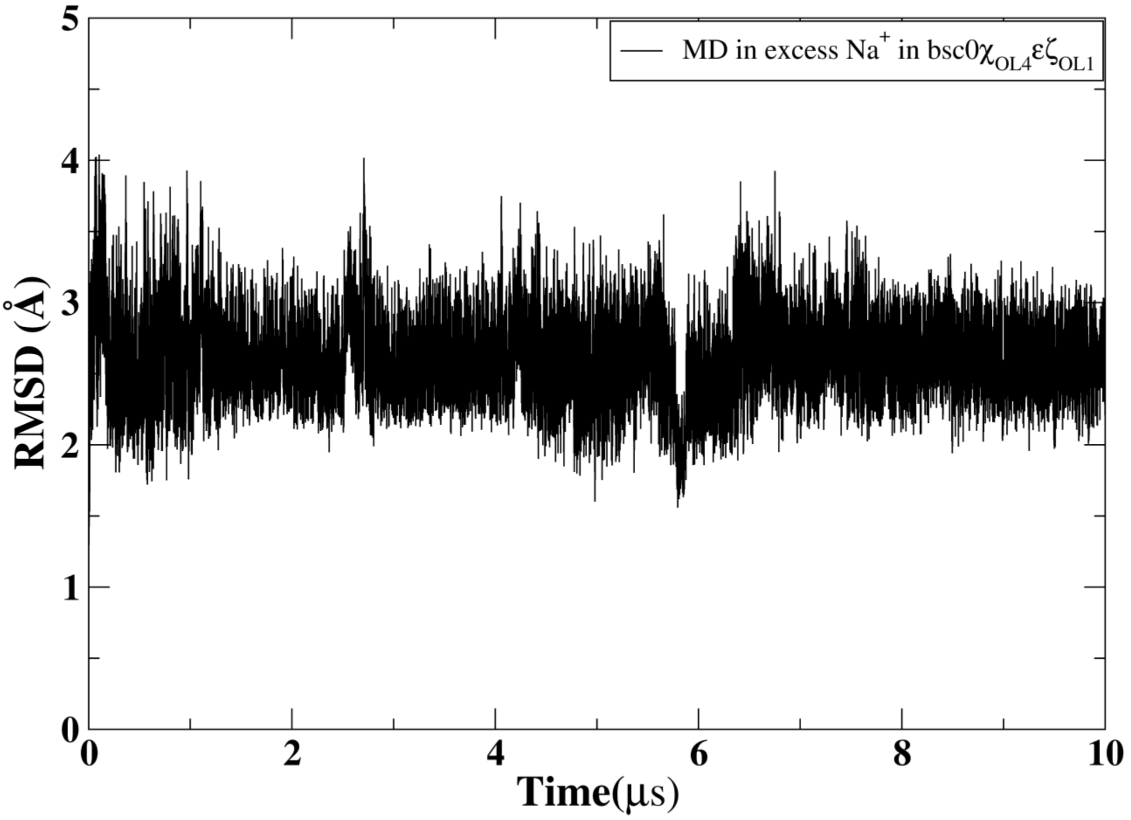

Figure S13: RMSD of *c-kit* promoter GQ over 10 μs in MD simulation carried out in presence of excess Na^+ in the bsc0$\chi_{OL4}\varepsilon\zeta_{OL1}$ force-field (Simulation **2**).

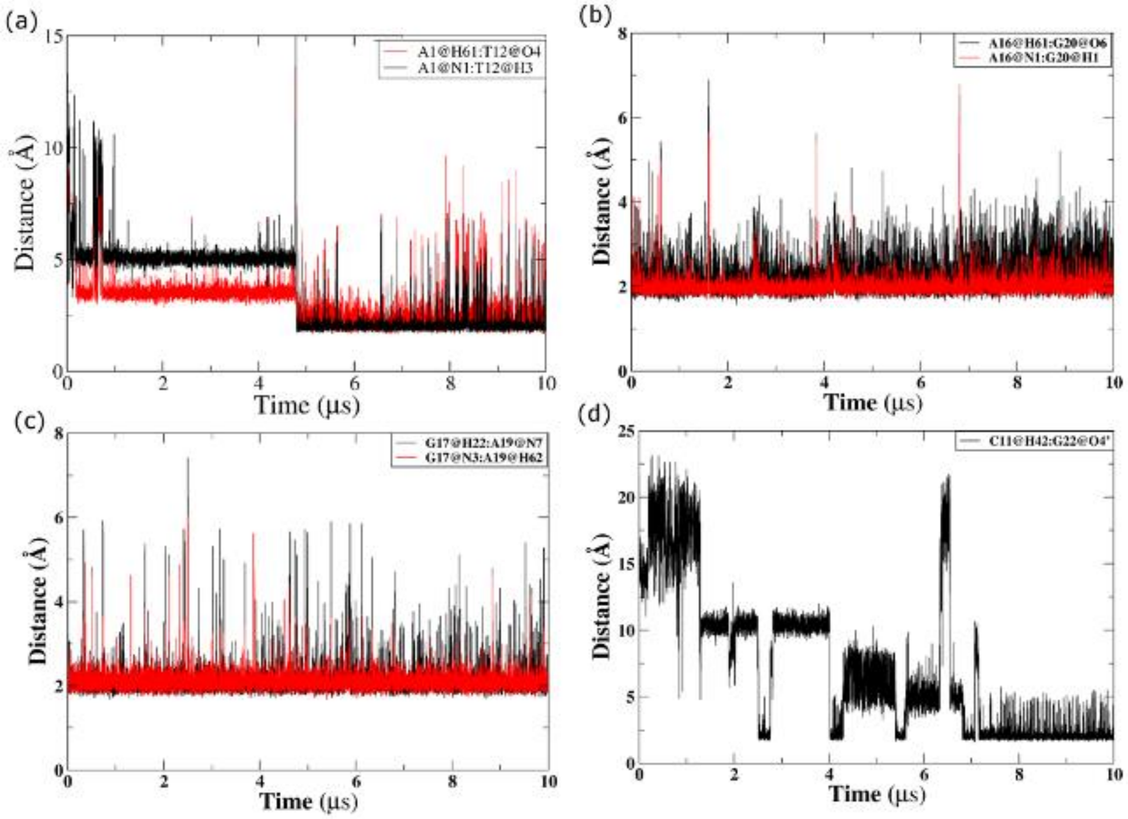

Figure S14: Distance plot of base interactions found in *c-kit promoter* GQ 10 µs long Simulation **2** in presence of excess Na^+ in the bsc0$\chi_{OL4}\varepsilon\zeta_{OL1}$ force-field. (a) A1 and T12 *cis* WC is formed after ~4.8 µs when A1 goes to *syn* orientation. (b) A16 and G20 pair, (c) G17 and A19 pair and (d) C11 bonds with O4' of G22 through its amino hydrogen at ~6.8 µs; it locks the lateral loop in one conformation till the end of the simulation.

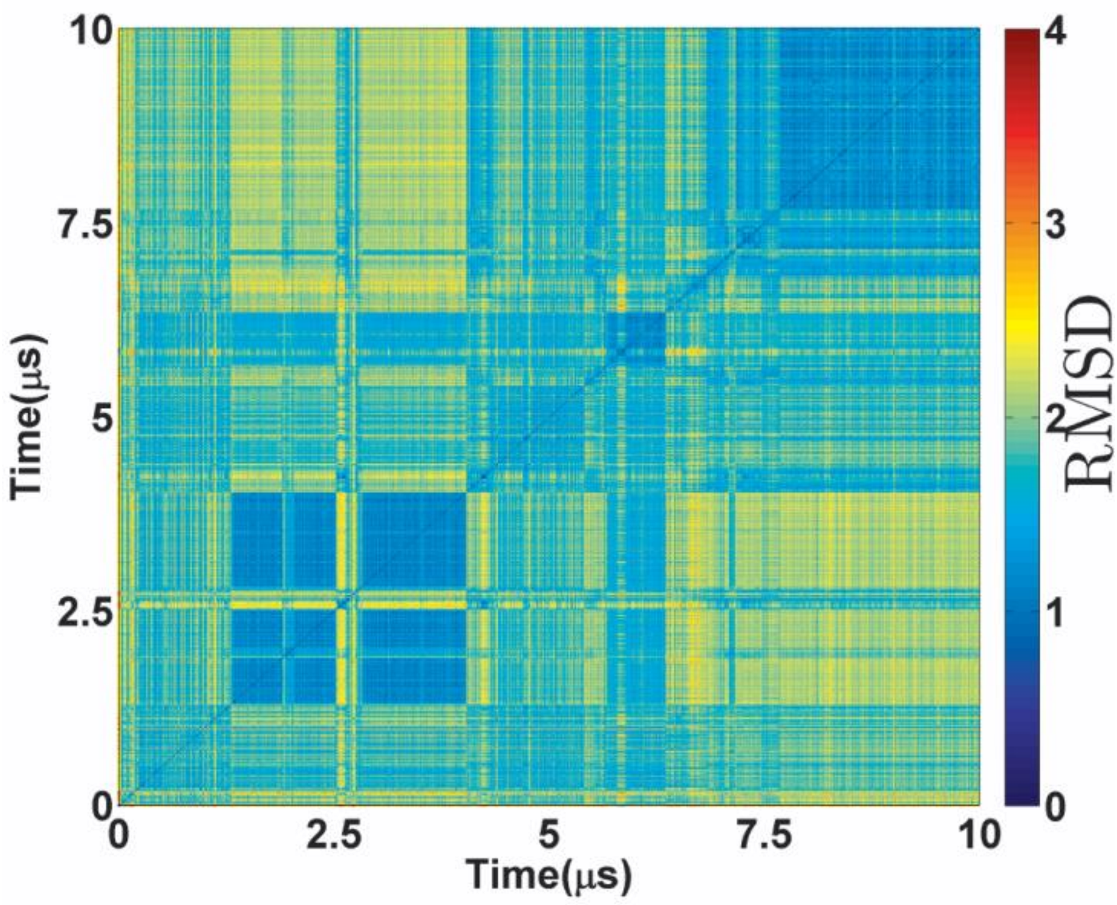

Figure S15: 2D-RMSD of backbone atoms of *c-kit* promoter quadruplex over 10 μs in the Simulation **2** carried out in the bsc0$\chi_{OL4}\varepsilon\zeta_{OL1}$ force-field. The Figure visualises the data with δt = 5 ns resolution.

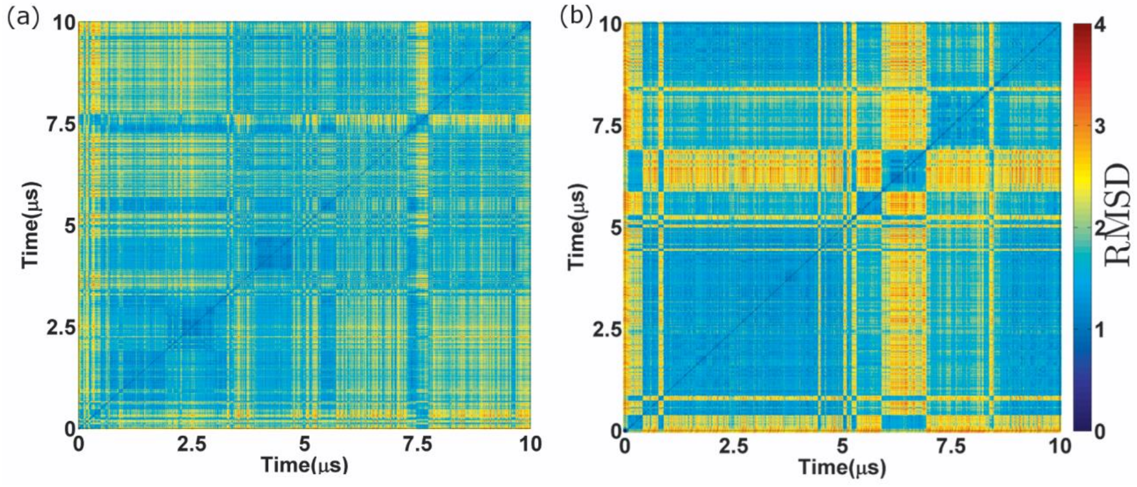

Figure S16: Comparison of 2D-RMSD of backbone atoms in 10 µs long (a) Na^+ and (b) K^+ simulation in the bsc0χ_{OL4} force-field, Simulation **1** vs. Simulation **3**. The Figure visualises the data with δt = 5 ns resolution.

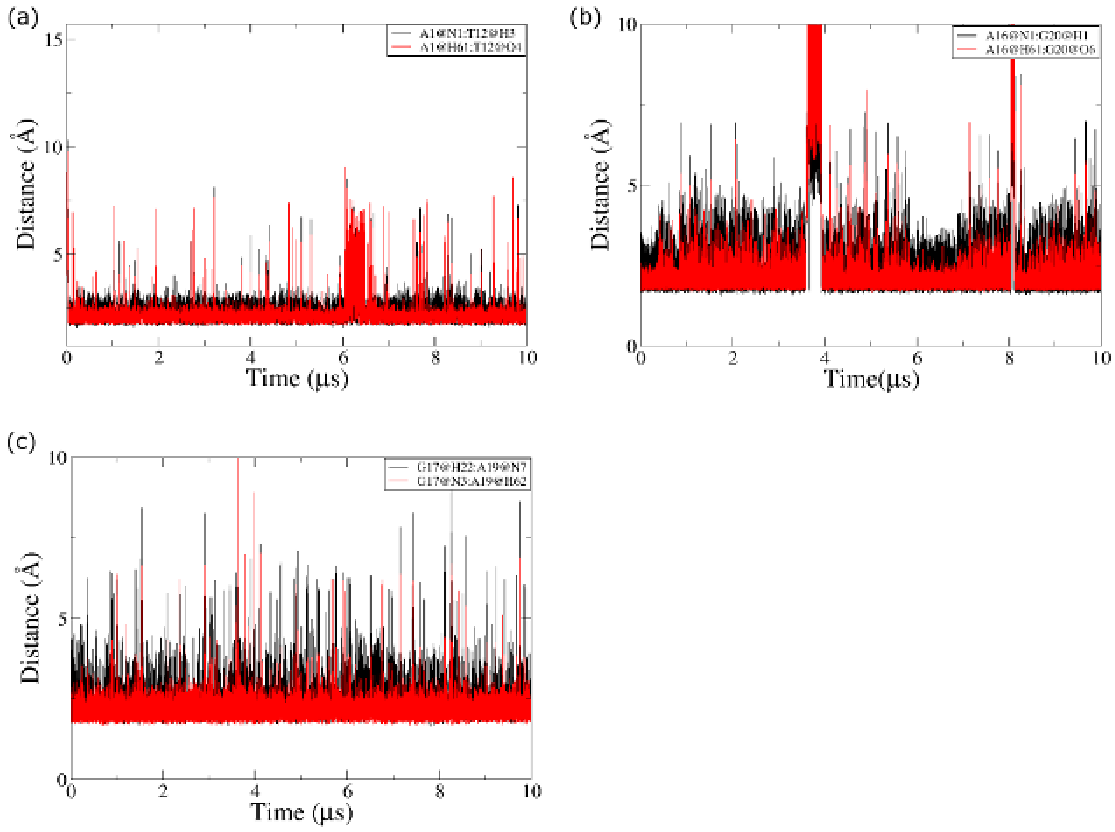

Figure S17: Distance plots of base pairing found within the loops of *c-kit* promoter GQ in 10 µs long simulation in presence of excess K^+ in the bsc0χ_{OL4} force-field (Simulation **3**). (a) A1 and T12 pair, (b) A16 and G20 pair and (c) G17 and A19 base pair similar to the experimental structure were sampled in the simulation.

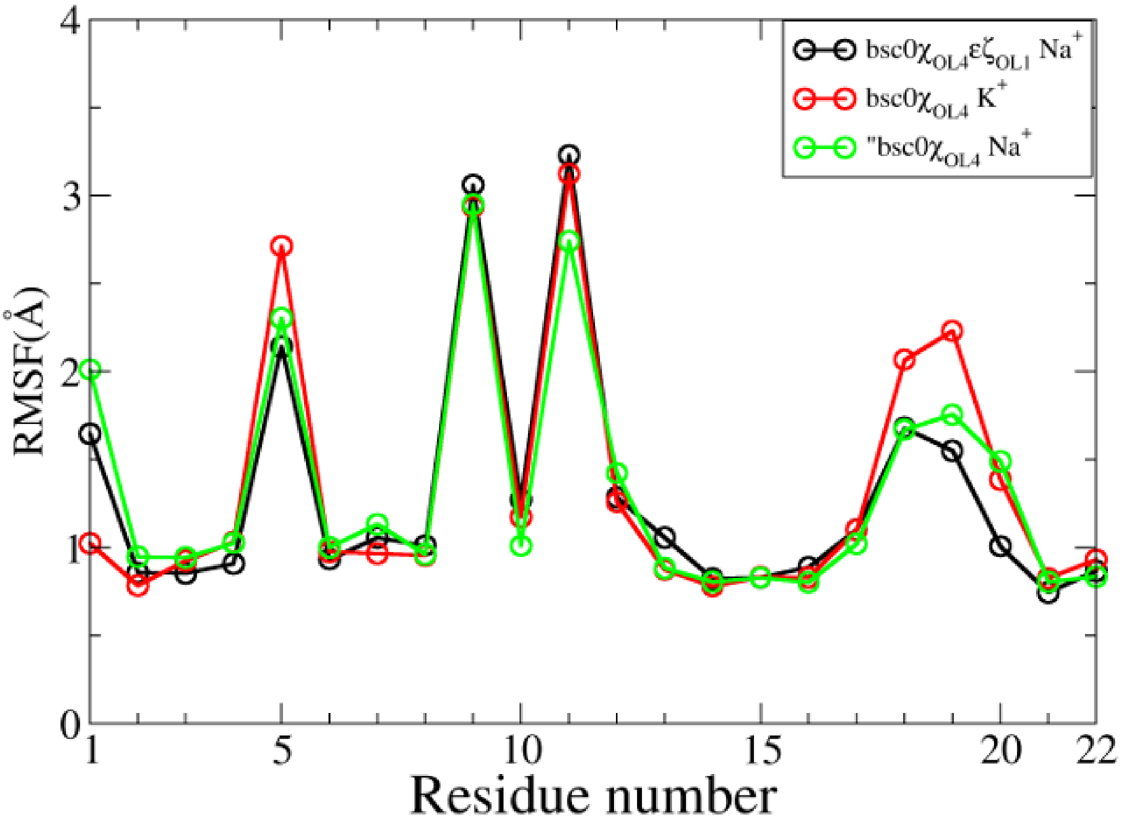

Figure S18: Average root mean squared fluctuations (RMSF) of GQ residues in Simulations **1**, **2** and **3**. The RMSF of residues in Simulation **1** is shown in green, Simulation **2** in black and Simulation **3** in red.
