# Supplementary material for: Extended molecular dynamics of a c-kit promoter quadruplex
Source: Nucleic Acids Res. 2015 Oct 10;43(18):8673–93. doi: 10.1093/nar/gkv785 (PMC4605300; doi:10.1093/nar/gkv785)
Supplement: SUPPLEMENTARY DATA [file supp_gkv785_nar-00989-f-2015-File013.pdf]

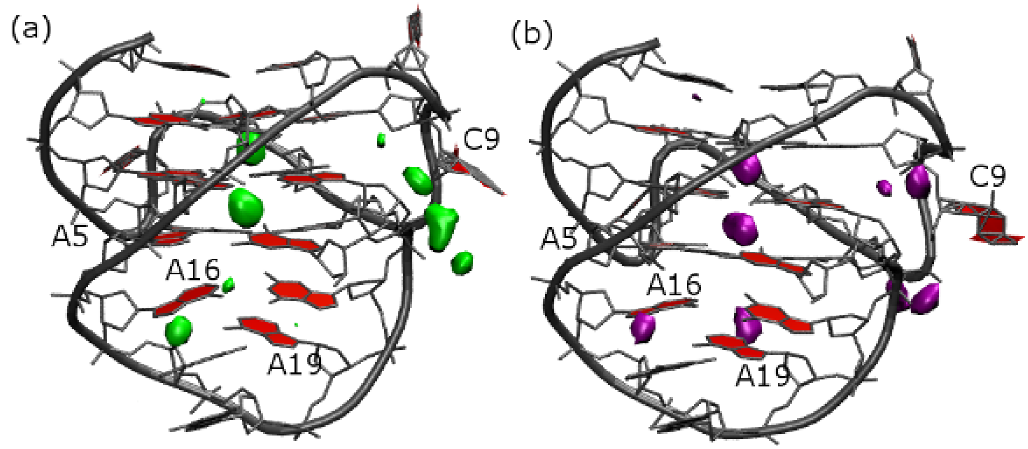

Figure S19: Cation density of (a) Na^+ and (b) K^+ in the Simulations **1** and **3**. The bases are shown in red while the sugar phosphate backbone of the GQ is shown in grey. The Na^+ density is represented in green and the K^+ in purple. The binding sites of the cations are similar and mutually consistent but the occupancies of the cations vary in both simulation. This, however, is not surprising and may still be due to lack of a full convergence of the simulations (note that the binding of ions is affected by loop dynamics).

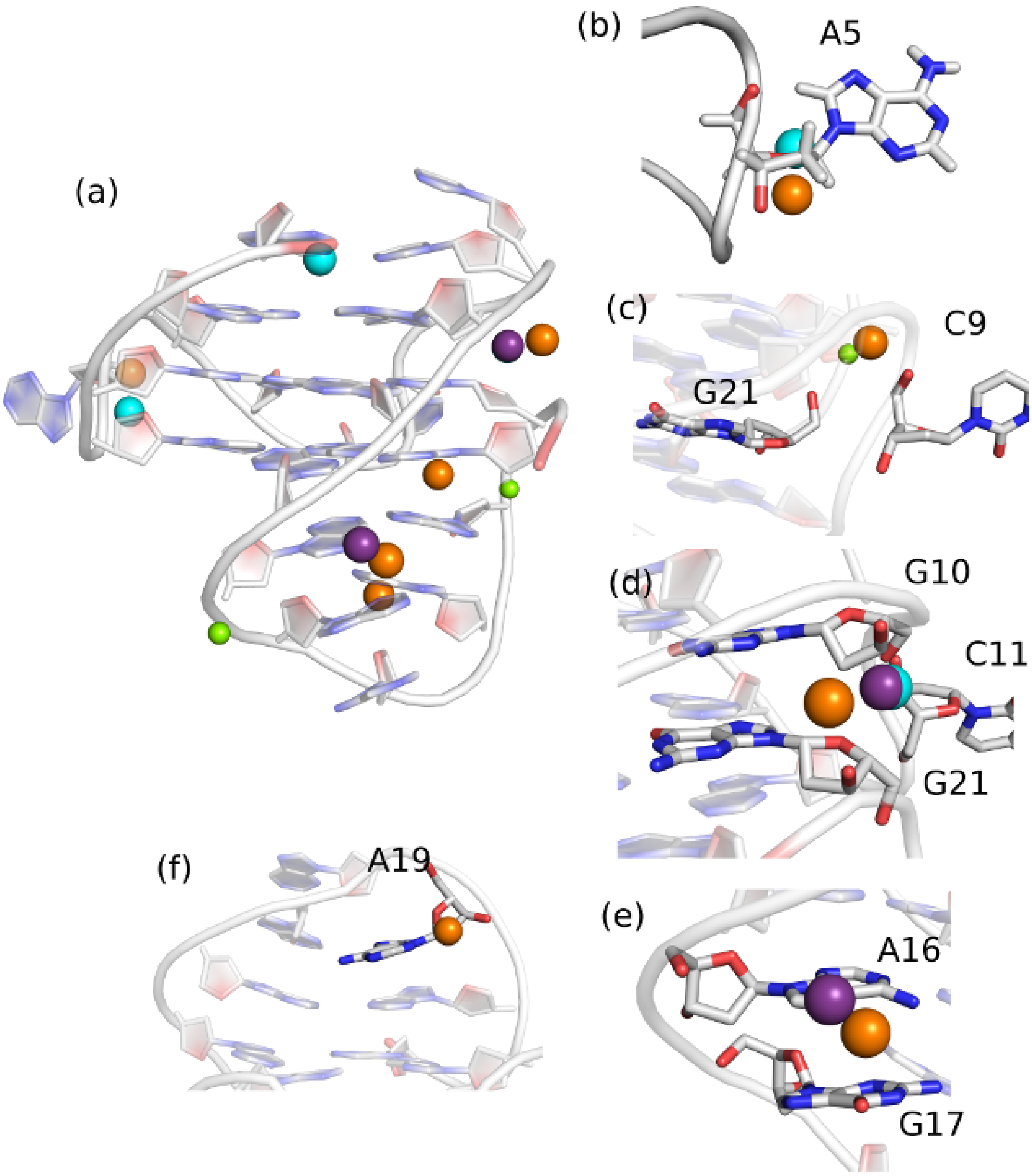

Figure S20: Comparison of cation binding pockets as observed in Simulation **3** and in the crystal structures (PDB id: 3QXR) of the *c-kit* promoter GQ. The K^+ as observed in GQs A and B of the X-ray crystal structure are shown in cyan and purple, respectively. The X-ray Mg^{2+} ions are shown in green. The K^+ in simulation are shown in orange. (a) Cartoon representation of GQ structure in the simulation at ~750ns. The ion positions in crystal structure were overlaid for comparison on the simulation structure using *Chimera.* The cations bind between the (b) O4' of A5 and phosphate of G7, (c) O4' and phosphate groups of C9 and G21 residues, (d) backbone of G10, C11 and G21 residues and (e) A16 and G17 bases, transiently throughout the simulation. (f) Cations also assist repositioning of LP loop by binding to N3(A19). Channel cations are not shown in the Figure.

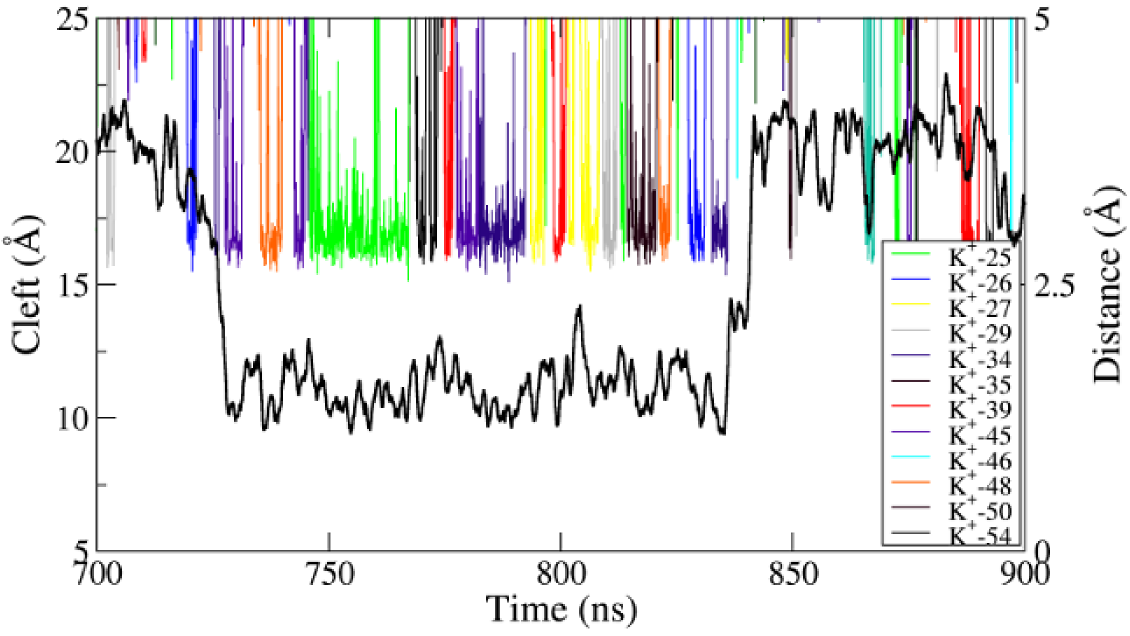

Figure S21: Effect of cation binding to N3 of A19 on the size of the cleft between the LP loop and the adjacent quartet shown in Simulation **3**. The cleft was measured as distance between phosphates of A19 and A5 and is plotted in thick black. The distance between cations and N3(A19) are plotted as colored lines in the graph. The distinct colors indicate different K^+ ions binding to N3(A19). The occupancy of K^+ at N3(A19) was 61% when the cleft was narrow while it was 23% when the cleft was large. It is evident that cation binding facilitates movement of the LP loop towards the third quartet.

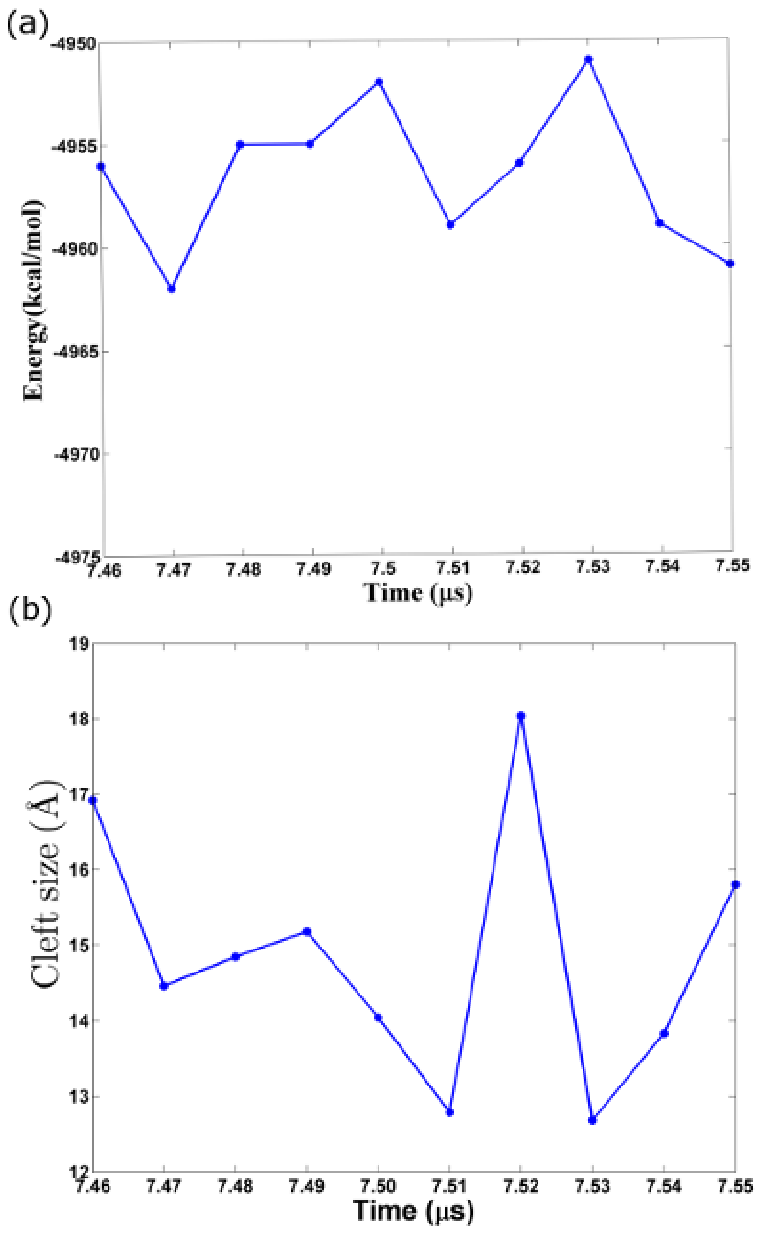

Figure S22: Comparison of energy with the variation in cleft size between the LP loop and the third quartet of *c-kit* promoter GQ. (a) Energy (kcal/mol) vs time plot of *c-kit* promoter GQ between 7.45 and 7.55 μs of Simulation **1** as calculated by MM-PBSA. Each point represents average of 10 ns interval of the trajectory. The entropy values were not used in the energy calculation as their values were almost constant in the ensembles used here. (b) Variation in size of the cleft between LP loop and third quartet brought about by the movement of the loop. The distance between phosphate atom of A19 and phosphate atom of A5 was used to calculate the cleft size. It is evident that the MM-PBSA method is too noisy to reveal any correlation between the loop dynamics and the free energy.

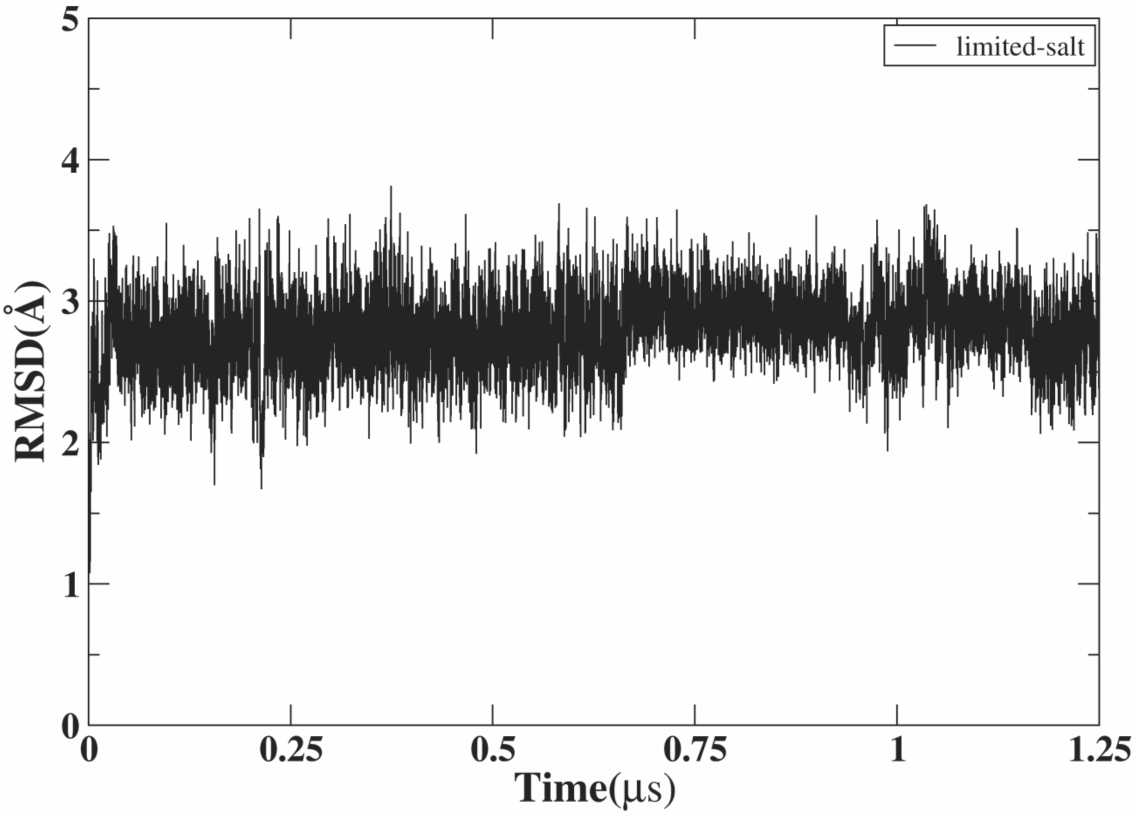

Figure S23: RMSD of backbone atoms of *c-kit* promoter GQ over 1.25 μs in limited-salt simulation carried out in the bsc0χ_{OL4} force field (Simulation **4**).

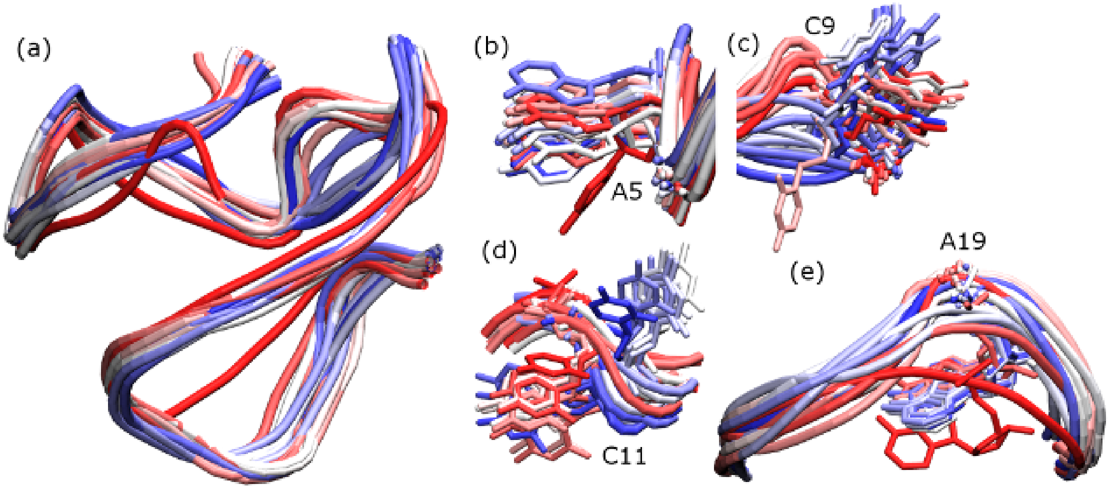

Figure S24: Representation of dynamics of *c-kit* promoter GQ in Simulation **4** carried out in the bsc0χ_{OL4} force field with limited salt for 1.25 µs. The different conformations are colored according to the simulation progression; the structure at the beginning of trajectory is red, the middle in white and the end in blue color. The conformations are sampled at a time step of 50 ns. (a) Overlay of backbone conformations of the GQ sampled over the simulation. The conformations of loop residues (b) A5, (c) C9, (d) C11 and (e) A19 are shown in licorice while the backbone is shown in tube representation.

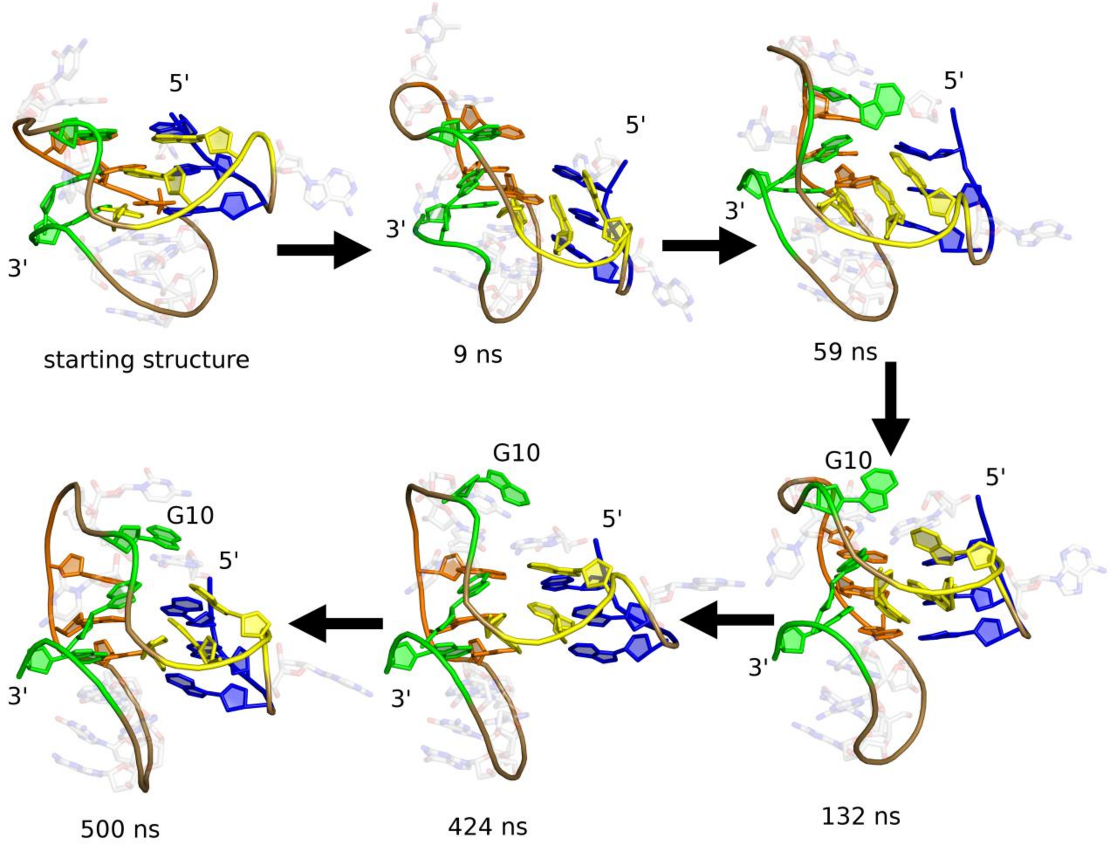

Figure S25: Representation of unfolding pathway of *c-kit* promoter GQ observed in Simulation **6**. The side view of structures observed at various times in the unfolding simulation is shown. The time at which the structure was observed in Simulation **6** is indicated below the respective structure. The G10, and 5' and 3'-ends of the structures are marked in the Figure. The loop backbone is shown in brown. Strand **a** is shown in blue, strand **b** in yellow, strand **c** in green and strand **d** in orange. Stem guanines are shown in cartoon representation while the loop nucleosides are shown as transparent sticks. Hydrogen atoms and ions are not shown.

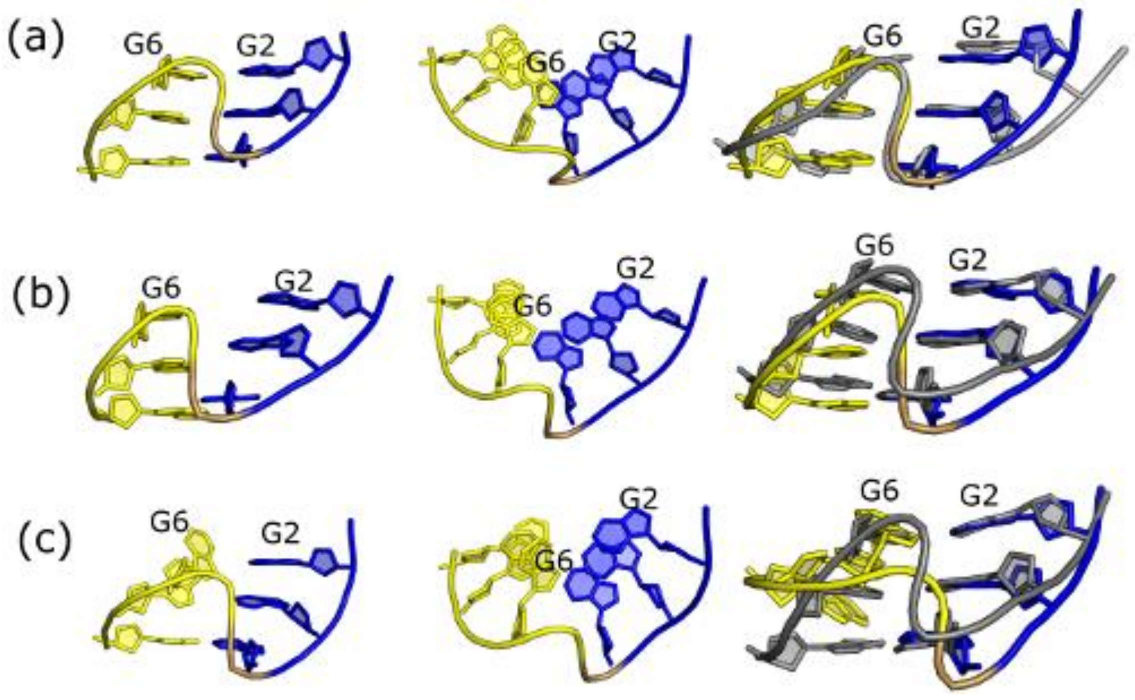

Figure S26: Representation of conformations of propeller loop observed in the present simulations and the criteria we use to judge if the propeller loop is in the native conformation or not. The side view, the top view and overlay with respective loop in GQ B in crystal structure 3QXR is shown. The strands **a** and **b** are shown in blue and yellow, respectively. The loop conformation in crystal structure is shown in grey. G2 and G6 are labelled in the Figure. Only the backbone of single nucleotide propeller loop joining the strands is shown. We say that the loop is in native conformation when the loop backbone and bases (of strands connected by loop) show reasonable alignment with the respective loop and bases of GQ B in the crystal structure. An example of such conformation of propeller loop is shown in (a). We consider the conformation of the propeller loop to be lost when the loop tends to straighten and the bases of the strands are not in hydrogen bonding distances as shown in (b); in this particular case, certain degree of strand slippage (of strand **b**) is seen and G2 – G6 distance increases to more than 4 Å. The conformation is also considered to be lost when the bases of two strands connected by the loop are not coplanar as in (c), i.e., the respective strands are not parallel enough. The cases shown in this Figure can be considered as borderline cases, but we consider them already as showing a loss of native propeller loop conformation.
