# Supplementary material for: Extended molecular dynamics of a c-kit promoter quadruplex
Source: Nucleic Acids Res. 2015 Oct 10;43(18):8673–93. doi: 10.1093/nar/gkv785 (PMC4605300; doi:10.1093/nar/gkv785)
Supplement: SUPPLEMENTARY DATA [file supp_gkv785_nar-00989-f-2015-File014.pdf]

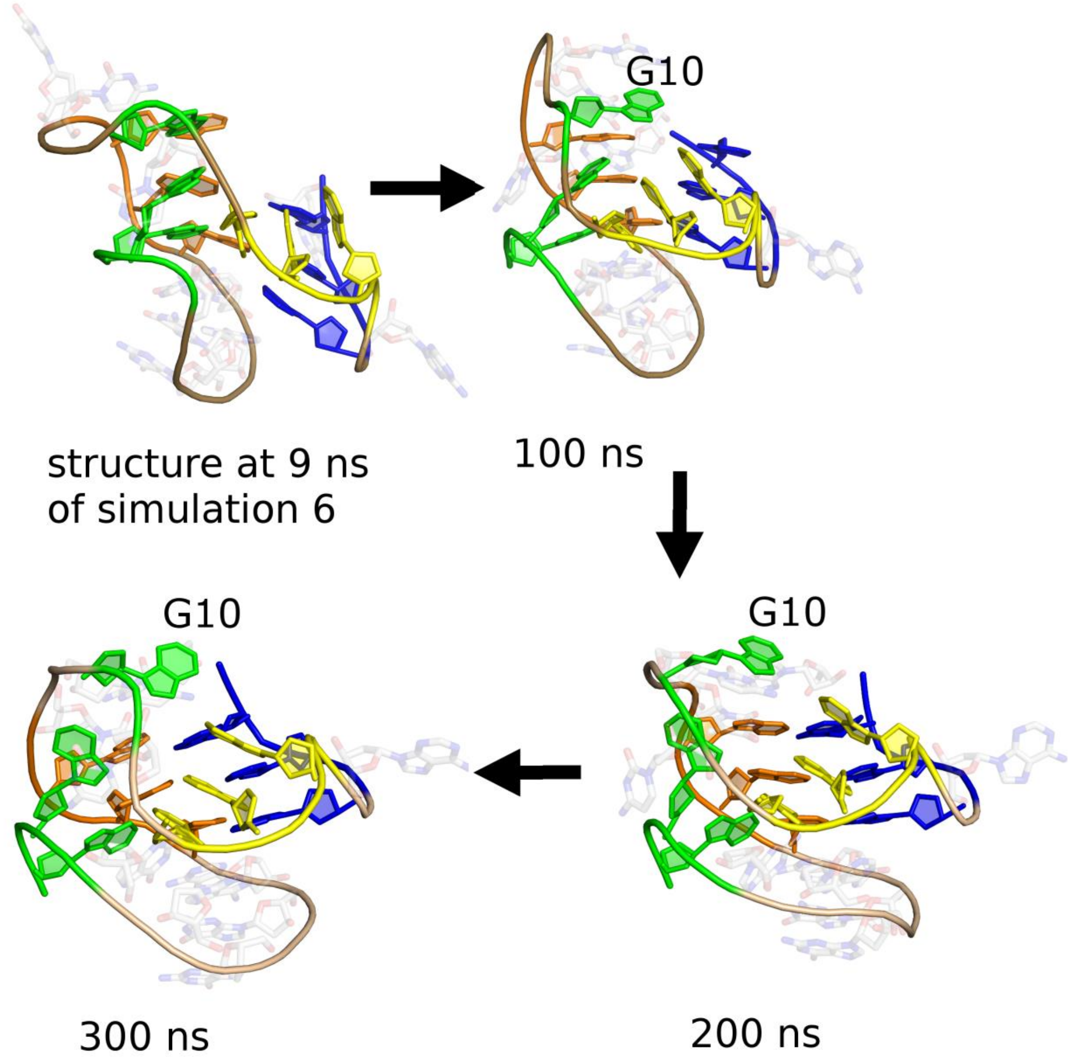

Figure S27: Side view of structures observed in the refolding simulation of perturbed GQ obtained at 9 ns of Simulation **6** (Simulation **7a**). The refolding simulation was carried out for 300 ns in the presence of excess K^+. The side views of structure at the start, 100, 200 and 300 ns of refolding simulation are presented here. The time at which the structure was observed in refolding simulation is indicated below the respective structure. The color scheme is explained in the legend of Figure S25.

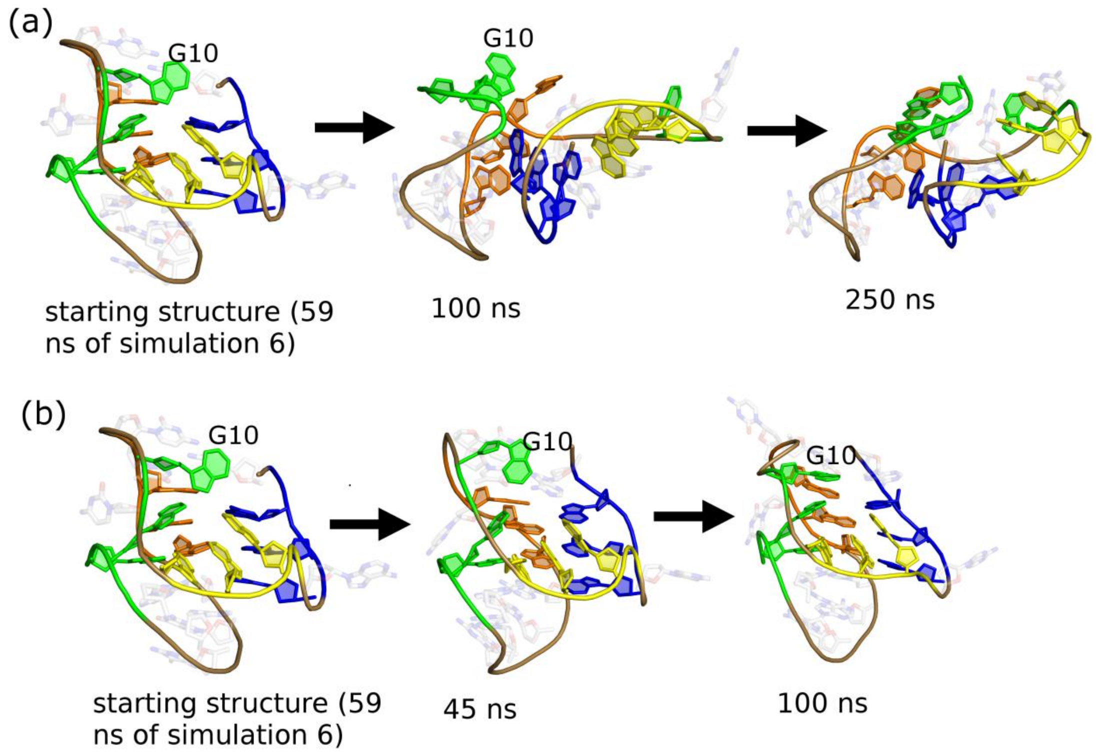

Figure S28: Side view of structures observed in the refolding simulation of perturbed GQ obtained at 59 ns of Simulation **6** (Simulation **7b** and **7c**). Two individual attempts of refolding were carried out. (a) Simulation **7b** was carried out for 250 ns in the presence of excess K^+. The structures at the start, 100 ns and at the end of 250 ns of simulation are presented here. The starting structure showed no sign of refolding in this simulation. (b) Simulation **7c** was carried out for 100 ns also in the presence of excess K^+. The structures at the start, 45 ns and at the end of 100 ns of refolding simulation are presented here. Strand **b** could not realign along the stem axis and no sign of forming the native GQ was evidenced. The color scheme is explained in the legend of Figure S25.

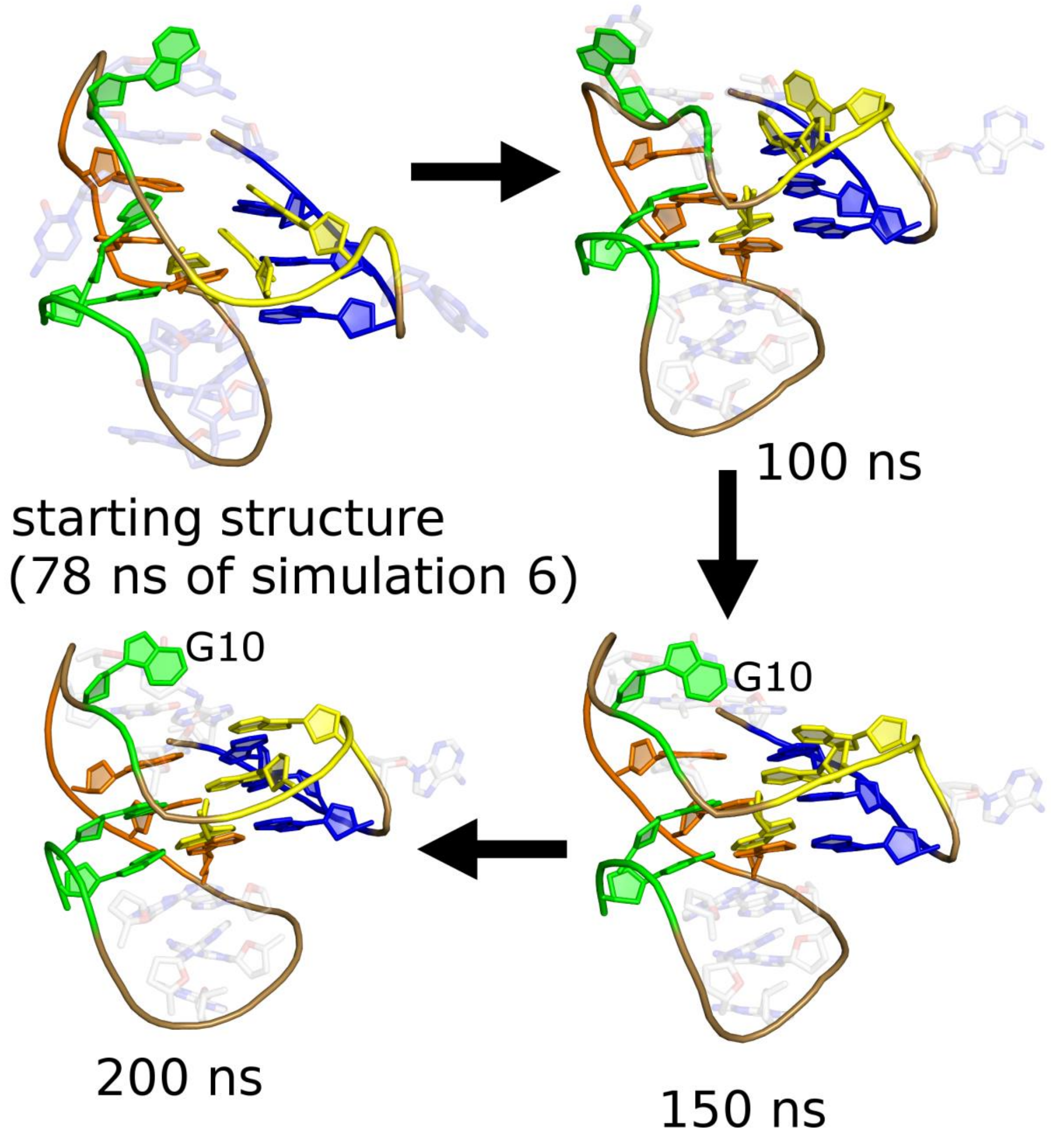

Figure S29: Side view of structures observed in the refolding simulation of perturbed GQ obtained at 78 ns of Simulation **6** (Simulation **7d**). The refolding simulation was carried out for 200 ns in the presence of excess K^+. The side views of structure at the start, 100, 150 and 200 ns of refolding simulation are presented here. The time at which the structure has been observed is indicated below the respective structures. The structure at the end looks quite good at the first sight. The bases of strands **a** and **b** are coplanar, but G2 and G6 remain quite separated, so the structure of the first propeller loop was not re-established. G10 remained extruded from the first quartet. The G10 is marked in the figures and color scheme is explained in the legend of Figure S25.

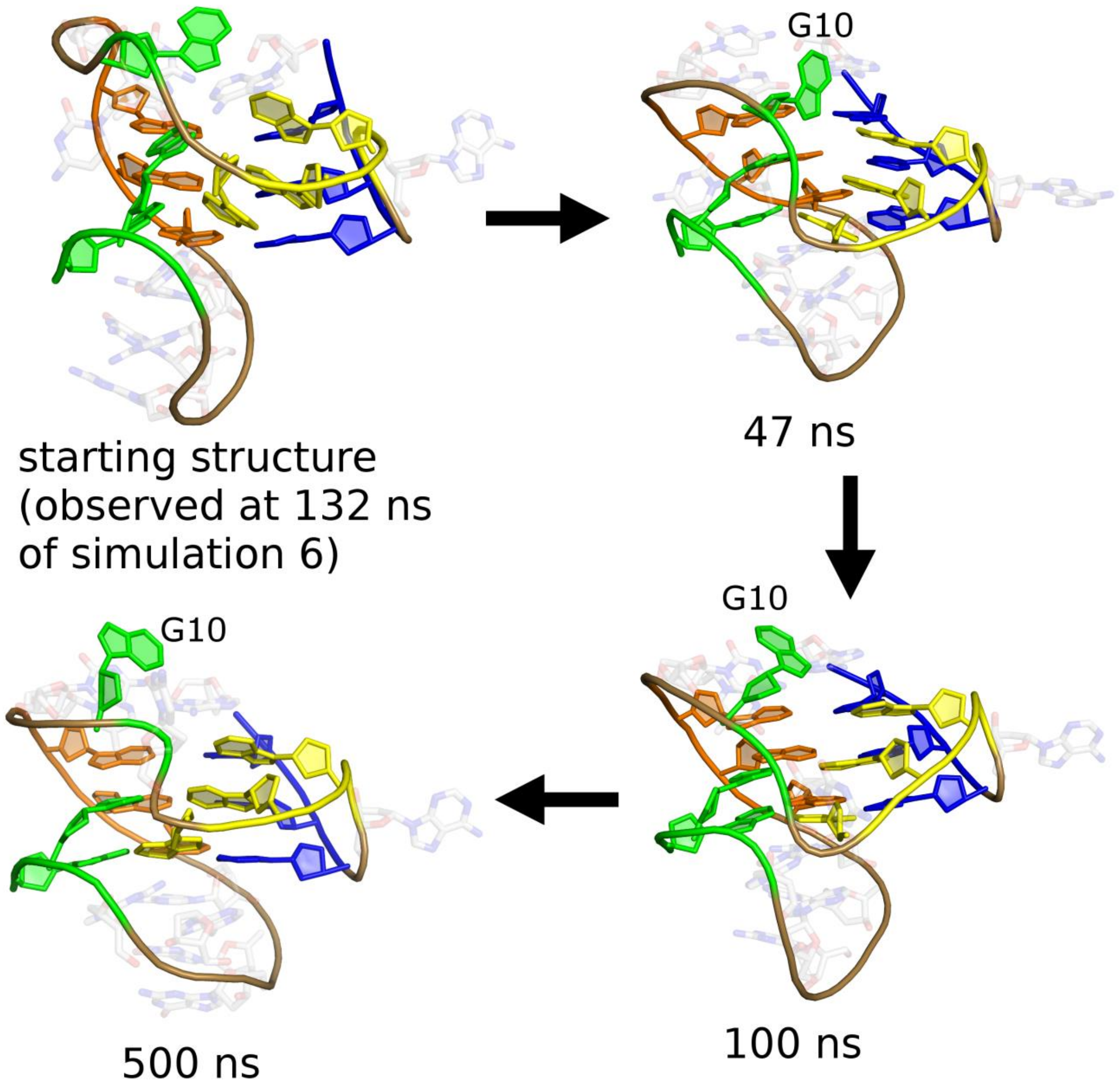

Figure S30: Side view of structures observed in the refolding simulation of perturbed GQ obtained at 132 ns of Simulation **6** (Simulation **7e**). The refolding simulation was carried out for 500 ns in the presence of excess K^+. The side views of structure at the start, 47, 100, 500 ns of refolding simulation are presented here. The time at which the structure has been observed is indicated below the respective structures. The color scheme is explained in the legend of Figure S25. At 40 ns, strands **b** and **c** realign along the G-stem axis and form the native base pairings. The structure is further stabilized so that at 100 ns the GQ almost with native base pairing was obtained. However, G10 could not insert back into the first quartet even after 500 ns and thus the complete GQ could not be reformed. This first propeller loop, however, was reformed.

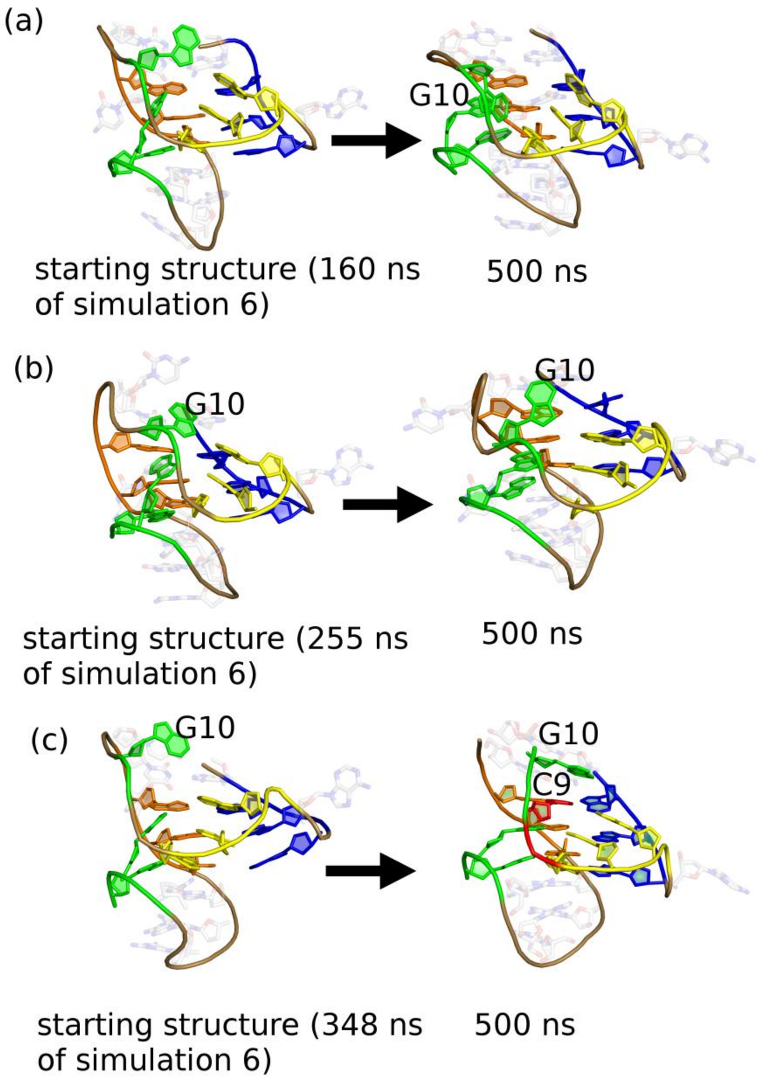

Figure S31: Side view of structures observed in the refolding simulation of perturbed GQ obtained at (a) 160, (b) 255 and (c) 348 ns of Simulation **6** (Simulation **7f**-**h**). The refolding simulation of each of the structures was carried out for 500 ns in the presence of excess K^+. The side views of structure at the start and 500 ns of refolding simulation are presented here. The color scheme is explained in the legend of Figure S25. In all of these simulations, G10 could not insert back into the first quartet and complete GQ could not be reformed. In Simulation **7h**, C9 of the propeller loop inserted into the quartet and formed a misfolded structure. C9 is indicated in red color in part (c) of the Figure. There are also other deformations persisting at the end of the simulations.

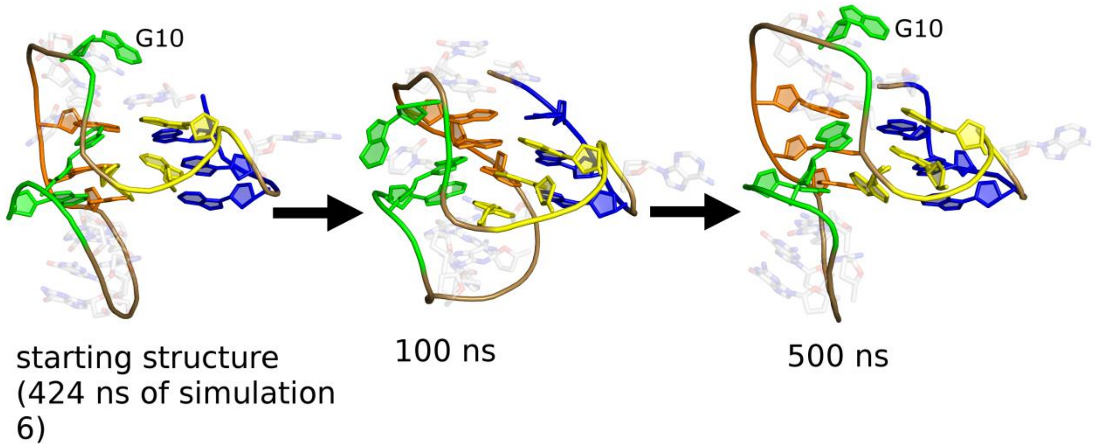

Figure S32: Side view of structures observed in the refolding simulation of perturbed GQ obtained at 424 ns of Simulation **6** (Simulation **7i**). The refolding simulation was carried out for 500 ns in the presence of excess K^+. The side views of structure at the start, at 100 ns and the end of 500 ns of refolding simulation are presented here. The color scheme is explained in the legend of Figure S25. G10 is in *syn* orientation in the starting structure and flips into *anti*-orientation at 340 ns of the refolding simulation. It is still not sufficient to initiate a repair of the structure.

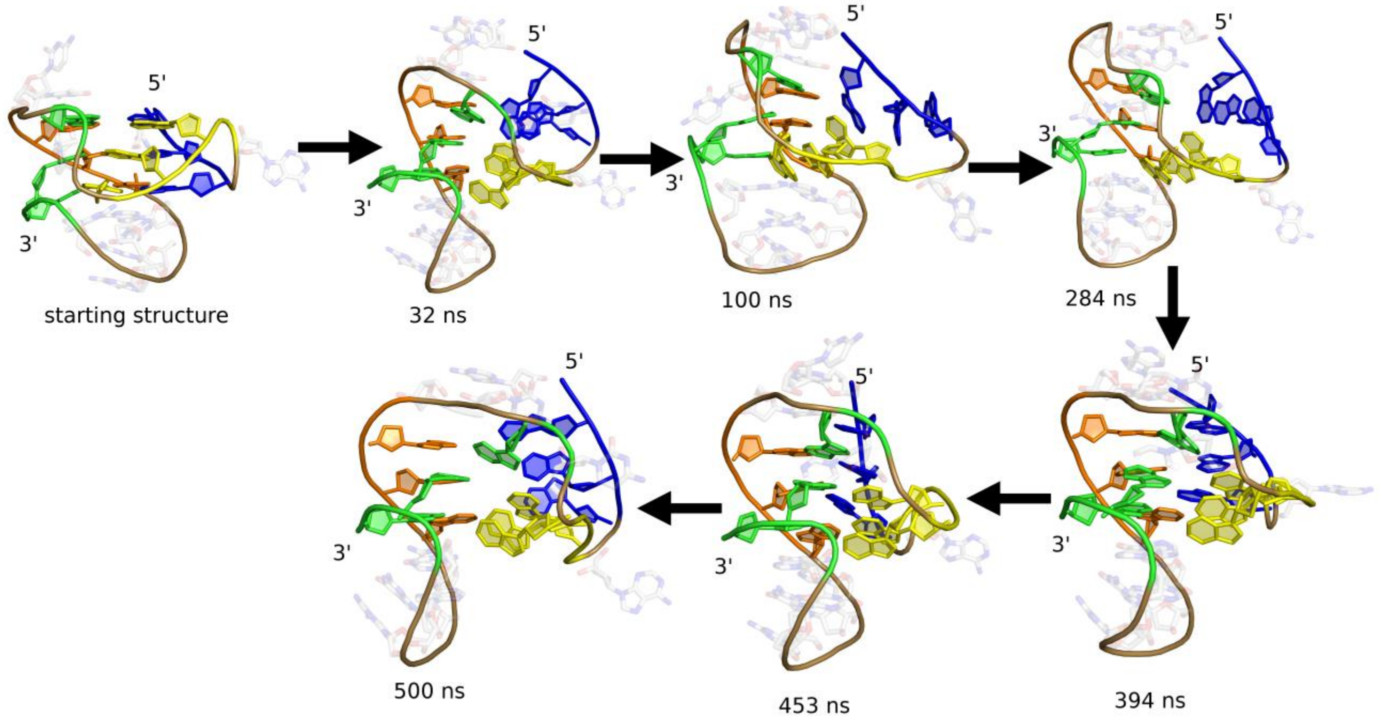

Figure S33: Side view of structures observed at various times in the second unfolding simulation (Simulation **8)**. The time at which the structures were observed in Simulation **8** is indicated below the respective structure. The color scheme is explained in the legend of Figure S25. The 5’ and 3’-end of the structures are marked in the figures.

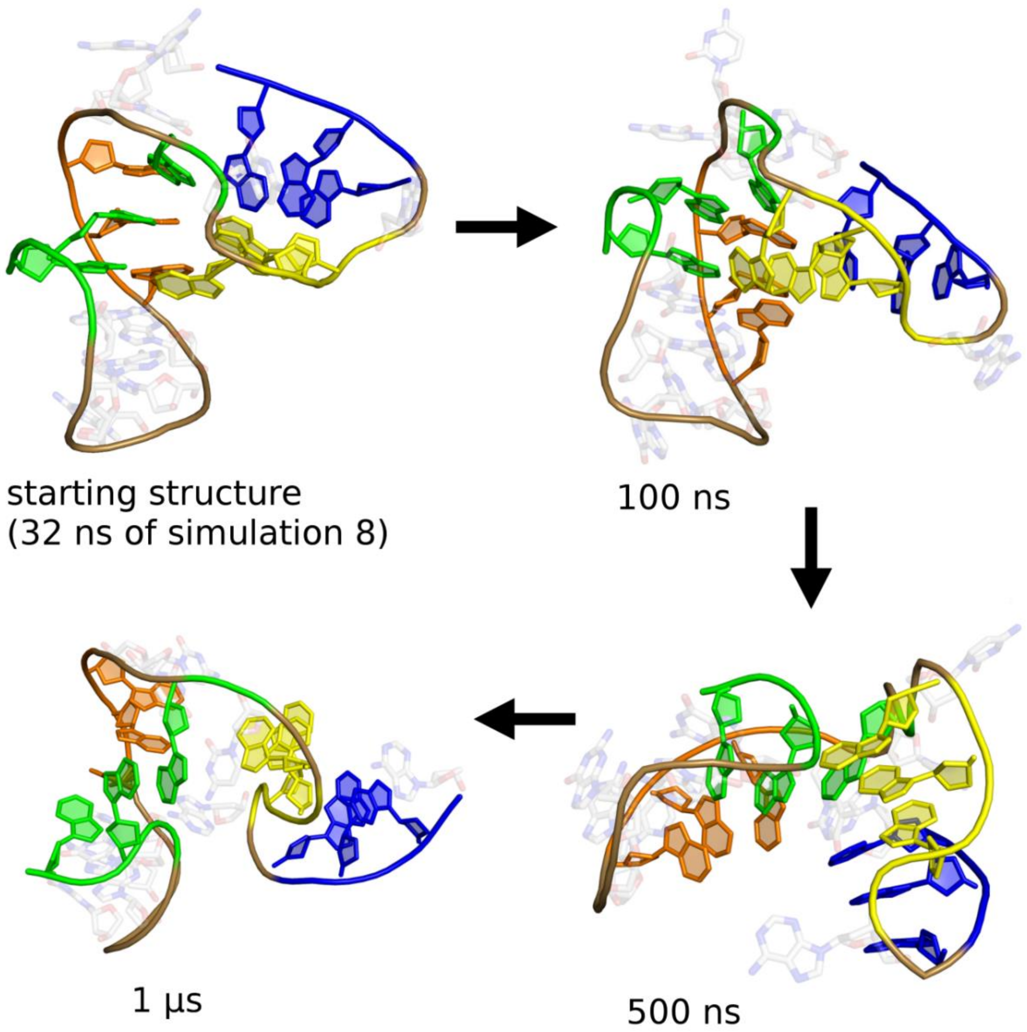

Figure S34: Side view of structures observed in the refolding simulation of perturbed GQ obtained at 32 ns of Simulation **8** (Simulation **9a**). The refolding simulation was carried out for 1 µs in the presence of excess K^+. The side views of structure at the start and after 100 ns, 500 ns and 1 µs of refolding simulation are presented here. The color scheme is explained in the legend of Figure S25. The native interactions of strand **a** are lost. Strand **b** is horizontally aligned along the GQ and is stabilized by non-native hydrogen bonds with bases of strand **c** and **d.** There was no sign of any repair of the GQ in this refolding simulation.

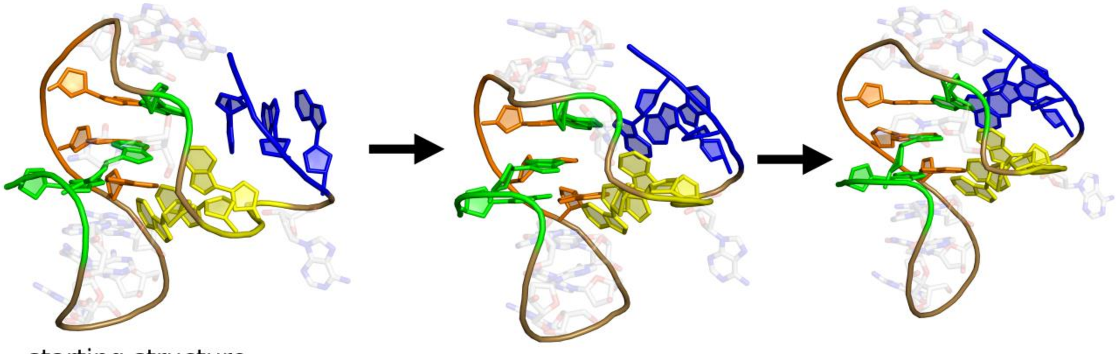

Figure S35: Side view of structures observed in the refolding simulation of perturbed GQ obtained at 100 ns of Simulation **8** (Simulation **9b**). The refolding simulation was carried out for 1 µs in the presence of excess K^+. The side views of structure at the start, 500 ns and 1 µs of the refolding simulation are presented here. The coloring scheme is described in the legend of Figure S25. The strands **a** and **b** could not realign along the stem axis and no refolding was achieved.
