# Supplementary material for: Extended molecular dynamics of a c-kit promoter quadruplex
Source: Nucleic Acids Res. 2015 Oct 10;43(18):8673–93. doi: 10.1093/nar/gkv785 (PMC4605300; doi:10.1093/nar/gkv785)
Supplement: SUPPLEMENTARY DATA [file supp_gkv785_nar-00989-f-2015-File015.pdf]

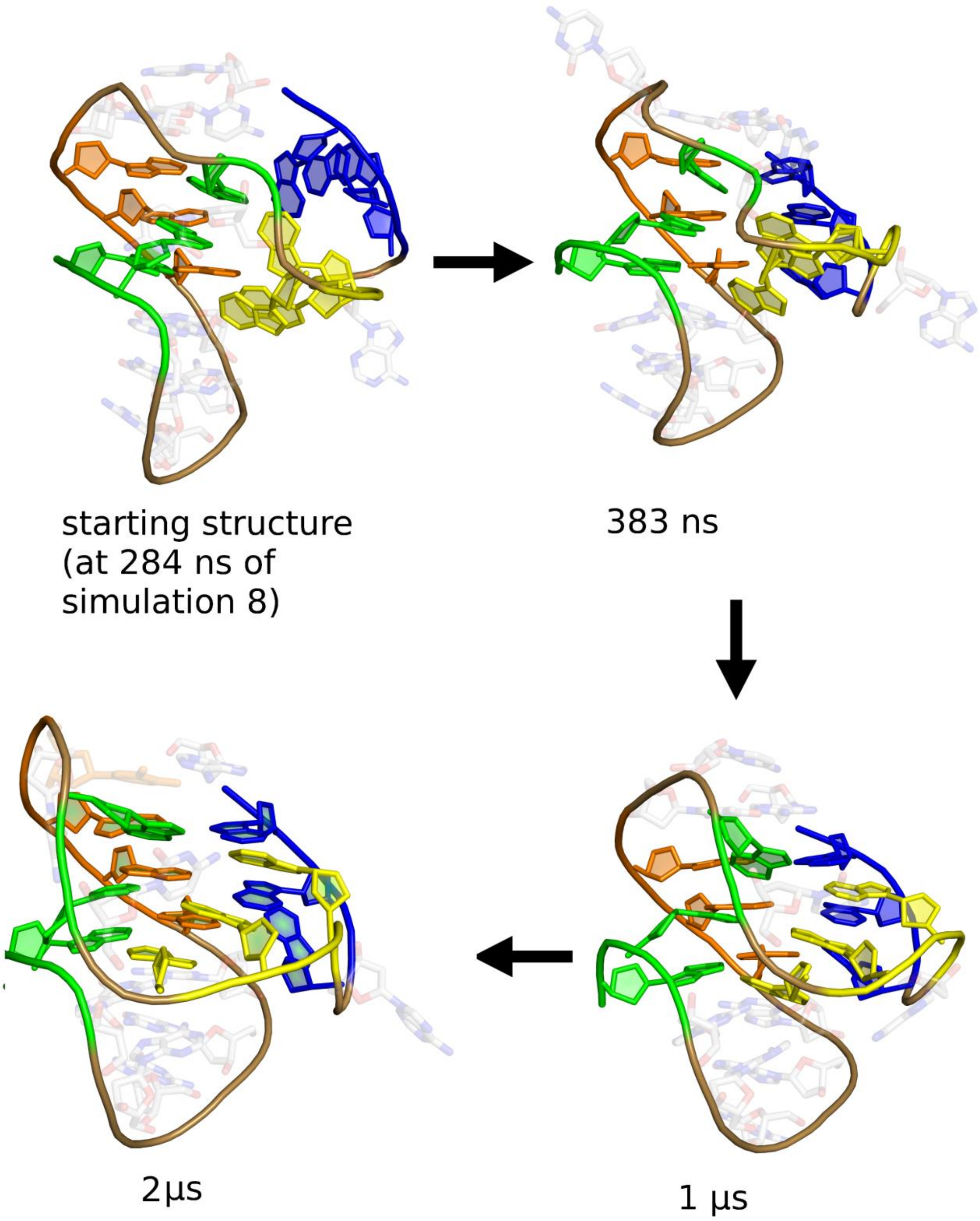

Figure S36: Side view of structures observed in the refolding simulation of perturbed GQ obtained at 284 ns of Simulation **8** (Simulation **9c**). The refolding simulation was carried out for 2 µs in the presence of excess K^+. The side views of structure at the start, 383 ns, 1 µs and 2 µs are presented. The color scheme is explained in the legend of Figure S25. Significant folding from a highly perturbed structure was observed in this simulation. We consider it as the most successful refolding event seen in the whole our study. The native base pairings of quartets 1 and 2 were re-established. The only difference from the native structure is that G4 remained slightly tilted and could not insert into the quartet 3 till the end of 2 µs of this simulation.

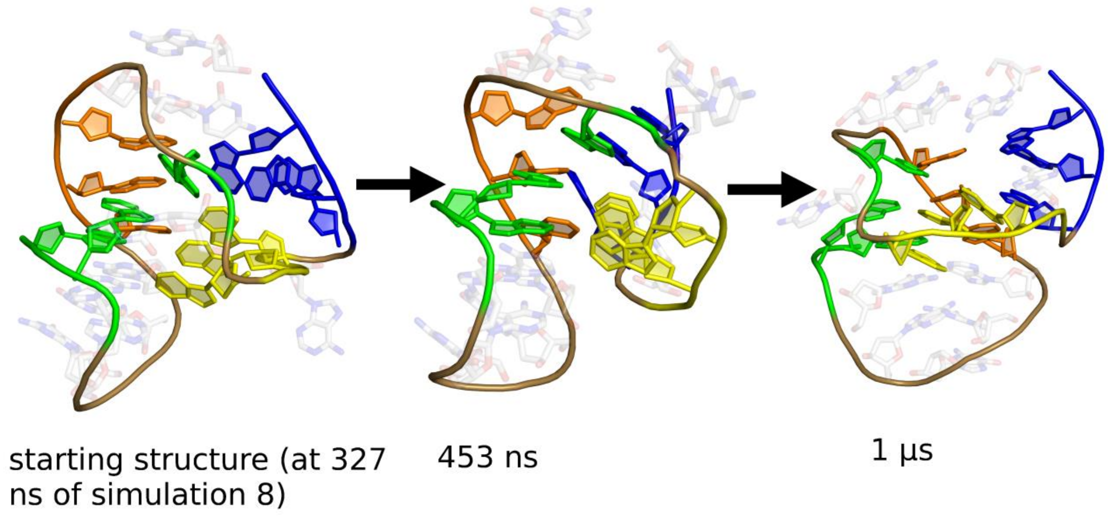

Figure S37: Side view of structures observed in the refolding simulation of perturbed GQ obtained at 327 ns of Simulation **8** (Simulation **9d**). The refolding simulation was carried out for 1 μs in the presence of excess K^+. The side views of structure at the start, 453 ns and after 1 μs of refolding simulation are presented here. The color scheme is explained in the legend of Figure S25.

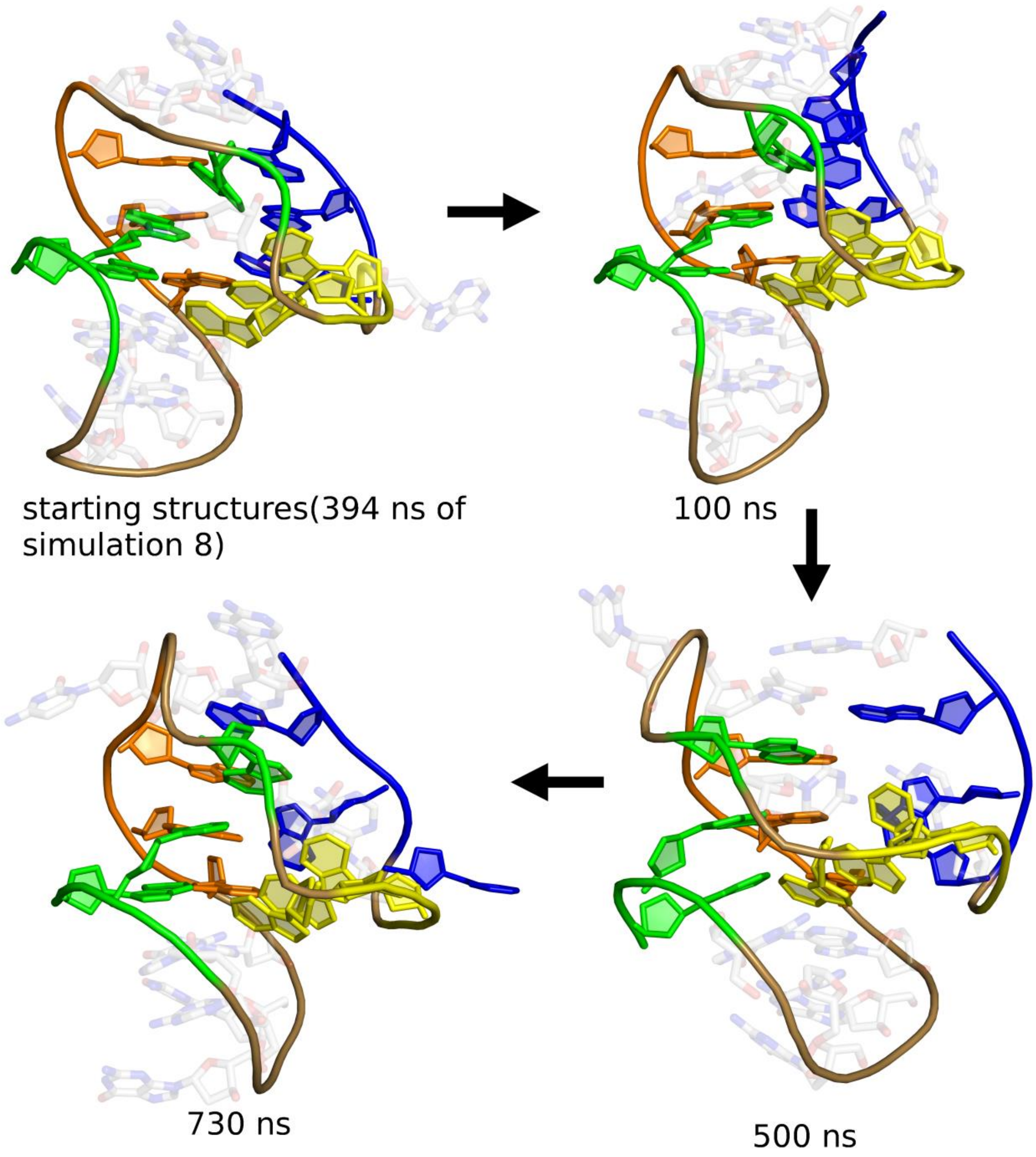

Figure S38: Side view of structures observed in the refolding simulation of perturbed GQ obtained at 394 ns of Simulation **8** (Simulation **9e**). The refolding simulation was carried out for 730 ns in the presence of excess K^+. The side views of structure at the start 100, 500 and after 730 ns of refolding simulation are presented here. The color scheme is explained in the legend of Figure S25.

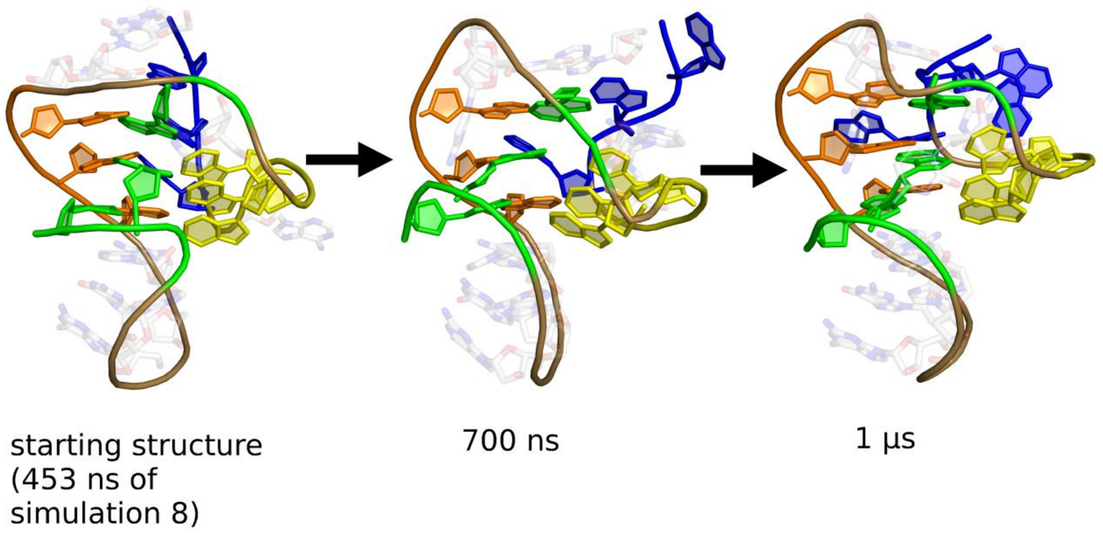

Figure S39: Side view of structures observed in the refolding simulation of perturbed GQ obtained at 453 ns of Simulation **8** (Simulation **9f**). The refolding simulation was carried out for 1 µs in the presence of excess K^+ ions. The side views of structure at the start, 700 ns and 1 µs of refolding simulation are presented here. The color scheme is explained in the legend of Figure S25.

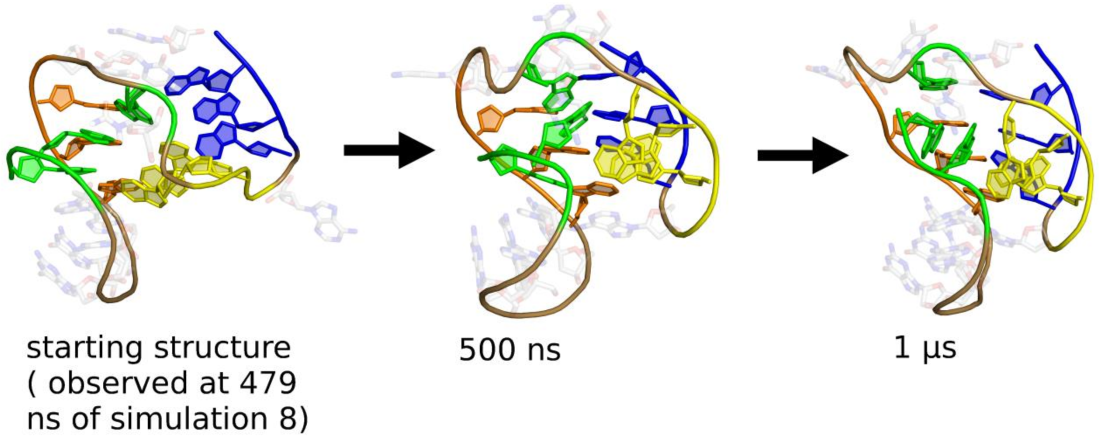

Figure S40: Side view of structures observed in the refolding simulation of perturbed GQ obtained at 479 ns of Simulation **8** (Simulation **9g**). The refolding simulation was carried out for 1 µs in the presence of excess K^+ ions. The side views of structure at the start, 500 ns and after 1 µs of refolding simulation are presented here. The color scheme is explained in the legend of Figure S25.

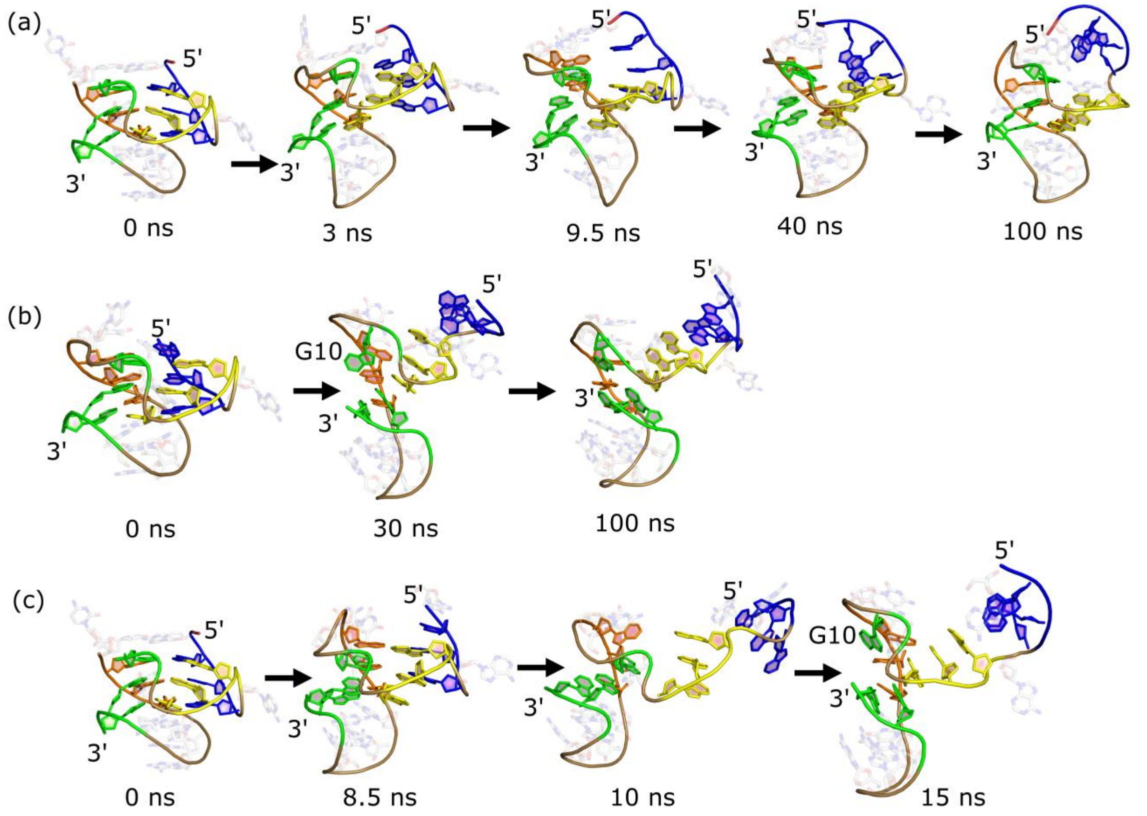

Figure S41: The unfolding pathway of *c-kit* promoter GQ observed by additional independent no-salt simulations (Simulations **10a-c**). See legend to Figure S25 for further details. Unfolding in all three simulations is initiated by the loss of conformation of single nucleotide propeller loops, leading to rotation and expulsion of the strand **b**. (a) The structures of lateral and LP loops are also lost in the Simulation **10a**, albeit it is not apparent from the scheme. (b) Strand **a** moves far away from the rest of the structure in the Simulation **10b.** (c) The conformation of both single nucleotide propeller loops is again lost within 15 ns of the Simulation **10c**.

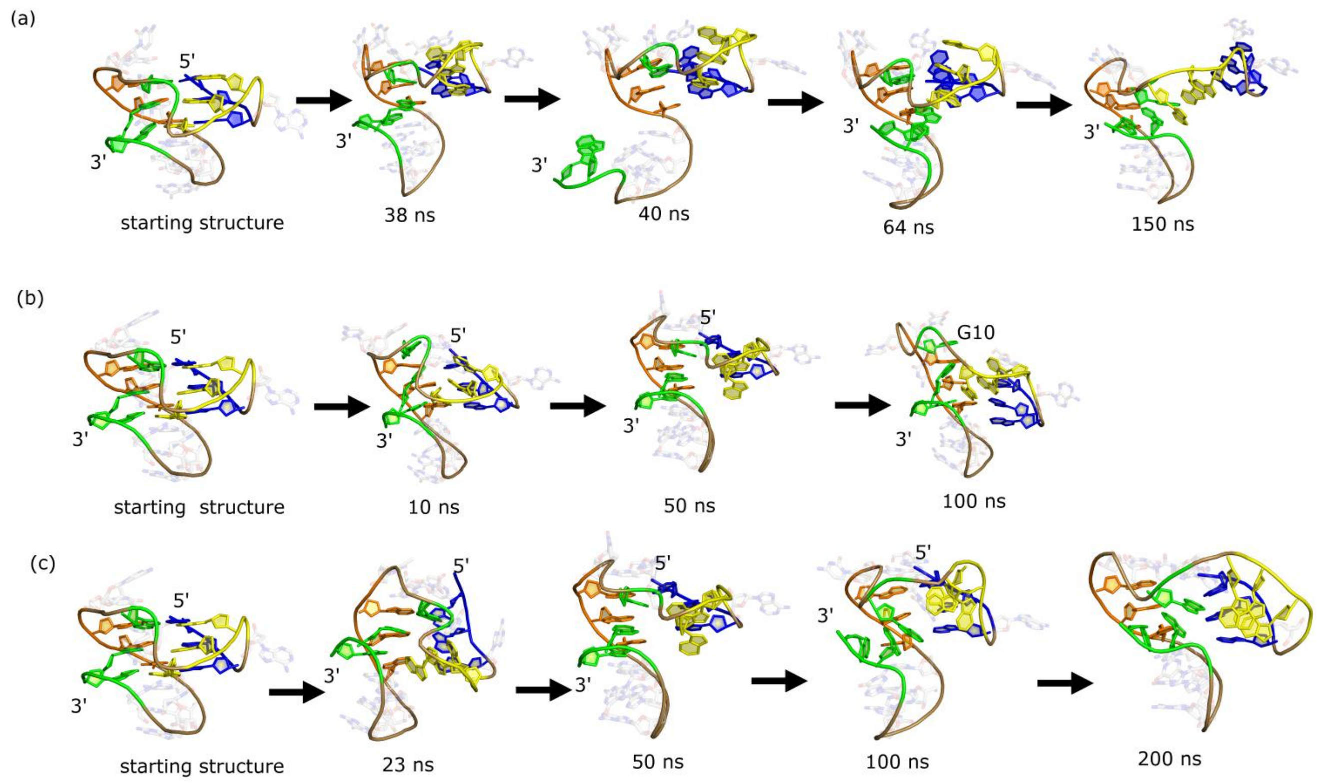

Figure S42: Unfolding pathway of *c-kit* promoter GQ in SPC/E water model (Simulations **11a-c**). Simulations **11a**, **11b** and **11c** were carried out for 150ns, 100ns and 200 ns respectively. The strands **a** and **b** are expelled out from G-stem axis similar to Simulations **6**, **8** and **10.** The color scheme is explained in legend of Figure S25.

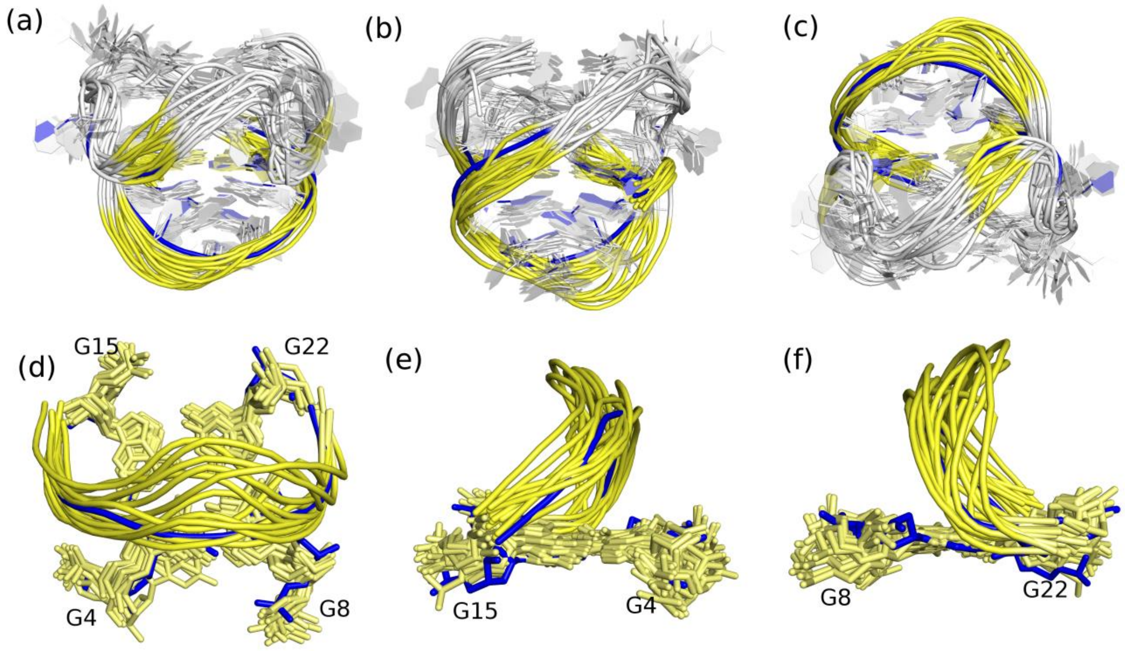

Figure S43: Representation of different loop conformations sampled in 10 µs long Simulation **1**. (a-f) show loop conformations from different perspectives. In (d to f) the quartet adjacent to LP loop (third quartet) is shown in sticks while the backbone of LP loop is shown in cartoon representation. Third quartet and backbone of LP loop sampled in Simulation **1** are shown in yellow color. The conformation in the starting structure is shown in blue.

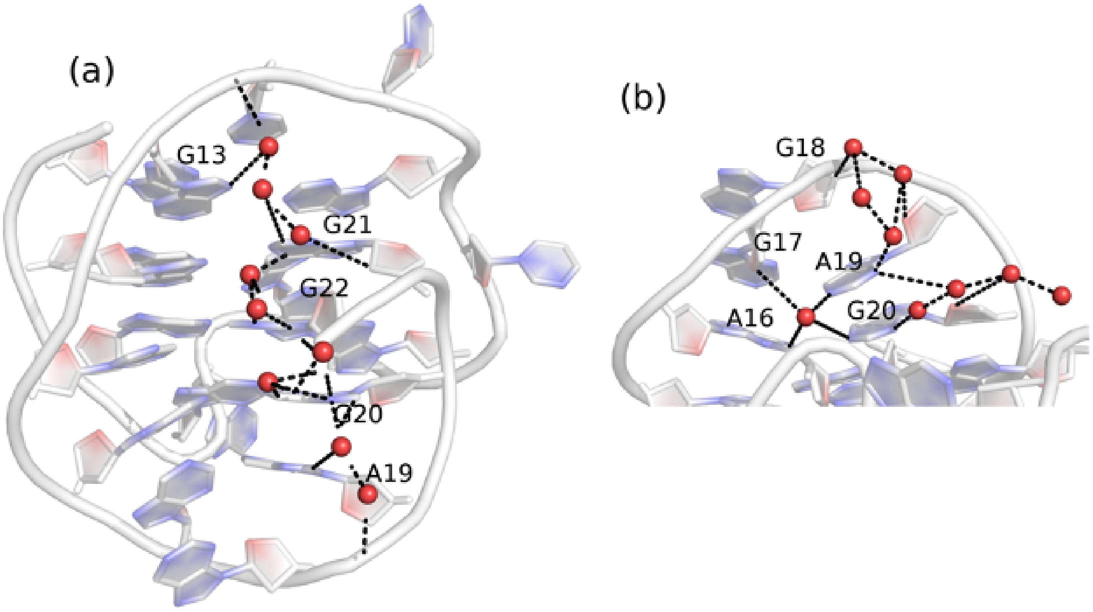

Figure S44: Representation of hydration pattern observed in *c-kit* promoter GQ in Simulation **1**. The GQ is shown in cartoon representation and water molecules are shown in red. The black lines indicate hydrogen bond interactions. (a) The water molecules bridge the LP loop to the adjacent quartet which is in turn linked to the quartets below to form a spine of hydration. (b) The electronegative atoms of LP loop act as a scaffold for the binding of water molecules.
